# Constraints on cooperation shape hierarchical versus distributed structure in human groups

**DOI:** 10.1038/s41598-022-23454-9

**Published:** 2023-01-20

**Authors:** Matthew R. Zefferman

**Affiliations:** 1 National Institute for Mathematical and Biological Synthesis, Knoxville, TN 37996 USA; 2 Naval Postgraduate School, Monterey, CA 93943 USA

**Keywords:** Anthropology, Cultural evolution, Social evolution

## Abstract

Some human groups are organized hierarchically and some are distributed. Both types of groups occur in economic, political, and military domains, but it is unclear why hierarchical organizations are favored in certain contexts and distributed organizations are favored in others. I propose that these different organizational structures can be explained by human groups having different constraints on their ability to foster cooperation within the group. Human within-group cooperation is often maintained by monitoring and punishment. In hierarchical groups, monitoring and punishment are organized into tree-like command-and-control structures with supervisors responsible for monitoring the cooperation of their subordinates and punishing non-cooperators. By contrast, in distributed groups, monitoring is diffuse and punishment is collective. I propose that the organization of cooperative human groups is constrained by the costs of monitoring and punishment. I formalize this hypothesis with a model where individuals in a group cooperate to produce public goods while embedded in a network of monitoring and punishment responsibilities. I show that, when punishment costs are high and monitoring costs are low, socially-optimal monitoring and punishment networks are distributed. The size of these distributed networks is constrained by monitoring costs. However, when punishment costs are low, socially-optimal networks are hierarchical. Monitoring costs do not constrain the size of hierarchical networks but determine how many levels of supervision are required to foster cooperation in the hierarchical group. These results may explain the increasingly large and hierarchical groups throughout much of human history. They also suggest that the recent emergence of large-scale distributed organizations has been possible because new technologies, like the internet, have made monitoring costs extremely low.

## Introduction

**Figure 1:**
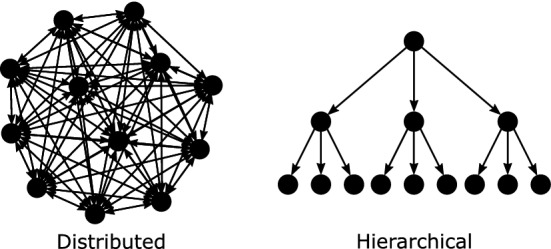
Example groups with distributed (left) and hierarchical (right) organizational structures.

Why are some human social organizations hierarchical and others distributed? This question has engaged scholars of political^1–13^, economic^14–20^, and military^21–24^ organizations. For example, anthropologists and archaeologists ask why smaller-scale distributed societies sometimes transition to hierarchical chiefdoms or even more hierarchical states^5,7^. Organizational economists explore the benefits and challenges of hierarchical organization in economic firms^14,16,18,20^. Political scientists grapple with how to best structure organizations to foster international cooperation^9–11^. Military practitioners try to understand which type of organization is better to achieve specific military objectives^21–23^. Both types of organizational structures can be represented as directed networks (Fig. 1).

Political, economic, and military organizations all need to foster cooperation in groups, which is notoriously difficult when individual costs are paid by the cooperator, but the benefits are distributed widely to group members. Consider the classic collective action problem of warfare, where participants incur high risk, often including death, for benefits that may be widely distributed^25^. Organizations supporting cooperation in warfare can be distributed or hierarchical^21–23^. For example, Turkana pastoralists in East Africa who engage in lethal cattle raids with neighboring groups. These raids are high risk, with about half of adult male mortality due to raiding^26^. Although an individual’s active participation in a raid increases the group’s probability of success, shirkers are less likely to die or be injured than active participants and can also capture more cattle while others are busy fighting^24,26,27^. Shirking lowers a warrior’s expected costs and increases his chance of bringing home cattle. However, there is a cooperative dilemma because, if all warriors shirk, the raid will fail without anyone capturing cattle^24,26,27^.

Cooperation on Turkana cattle raids is maintained by distributed institutions of monitoring and punishment^24,26,27^. Small raids often do not have leaders. Each individual is monitored by other participants and shirkers are collectively punished. Larger raids often have leaders who coordinate strategy. However, monitoring and punishment are still distributed since they do not have coercive authority over others^24,28^. Raiders are still collectively monitored by others of a similar age who also punish any shirkers in their age group. Punishment includes severe beatings, social exclusion, or the forfeit of livestock^24,26,27^.

By contrast, militaries in large-scale industrialized societies are famously hierarchical with cooperative institutions organized in tree-like command-and-control structures.^21–23^ Individuals higher up in the tree are responsible for monitoring and, if necessary, sanctioning those below them. An advantage of this type of organization is that it is efficient, with clear lines of authority and “unity of command”. Another advantage is that tree-like structures can be nested and grow by adding more subordinate branches to the hierarchy.^8,12^ Tree-like structures are also ubiquitous in large-scale political^5–7^ and economic^19,20^ organizations.

Similarly, consider the collective action problem of organizing and disseminating knowledge^29^. The governance of the online encyclopedia, Wikipedia, to a more traditional encyclopedia, *The Encyclopædia Britannica*, is another contrast between distributed and hierarchical organization. Wikipedia is maintained by thousands of volunteer contributors and hundreds of editors who update the context, ensure that it conforms to the website’s standards, and monitor content for violations or vandalism by other contributors^30^. Because all updates to Wikipedia’s content are opened and logged, monitoring the site for violations of cooperative norms is relatively easy and violators can be punished by banning their user accounts. Banning a user account is accomplished through the consensus of the Wikipedia community with a smaller board arbitrating disputes and handling high-profile cases.

The publication of *The Encyclopædia Britannica*, which had fifteen editions published over two centuries, was managed by a much more hierarchical organization with a professional editor who oversaw editorial staff who solicited articles from a relatively small number of experts. The editors were nested in a tree-like structure with subordinate editors reporting to managing editors who monitored the content created by contributors.

Despite the differences in organizational structure, Wikipedia and *Encyclopædia Britannica* had similar accuracy for scientific articles as far back as 2005^31^. Though *The Encyclopædia Britannica* is no longer published as a print encyclopedia, it persists as a web portal and phone application. One reason why Wikipedia has persisted and *Encyclopædia Britannica* has not may be because of the relatively inexpensive monitoring costs associated with a free open digital platform and a large number of volunteers willing to provide collective sanctioning of wrong-doers.

Both distributed and hierarchical organizations use monitoring and punishment of non-cooperators to incentivize cooperation^13^. “Monitoring” involves observing others for violations of cooperative norms. However, monitoring is costly, taking resources that could otherwise be contributed directly to the public good or retained by the individual. Consequently, the problems of how to pay for monitoring^16^ and how to efficiently structure it within organizations^17,20^ have been a focus of organizational economics^19^. However, these models typically presuppose that organizations of interest will have tree-like structures. The model in this paper shows how monitoring costs influence the optimal design of the organizational structures themselves.

“Punishment” involves imposing costs selectively on non-cooperators^32–34^. Understanding how punishment incentivizes cooperation has been a focus of social and biological scientists, who have found that it is effective, especially when non-cooperation is rare^33,35^. However, when punishment is costly for the punisher, it is a “second-order” collective action problem since individuals who do not pay the cost of punishing non-cooperators have higher payoffs than those who do^34^. Punishment must be “incentive compatible”, in that punishers must have an incentive to punish^9^. Models of costly punishment have shown that it is more effective when coordinated (e.g.,^36^), non-anonymous (e.g.,^37^), or centralized (e.g.,^11,34,38,39^). Experimental evidence suggests that costly punishment can increase collective action in both large-scale^40,41^ and small-scale^42,43^ societies. Similar to models of monitoring, models of punishment typically assume fixed organizational structures with punishment either dyadic or distributed within groups. The model in this paper shows how punishment costs influence the optimal design of the organizational structures themselves.

This paper examines how optimal organizations are more distributed or more hierarchical depending on the efficiencies of cooperation, monitoring, and punishment. Previous related models of hierarchy and cooperation have assumed hierarchical or distributed organizational structures *a priori* to examine the conditions where people would prefer hierarchical institutions^6^, how hierarchical societies generate inequality between subordinates and managers^44^, and how hierarchical societies can grow through inter-group competition and warfare^12,45^. The model in this paper does not assume a particular organizational structure. Organizations are modeled as networks which can be completely distributed, completely hierarchical, or somewhere in between.

## The model

**Figure 2:**
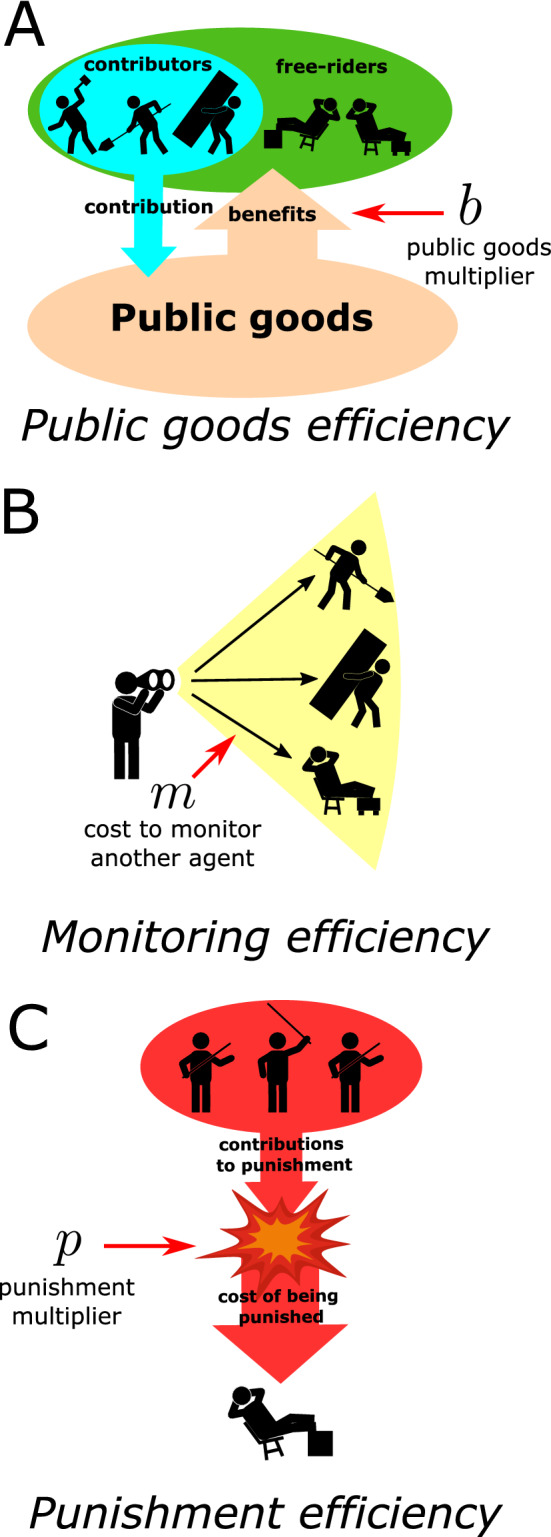
The three efficiencies that determine optimal network structure. An individual’s contribution to a public good is multiplied by the public goods efficiency, *b*, and the result is distributed equally to other group members. Monitoring efficiency, *m*, is the cost for one individual to effectively monitor another. An individual’s contribution to punishment is multiplied by the punishment efficiency, *p*, and the result is subtracted from the punished individuals’ payoffs.

Suppose *N* individuals play a linear public goods game where they can contribute to a public good. Contributions to the public good are multiplied by a “public goods efficiency,” $b \ge 1$, with the product distributed equally to all other individuals (Fig. 2A). This creates a social dilemma because, even though everyone is better off if everyone contributes to the public good, in the absence of institutions that promote contributions, an individual is always better off if they do not contribute.

However, suppose individuals can monitor others and punish those who do not contribute. Further suppose that each individual is assigned an “institutional role” that determines who they are responsible for monitoring and, if necessary, punishing. In the model, these institutional roles are represented by a directed network where nodes represent individuals and links designate individuals’ responsibilities for monitoring and punishing other individuals. Specifically, a directed link from individual *i* to individual *j*, $\ell _{ij} = 1$, indicates that individual *i* is responsible for monitoring individual *j* and punishing individual *j* if individual *j* is not a contributor. The lack of a directed link, $\ell _{ij} = 0$, indicates that individual *i* has no responsibilities for monitoring individual *j*. I define $k_{in,i} = \sum _j \ell _{ji}$ as individual *i*’s *in-degree*, the total number of links directed towards individual *i*. I define $k_{out,i} = \sum _j \ell _{ij}$ as individual *i*’s *out-degree*, the number of links directed away from individual *i*. Therefore, $k_{in,i}$ is the number of individuals who are responsible for monitoring individual *i* and $k_{out,i}$ is the number of individuals that individual *i* is responsible for monitoring. I define *K* as the total number of directed links in the network, that is $K = \sum _i \sum _j \ell _{ij}$.

In the model, monitoring is costly. The cost for an individual to effectively monitor another is 1/*m*, where *m* is the “monitoring efficiency” (Fig. 2B). For example, if $k_{out,i} = 3$, individual *i* is responsible for monitoring three other individuals for a total monitoring cost of 3/*m*. Higher monitoring efficiency implies that individuals can effectively monitor more individuals for the same cost.

Punishing is also costly, with the cost determined by a “punishment efficiency” parameter, *p*. Any resources an individual allocates to punishment are multiplied by the punishment efficiency, with the result subtracted from the punished individual’s payoff (Fig. 2C). Higher punishment efficiency implies that the cost to the punisher is lower relative to the cost to the punished.

Individuals can be considered either “contributors” or “free-riders”. Contributors contribute at least a total threshold amount, $c_t$, to a combination of monitoring, punishment, and the public good following their institutional role as determined by their location in the network. A contributor’s first priority is to pay for any monitoring and punishment responsibilities up to the $c_t$ threshold. They are then responsible for contributing anything remaining under the $c_t$ threshold directly to the public good. All non-contributors are considered free-riders.

Any individual with punishment responsibilities is additionally expected to hold an amount, $c_p \le c_t$, in reserve to use for any punishment. If they monitor any free-riders, they are expected to allocate this punishment equally to punish any free-riders they monitor. If none of the individuals that they monitor are free-riders, they are expected to then contribute the full $c_p$ directly to the public good. If $k_{out,i} = 0$, individual *i* has no monitoring or punishment responsibilities and is expected to contribute the entire threshold amount to the public good. A summary of all model parameters is in Table 1.

**Table 1:** Summary of model parameters.

Group/network parameters		Individual parameters	
*N*	Number of individuals/nodes	$k_{in,i}$	Number of links directed to individual *i*
*K*	Number of directed links	$k_{out,i}$	Number of links directed from individual *i*
$\Pi _N$	Group payoff/social efficiency	$\pi _i$	Individual *i*’s payoff

Game parameters		Institutional parameters	
*b*	Public goods efficiency	$c_t$	Contribution threshold
*m*	Monitoring efficiency	$c_p$	Punishment reserve amount
*p*	Punishment efficiency		

**Figure 3:**
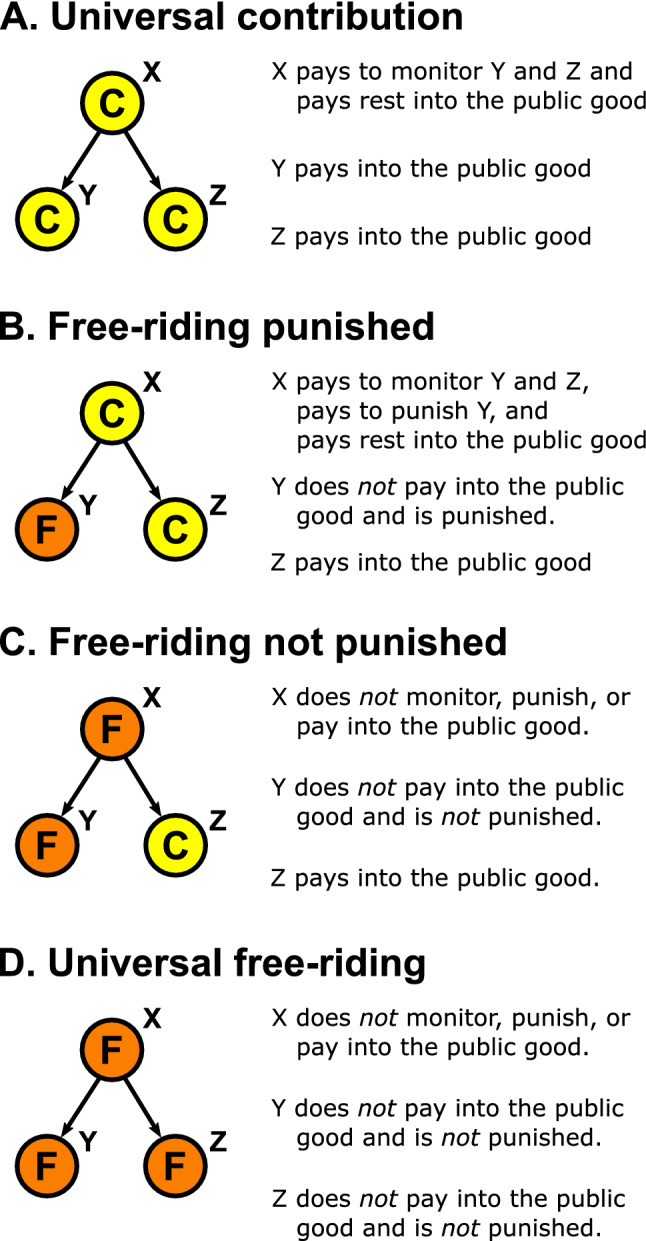
An example motif, with one individual responsible for monitoring and punishing the other two, having different mixes of contributors and free-riders. Nodes labeled (**C**) (yellow) represent contributors and nodes labeled (**F**) (orange) represent free-riders. In (**A**) contributing is universal. Individual X pays 2/*m* to monitor individuals Y and Z and contributes $c_t - 2/m$, to the public good. Individuals Y and Z each contribute $c_t$ to the public good. In (**B**) individual X pays 2/*m* to monitor individuals Y and Z, pays $c_p$ to punish individual Y, and contributes $c_t - 2/m - c_p$ to the public good. Individual Z contributes $c_t$ to the public good. Individual Y is punished by Individual X which costs Individual Y $p c_p$. In (**C**) Individual Z contributes $c_t$ to the public good. Individuals X and Y contribute nothing and receive no punishment. In (**D**) all individuals are free-riders who contribute nothing and do not receive any punishment.

An individual’s payoff is thus a function of their strategy and the strategies of the rest of the individuals in their network. To find optimal networks, I assume that individuals’ strategies are in a Nash equilibrium where no individual would be better off unilaterally changing their strategy. In Appendix S1 I show that individuals only have two possible strategies if they are in a Nash equilibrium. One strategy is to be a “threshold contributor,” who contributes exactly $c_t$ following their institutional role. The threshold contributor pays the minimal amount to avoid punishment. The other possible strategy is to be a “pure free-rider” who contributes nothing at all while accepting any punishment that they might receive for free-riding. For the remainder of the paper, all references to “contributors” assume that they are threshold contributors, and all references to “free-riders” assume they are pure free-riders. For illustration, Fig. 3 shows a network motif of three individuals with one responsible for monitoring and punishing the other two. The four different combinations of contributors and free-riders on the motif result in different payoffs for each individual.

I define “optimal networks” as those which have the highest social efficiency, or highest total group payoff, assuming individuals’ strategies are at a Nash equilibrium. While one network structure can have different mixtures of free-riding in Nash equilibrium, I show in Appendix S2 that for socially efficient structures at Nash equilibrium, either every individual is a contributor (i.e., universal threshold contribution) or every individual is a free-rider (i.e., universal pure free-riding). Therefore, for socially efficient networks and a socially efficient Nash equilibrium, the possible payoffs to an individual, $\pi$, depends on whether their network has universal threshold contribution or universal pure free-riding, as shown in Table 2.

**Table 2:**
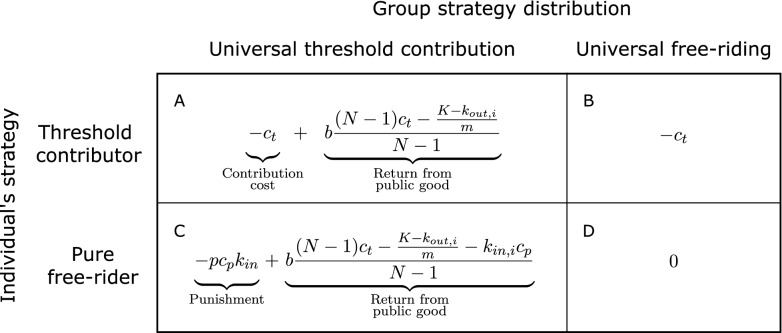
Payoffs for threshold cooperation or pure free-riding for an individual under conditions where every other individual in the group is a threshold contributor or a pure free-rider. Threshold cooperation and pure free-riding are the only two Nash strategies and universal threshold contribution and universal free-riding are the only two possible socially efficient distributions of strategies within a group.

## Analysis

Determining which organizational structures are optimal for different parameter values requires that we (1) determine the conditions under which networks can be found where universal threshold contribution is socially efficient and a Nash equilibrium, (2) determine which of these structures are socially optimal for different parameters, and (3) objectively measure how hierarchical the networks are under different conditions so that they can be compared.

Three conditions determine the parameters where universal contribution is both socially efficient and a Nash equilibrium. The first condition, as derived in Appendix S3, is that the benefits from the public good must be great enough to pay the monitoring costs implied by the network. Specifically, Eq. (1) gives the condition for universal threshold contribution to be more socially efficient than universal free-riding.

1 $$\begin{aligned} b > \frac{N c_t}{N c_t - K/m} \end{aligned}$$

The second condition, as derived in Appendix S4, is that the minimum in-degree for each node in the network, $\text {min}(k_{in,i})$, must be high enough for there to be enough punishment to incentivize threshold contributions from each individual. Specifically, Eq. (2) shows that the punishment efficiency sets each node’s minimum in-degree. Since punishment efficiency determines the number of punishers required to incentivize an individual to contribute, the higher the punishment efficiency, the lower the minimum in-degree.

2 $$\begin{aligned} \text {min}(k_{in,i}) = \Biggl \lceil \frac{c_t}{c_p \left( p + \frac{b}{N - 1}\right) } \Biggr \rceil \end{aligned}$$

An individual’s “span-of-control” describes how many others they can effectively monitor and is a classic topic in organizational economics^46^. The third condition, as derived in Appendix S5, determines each individual’s span-of-control and is defined as the maximum out-degree for each node in the network, $\text {max}(k_{out,i})$. Effectively monitoring more individuals takes more resources, so an individual’s span-of-control must be low enough that the individual can pay the monitoring cost under the contribution threshold. Specifically, Eq. (3) shows that the monitoring efficiency sets each node’s maximum out-degree. Since monitoring efficiency determines the number of others that one individual can effectively monitor, the higher the monitoring efficiency, the higher the maximum out-degree.

3 $$\begin{aligned} \text {max}(k_{out,i}) = \Bigl \lfloor m \left( c_t - c_p \right) \Bigr \rfloor \end{aligned}$$

If the condition in Eq. (1) is met, the two constraints in Eqs. (2) and (3) determine the possible network structures for the network. Each individual requires a minimum in-degree, but can only produce a maximum out-degree. Therefore, socially efficient contribution networks can only be constructed when $\text {max}(k_{out,i}) \ge \text {min}(k_{in,i})$. Furthermore, as shown in Appendix S6, the most efficient of these networks are the ones that minimize the number of links so that $K = N \text {min}(k_{in,i})$.

To objectively compare how distributed or hierarchical optimal network structures are for different parameter values, I use existing measures developed by Krackhardt^47^ for directed networks. These four measures, “graph hierarchy,” “graph efficiency,” “connectedness,” and “least-upper-boundness,” each range from zero to one. When all four measures of a network are equal to one, the network is the most hierarchical and satisfies necessary and sufficient conditions to be an out-tree. Larger values across all four measures indicate more hierarchical networks and smaller values indicate more distributed networks. Networks in this paper all have maximum connectedness and least-upper-boundness scores, so I will only use graph hierarchy and graph efficiency scores to measure these networks.

A graph hierarchy, *H*, score for a network of one component is calculated as one minus the fraction of reciprocally-linked pairs of nodes to linked nodes in a network (Eq. 4). For example, nodes *A* and *B* are linked when there is a path from *A* to *B* or a path from *B* to *A*. Nodes *A* and *B* are reciprocally-linked when there is a path from *A* to *B* and a path from *B* to *A*. Since an out-tree has no reciprocally-linked nodes, its *H* score is one. By contrast, since every node in a completely connected network is reciprocally linked, its *H* score is zero.

4 $$\begin{aligned} H = 1 - \frac{\text {Number of reciprocally linked nodes}}{\text {Number of linked nodes}} \end{aligned}$$

A graph efficiency, *E*, score is calculated as one minus the number of links greater than $N-1$. For single-component networks, the *E* score is calculated as in Eq. (5). Since an out-tree has $N-1$ links, an out-tree’s *E* score is equal to one. By contrast, a completely connected network has the highest possible number of links, so its *E* score is zero.

5 $$\begin{aligned} E = 1 - \frac{\text {Number of links greater than } N-1}{\text {Maximum possible links greater than } N-1} \end{aligned}$$

A composite hierarchy measure can be constructed by simply multiplying *E* and *H* together, resulting in a single value that ranges from 0 to 1 where higher numbers indicate more hierarchical networks. I use this measure to compare the relative hierarchy of networks where universal threshold contribution is socially optimal and a Nash equilibrium under different parameter values. Component *E* and *H* scores are reported in the appendix.

For different parameter values I calculate whether they meet the conditions of Eq. (1) and, if they do, find the constraints on in-degree and out-degree from Eqs. (2) and (3). Using those constraints, I choose socially optimal networks which are those where $K = N \text {min}(k_{in,i})$ and take the subset of those where the number of pure contributors is maximized as described in Appendix S7. Pure contributors are individuals who do not monitor or punish and we would expect networks with more pure contributors to do better if there were any benefit to specialization. I then calculate *E* scores and the lower bound for *H* scores from this subset of networks as described in Appendices S8 and S9. I then generated one million random samples of these networks for each $\text {min}(k_{in,i})$ and $\text {min}(k_{out,i})$ of interest for networks of size $N = 6$ and $N = 12$ and recorded the distribution of *H* scores for each sample. I also conducted a separate, targeted, search to find socially-optimal networks with higher maximum *H* scores than those found in the random sample.

## Results

**Figure 4:**
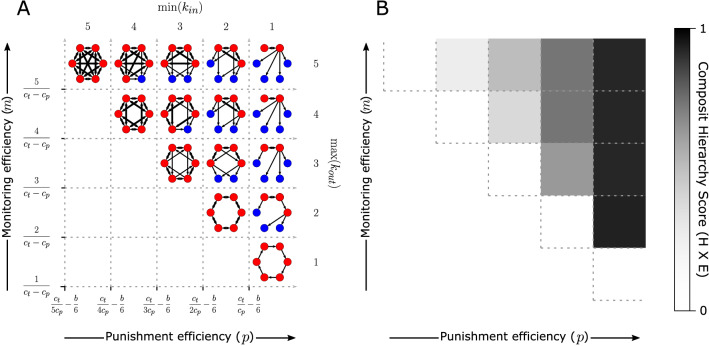
Six-node organizations where universal contribution is a socially efficient Nash Equilibrium are distributed or hierarchical depending on monitoring and punishment efficiencies. Punishment efficiency sets the minimum in-degree (Eq. 2) and monitoring efficiency sets the maximum out-degree (Eq. 3) for each node. In (**A**), directed links represent monitoring and punishment responsibilities and thicker links are reciprocal. Blue nodes are pure contributors with zero out-degree. Composite hierarchy scores for each organization are shown in (**B**) with higher values indicating more hierarchy. Less costly monitoring and punishment favor more hierarchical organizations. When punishment is sufficiently cheap, optimal organizations are tree-like with high composite hierarchy scores. When is cheap to monitor, but expensive to punish, the optimal structure is completely distributed.

For illustration, Fig. 4A shows socially-optimal networks for organizations of $N = 6$ individuals under different monitoring and punishment efficiencies. Figure 4B shows their corresponding composite hierarchy scores. These networks were determined by constraining the in-degree of each node to the minimum required by Eq. (2) and not allowing the out-degree of any node to exceed the maximum allowed by Eq. (2) for each set of parameters. The networks also maximize the number of pure contributors as described above. For all networks shown, universal contribution is the socially optimal Nash equilibrium for the parameter values. In areas where no networks are shown, universal free-riding is the socially optimal Nash equilibrium.

When monitoring efficiency is high and punishment efficiency is low, i.e., where $\text {min}(k_{in}) = 5$ and $\text {max}(k_{out}) = 5$, the socially optimal network is a massively distributed completely-connected graph. Monitoring is entirely universal and punishment is completely collective. This is similar to, for example, warfare in small-scale societies. Both the graph efficiency and graph hierarchy scores of this network are zero, as is the composite score where these are multiplied together.

With higher punishment efficiency, socially-optimal networks are more hierarchical. This is because the number of monitors, and thus the number of monitoring links, needed to incentivize contribution is lower. This, therefore, increases the graph efficiency score for optimal networks, which also increases the composite hierarchy score. As also shown in Fig. 4, higher punishment efficiency also allows for universal contribution when monitoring is less efficient.

When punishment efficiency is sufficiently high, each individual only needs one monitor to incentivize contribution and socially optimal networks resemble out-trees (with one reciprocal link, as shown on the far right of Fig. 4A. The composite hierarchy score for these tree-like networks is very close to one and monitoring efficiency determines each individual’s maximum span-of-control. Greater monitoring efficiency results in socially-optimal networks with a greater possible span-of-control and flatter hierarchical structures. As shown in Fig. 4, there is a special case, where punishment efficiency is high, but monitoring efficiency is low and the only efficient network is a cycle instead of a tree. A cyclic organizational structure seems impractical and may be particularly susceptible to external shocks, a modeling possibility discussed later in this paper.

While Fig. 4 shows optimal organizations and hierarchy scores for organizations of only six people, graph efficiency scores for optimal organizations can be calculated analytically for arbitrarily large networks, as derived in Appendix S9. There can be socially optimal network structures for the same parameter values that have different graph hierarchy, *H*, scores. The lower-bound of these *H* scores, $H^*$, can also be calculated analytically as shown in Appendix S9. This equation shows that the lower-bound *H* score depends on both monitoring and punishment efficiency, and does not account for higher *H* scores that are sometimes possible for different parameter values.

Properties of the networks shown in Fig. 4, including component *E* and *H* scores, are given in Appendix S10. When $\text {min}(k_{in}) = 1$ and Equation $\text {max}(k_{out}) = 2$ there is an alternative network structure with a different composite hierarchy score, as described in Appendix S11.

**Figure 5:**
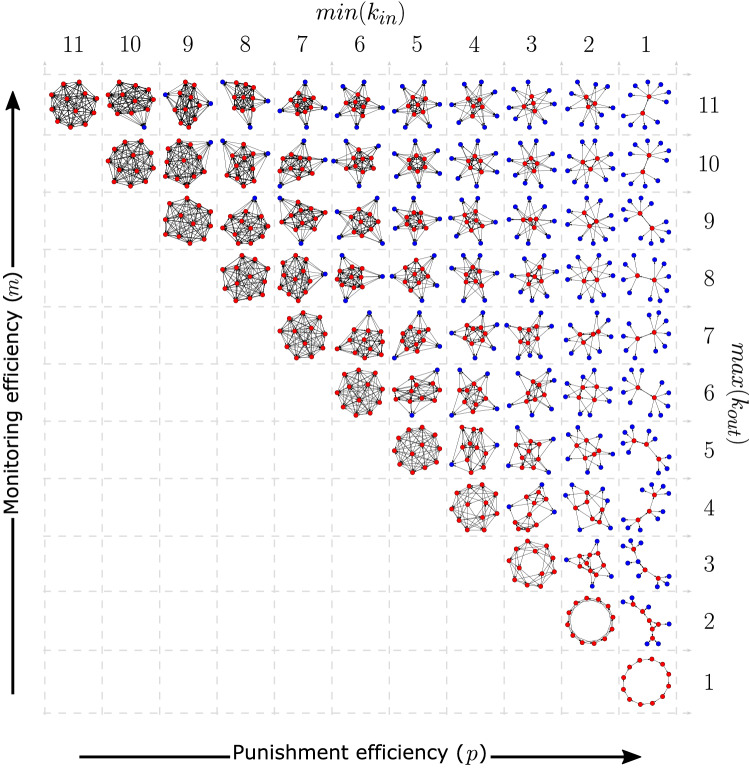
Socially efficient network structures for twelve individuals (nodes) when universal threshold contribution is a Nash equilibrium. The networks shown maximize the division of labor between those with monitoring and punishment responsibilities (red nodes) and those without (blue nodes) and, of those, have the median network hierarchy score from 1 million randomly generated networks. As with the six-node networks in Fig. 4, higher monitoring efficiency corresponds to a higher maximum number of out-links, and higher punishment efficiency corresponds to a lower minimum number of in-links. The optimal network structures range from completely connected, networks indicating distributed monitoring and punishment, at the top left and hierarchically organized monitoring and punishment on the far right.

**Figure 6:**
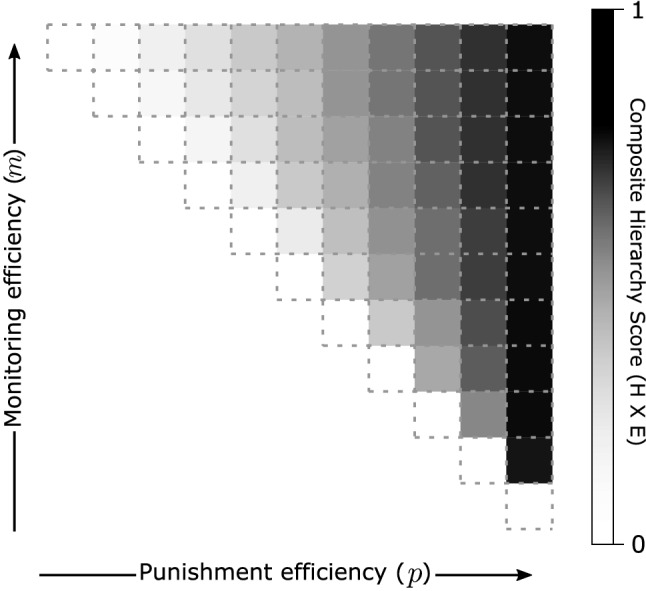
Composite hierarchy scores for the 12-node networks graphs in Fig. 5 show that, similar to the six-node networks in Fig. 4, greater monitoring and punishment efficiency corresponds with higher graph efficiency and graph hierarchy. Some parameter values had socially efficient graphs with greater composite scores. The graphs shown here are the median network hierarchy score from 1 million randomly generated networks. Versions of this plot with both component scores and with the lowest and highest hierarchy scores are in Appendix S12.

This relationship between monitoring and punishment efficiencies and organizational structure applies to larger organizations. Figures 5 and 6 show the results of a similar analysis for organizations of size $N = 12$. Figure 5 shows socially optimal organizational structures for different monitoring and punishment efficiencies that maximize pure contributors. Sometimes there can be multiple optimal networks for the same parameters and these networks sometimes can have different hierarchy scores. The networks in Fig. 5 are those with the median hierarchy score from a million randomly-generated optimal samples. Networks with minimum and maximum hierarchy scores are in Appendix S12.

As with six-node networks, when punishment gets less expensive, optimal networks are more hierarchical, and universal contribution is possible for lower monitoring efficiencies. When monitoring efficiency is high, but punishment efficiency is low, the optimal network structure has universal monitoring and collective punishment. Hierarchical out-trees are possible when punishment efficiency is sufficiently high with the maximum span-of-control determined by the monitoring efficiency. With twelve nodes, there are many more ways to organize these hierarchical networks. Properties of all possible 12-node optimal networks when $\text {min}(k_{in}) = 1$ are shown in Appendix S13.

Appendix S12 shows distributions of hierarchy scores of the randomly generated optimal networks for each parameter combination; detailed representations of networks with low, median, and high hierarchy scores networks for each parameter combination, and versions of Fig. 6 for networks with low, high, and median component and composite hierarchy scores.

These results apply to even larger networks. For example, similar results were found for 120-node networks as shown in Appendix S14.

## Discussion

This model demonstrates how hierarchical or distributed organizations are favored depending on the efficiencies of the institutions underlying cooperation. Specifically, the game-theoretic parameters for monitoring and punishment map onto existing network theoretic measures of hierarchical networks. Graph efficiency increases with punishment efficiency in a predictable way. Graph hierarchy increases with both monitoring and punishment efficiency. When monitoring and punishment are both costly, collective action is not socially efficient. When monitoring is efficient, but punishment is costly, command-and-control institutions are distributed with collective monitoring and punishment, resembling non-hierarchical societies and organizations^21,22,24,26,27^. With more efficient punishment, institutions are more hierarchical, with a command-and-control structure more resembling the out-tree of modern organizational charts with the monitoring efficiency constraining the maximum span-of-control.

These findings suggest a path by which cooperative institutions increased in scale and complexity in humanity’s history. First, as Eq. (1) suggests, there needs to be a public good with sufficient return on investment to pay for the institutional costs of monitoring others and punishing non-contributors. This is consistent with coarser-scale models of leadership and collective action (e.g.,^6^) which find that public goods are needed to pay for hierarchical institutions. Communal agriculture, fishing, or warfare were likely the earliest public good opportunities for larger groups. Although the model suggests that cooperative groups could grow indefinitely when completely hierarchical, group size would be limited if the return to public goods investments was non-linear, with decreasing returns to the public good with larger cooperative groups, as implied by Eq. (1).

Second, greater human cognitive capacity may have improved our ability to monitor and track the contributions of increasingly more community members^48,49^. This would allow for small-scale cooperative organizations with distributed monitoring and collective punishment, like those for high monitoring efficiency, but low punishment efficiencies in Figs. 4 and 5.

Third, greater human technological capacity may have increased the effectiveness of punishment^3^. Technological and institutional innovations, including stand-off weapons like spears and projectile weapons, have been hypothesized to lower the cost of punishing others and increase the ability to replace physically dominant despotic individuals with more egalitarian cooperative institutions^3^. As in the model above, the increase in punishment efficiency would also foster cooperative institutions to become more hierarchical and less distributed.

These findings also suggest that the scale of organizational types can be limited by the costs of monitoring and punishment. Turkana military organization is distributed and orders of magnitude smaller than many modern industrialized militaries. Distributed structures, unlike hierarchical structures, do not scale as the organization grows. However, large organizations, like Wikipedia, can maintain a distributed organization because the monitoring costs are incredibly cheap and punishment (banning an account for bad behavior) requires consensus of the interested community members.

An assumption in this paper is that socially efficient organizations would be favored over socially-inefficient ones. There are at least a couple of mechanisms that bring this about. For example, socially efficient institutions could out-compete and replace inefficient ones through economic competition, military competition, or between-group learning^25,50,51^ with network structures and institutions representing a type of “group-level trait”^52,53^. Similarly, individuals might choose, rationally design, or innovate efficient organizations from first principles without the need for competition or learning^9,54^. Or socially efficient organizations could be favored by a combination of these mechanisms.

This model describes socially efficient networks at Nash equilibria and provides a framework to explore the dynamics of organizational formation and maintenance. For example, the networks shown in Figs. 4 and 5 are more efficient than alternatives, in part, because they minimize monitoring and punishment within the parameter constraints. However, the most efficient organizations may not be as resistant to shocks as organizations with more redundancy^18^. Like many systems, there are likely trade-offs between efficiency and robustness that could be explored by subjecting these organizations to various types of shocks. Socially optimal networks might be especially susceptible to invasion by second-order free-riders who contribute to the public good but do not punish free-riders^34^ with further invasion by pure free-riders.

There might also be trade-offs between organizations optimized for fostering collective action and those optimized for collective information processing and decision-making^55^. In the model, hierarchical organizations are efficient for fostering collective action when punishment is inexpensive. However tree-like structures are often thought to be inefficient for information processing because hierarchy creates information bottlenecks or “stove-pipes”^56–59^. Hierarchical organizations may arise to solve cooperation problems but later graft information processing functions, inefficiently, onto the same hierarchical structure. These inefficient informational stove-pipes may be maintained because organizational managers use their coercive authority to control the flow of information through the organization^19^. The trade-offs between collective action and information processing in organizations could be formally explored with additional modeling.

Additionally, the model could help explore the differences between egalitarian and inegalitarian hierarchical societies. The organizations in this model are “egalitarian” in the sense that the contribution threshold is the same for all individuals and all contributors have the same payoff. The relationship between hierarchical organizations and inequality is complicated. While societies with distributed organizations can have inequality^60^, hierarchical organizations themselves might cause and help maintain inequalities in power and wealth, with those at the top of the hierarchy having more of both which can then be used to maintain their control^2,61^. While some early hierarchical political institutions, simple chiefdoms, have been hypothesized to have been relatively egalitarian with chiefs coordinating collective action but not accumulating wealth or power above the typical member of the group^3,62,63^, it is possible that once hierarchical organizations are established, individuals with coercive power may use it to gain additional resources and maintain their position. For example, egalitarian simple chiefdoms sometimes transition into non-egalitarian “complex” chiefdoms where chiefs use their position to gain wealth and power and even pass their wealth, power, and status to their descendants^7,62^. This suggests that individuals with more out-degree centrality (i.e., those with greater out-degree) or Katz centrality^64^ (i.e., those higher-up in a hierarchical directed network) would have higher payoffs than other individuals. The payoff structure of the network model in this paper could be extended to capture the conditions where egalitarianism is stable and conditions that favor transitions to social inequality.

Finally, an anonymous reviewer suggested that the model in this model may have implications for global collective action problems, such as climate change mitigation. Solving global collective action problems, and especially greenhouse gas reduction, is a classic problem of institutional design^65,66^. Effective climate change agreements that are negotiated between countries have a free-rider problem as there is no overarching centralized institution that can punish non-cooperators^67^. Several authors have proposed that organizing countries into smaller independent climate agreements could lead to global collective action^68–72^. However, these proposals do not specify how the second-order free-rider enforcement problem can be overcome within these smaller agreements^73^. The model in this paper suggests that, depending on monitoring and enforcement costs, there may be efficient network structures that, depending on monitoring and punishment costs, can maintain global cooperation with either distributed or hierarchical enforcement. Many international agreements have decentralized enforcement mechanisms. For example, voluntary agreements like the World Trade Organization are designed with a decentralized punishment scheme for those who do not comply with the agreements^9^. However, it is unclear how these networks would form. More efficient networks are unlikely to emerge through competition with other networks because the benefits of carbon emission reduction by one network would accrue to all competing networks^66,73^. Some sort of “rational design”^9^ process would likely be required with all or most nations as members of the organization^11^. Also, it is to be determined how well these networks would respond to shocks, such as a member nation exiting the network or refusing to enforce sanctions on free-riders. A fully dynamic model of network formation and response to shocks, that included heterogeneity between member nations, would help determine the feasibility of various network structures for fostering global cooperation.

## Supplementary Information

Supplementary Information.

## Data Availability

The datasets generated and analysed during the current study are available in the OSF repository at https://osf.io/hksf4/?view_only=62e607346f7d47ffaa1d6db8312bc20d.
