# Supplementary material for: Constraints on cooperation shape hierarchical versus distributed structure in human groups
Source: Sci Rep. 2023 Jan 20;13:1160. doi: 10.1038/s41598-022-23454-9 (PMC9860035; doi:10.1038/s41598-022-23454-9)
Supplement: Supplementary file 1 — Supplementary Information. [file 41598_2022_23454_MOESM1_ESM.pdf]

# Supplementary materials

## S1 Nash strategies are pure free-riding or threshold contribution

Consider a generalized payoff model for an individual in a public goods game with punishment. To avoid being punished at a cost of $P \geq 0$ the individual must pay at least a threshold amount of $c_t \geq 0$. They receive a baseline public goods benefit of $G \geq 0$. However, if they are punished, an amount $0 \leq g \leq G$ is subtracted from this benefit due to whatever resources go towards punishing the individual instead of towards the public good.

A threshold contributor pays exactly $c_t$ and has a payoff as show in Equation S1.

$$\pi_{tc} = -c_t + G \tag{S1}$$

A pure free-rider pays zero and has a payoff as shown in Equation S2.

$$\pi_{pf} = -P + G - g \tag{S2}$$

Consider a strategy called "fractional free-rider" that pays a cost, $c_f$ less than the threshold so that $0 < c_f < c_t$. This strategy still receives punishment and has a payoff as shown in Equation S3.

$$\pi_{ff} = -c_f - P + G - g \tag{S3}$$

Comparing Equation S2 to Equation S3, the pure free-riding strategy dominates the fractional free-riding strategy because the payoff is the same except that the pure free-rider does not pay $c_f$.

Consider a strategy call "super-contributor" that pays a cost, $c_s$ greater than the threshold so that $c_s > c_t$. This strategy avoids punishment and has a payoff as shown in Equation S4.

$$\pi_{sc} = -c_s + G \tag{S4}$$

Comparing Equations S1 to S4, the threshold contribution strategy dominates the super-contributor strategy because $c_s > c_t$ by definition.

Therefore threshold contribution and pure free-riding are the only two possible Nash strategies because one of them dominates any other contribution strategy. Contributing more than $c_t$ is not a Nash strategy because it is costly but has no additional benefit for the individual because the individual only needs to contribute $c_t$ to avoid punishment. Contributing more than zero and less than $c_t$ is also

not a Nash strategy because it is more costly than contributing zero and the individual can still be punished.

# S2 Pure-strategy dominate mixed-strategy networks

In Appendix S1 I showed that the only strategies at a Nash equilibrium are threshold contribution or pure free-riding. In this section, I will show that when individuals' strategies are at a Nash equilibrium, the most socially efficient networks are those that support universal threshold contribution as long as they are more socially efficient than universal free-riding.

Equation S5 gives the social efficiency of a network with universal contribution, which is just the sum of the payoffs to all individuals in the network, which can be found by summing the payoffs over all individuals in a universal contribution network with each individual having the payoff in Table 2A.

$$\Pi_C = \sum_i \pi_{C,i} = \underbrace{-Nc_t}_{\substack{\text{Total}\\ \text{contribution}\\ \text{cost}}} \underbrace{+b\left(Nc_t - \frac{K}{m}\right)}_{\substack{\text{Total return}\\ \text{from public good}}} \tag{S5}$$

Equation S6 gives the social efficiency for a network with universal free-riding. The network structure of this network does not matter to the payoff since no individual is following their institutional role.

$$\Pi_F = \sum_i \pi_{F,i} = 0 \tag{S6}$$

Consider a network that is intermediate between universal threshold contribution and universal free-riding. Specifically, there are $n_c < N$ threshold contributors and $N - n_c$ pure free-riders. I define $K_c$ as the total number of out-links from threshold contributors in the network and $n_{kf} \leq n_c$ as the total number of contributors with at least one out-link to a free-rider.

The total payoff to the pure free-riders in the network is $\Pi_f$, as shown in Equation S7. This is just the fraction of the public good going to the free-riders.

$$\Pi_f = b\frac{N - n_c}{N}\left(n_c c_t - \frac{K_c}{m} - n_{kf}c_p\right) - pn_{kf}c_p \tag{S7}$$

The total payoff to the threshold contributors in the network is $\Pi_c$, as shown in Equation S8.

$$\Pi_c = -n_c c_t + \frac{n_c}{N}b\left(n_c c_t - \frac{K_c}{m} - n_{kf}c_p\right) \tag{S8}$$

The social efficiency of the mixed network is the total payoff, $\Pi_M$, the sum of $\Pi_c$ and $\Pi_f$, as shown in Equation S9.

$$\Pi_M = \Pi_f + \Pi_c = -n_c c_t + b\left(n_c c_t - \frac{K_c}{m} - n_{kf} c_p\right) - p(N - n_c) n_{kf} c_p \tag{S9}$$

Equation S9 simplifies to Equation S10.

$$\Pi_M = -n_c c_t + b\left(n_c c_t - \frac{K_c}{m}\right) - \left(b + p(N - n_c)\right) n_{kf} c_p \tag{S10}$$

For the mixed network to be more socially efficient than both the universal threshold contribution network and the universal pure free-riding network, both $\Pi_M > \Pi_C$ and $\Pi_M > \Pi_F$ must be true. To simplify the notation, I will define $P_M$ as the total punishment cost in the mixed network so that $P_M = \left(b + p(N - n_c)\right) n_{kf} c_p$ which, since $N > n_c$, is always positive. Also, the total number of links in the most socially efficient universal threshold contributor network is the total number of nodes times the minimum in-degree for each node, $K = Nk_{min}$. The total number of links needed to incentivize contribution in the mixed network is the total number of contributing nodes times the minimum in-degree for each node, $K_c = n_c k_{min}$. The new payoff for the mixed network is in Equation S11.

$$\Pi_M = -n_c c_t + b\left(n_c c_t - \frac{n_c k_{min}}{m}\right) - P_M = n_c\left(bc_t - c_t - \frac{k_{min}}{m}\right) - P_M \tag{S11}$$

First, solving for the conditions when the payoff to the mixed network is greater than for the universal pure free-riding network, $\Pi_M > \Pi_F$. Using Equations S6 and S11 results in Equation S12.

$$n_c\left(bc_t - c_t - \frac{k_{min}}{m}\right) - P_M > 0 \tag{S12}$$

Equation S12 implies Equation S13. This is true for any value of $P_M \geq 0$ and any value of $n_c > 0$.

$$bc_t - c_t - \frac{k_{min}}{m} > 0 \tag{S13}$$

Next, solving for the conditions when the payoff to the mixed network is greater than for the universal threshold contribution network, $\Pi_M > \Pi_C$. Using Equations S5 and S11 results in Equation S14.

$$-n_c c_t + b\left(n_c c_t - \frac{n_c k_{min}}{m}\right) - P_M > -Nc_t + b\left(Nc_t - \frac{Nk_{min}}{m}\right) \tag{S14}$$

Rearranging Equation S14 results in Equation S15.

$$n_c\left(bc_t - c_t - \frac{k_{min}}{m}\right) - P_M > N\left(bc_t - c_t - \frac{k_{min}}{m}\right) \tag{S15}$$

Since $bc_t - c_t - \frac{k_{min}}{m}$ must be positive according to Equation S13 and $N > n_c$ by definition, the conditions in Equation S13 and S15 can never by simultaneously met - even if $P_M = 0$ so that there is no punishment in the mixed network. Therefore, a mixed network at a Nash equilibrium cannot be more socially efficient than both a universal pure free-riding network at equilibrium and a universal threshold contribution network at equilibrium.

# S3 Conditions for socially efficient contribution networks

In Appendix S1 I showed that socially efficient networks are either entirely free-riders or entirely threshold contributors. For free-riding networks, the network structure is irrelevant since no individuals are monitoring or punishing.

Finding the conditions where the social efficiency of universal contribution is greater than the social efficiency of universal free-riding, $\Pi_C > \Pi_F$, results in Equation S16 from Equations S5 and S6.

$$-Nc_t + b\left(Nc_t - \frac{K}{m}\right) > 0 \tag{S16}$$

Solving for $b$ results in Equation 1 in the main text, which gives the condition where a network of contributors can be more socially efficient than networks of free-riders. The public goods efficiency must be sufficiently high for returns from the public good to pay the cost of monitoring.

# S4 Punishment efficiency determines minimum in-degree

What is required to incentivize threshold contribution in an individual assuming that the condition in Equation 1 is met? From Appendix S1 we know that the only possible individual strategies at a Nash equilibrium are threshold contribution or pure free-riding. From Appendix S2 we know that the only possible socially efficient networks at Nash equilibrium either have universal threshold contribution or universal free-riding. Therefore the payoffs for an individual in such a socially efficient network are limited to those in Table 2 in the main text.

If the network has universal free-riding, a pure free-rider does not have an incentive to switch to threshold contribution. Comparing the payoff in Table 1B to that in Table 1D, it is always better to be a free-rider in a network of other free-riders because there are no other contributors to allocate punishment. In this case, the network structure does not matter since no individuals are monitoring or punishing anyway.

However, the more interesting case is on a network with universal contribution at the socially efficient Nash equilibrium. The condition where individual $i$'s is better off being a threshold contributor than a free-rider can be found by determining where the payoffs in Table 1A is higher than Table 1C, as shown in Equation S17

$$-c_t + b\frac{(N-1)c_t - K - \frac{k_{out,i}}{m}}{N-1} > -pc_p k_{in,i} + b\frac{(N-1)c_t - K - \frac{k_{out,i}}{m} - k_{in,i}c_p}{N-1} \tag{S17}$$

Solving for the minimum integer $k_{in,i}$ that satisfies this equation results in Equation 2 in the main text. This is the minimum in-degree required to incentivize individual $i$ to contribute. Especially when the network is large (i.e., $N >> b$), the minimum in-degree required to incentivize contribution depends on the punishment efficiency. This is because the greater the punishment efficiency, the fewer punishers are needed to incentivize individual $i$ to contribute.

# S5 Monitoring efficiency determines maximum out-degree

Since, as shown in Appendix S1, individuals will not contribute more than $c_t$ at a Nash equilibrium this limits how many other individuals they will monitor. Since contributors with monitoring responsibilities are expected to retain a punishment reserve of $c_p$ and are not expected to contribute more than $c_t$, the resources they have available for monitoring are, at maximum, $c_t - c_p$. Since the monitoring efficiency, $1/m$, is defined as the cost to effectively monitor each other individual, Equation 3 in the main text gives the maximum out-degree an individual can use to effectively monitor other individuals.

# S6 Optimal networks minimize links

Equation S5 implies that the social efficiency of networks with universal threshold contribution at a Nash equilibrium is also higher when $K$, the number of links in a network, is minimized. That is because it is more efficient to minimize monitoring costs, as long as threshold contribution is still a Nash strategy for each individual. Since, as shown in Appendix S4, each individual in a network with universal contribution is required to have the minimum in-degree in Equation 2 to incentivize contributions, the minimum number of links in a socially efficient threshold contribution network is given by Equation S18. Each of the $N$ individuals in the network has the minimum number of links satisfying Equation 2, and no more.

$$K_{min} = N \left\lceil \frac{c_t}{c_p \left(p + \frac{b}{N-1}\right)} \right\rceil \tag{S18}$$

$K_{min}$ can also be substituted for $K$ Equation S5 to determine the maximum payoff for networks where universal contribution is socially efficient and a Nash equilibrium.

Similarly, the maximum number of links that can be generated by a network is given by multiplying the maximum out-degree for individuals in the network, from Equation 3, by the number of nodes in the network, as shown in Equation S19.

$$K_{max} = N \left\lfloor m \left(c_t - c_p\right) \right\rfloor \tag{S19}$$

Since $K_{max}$ must be as least as large as $K_{min}$ for universal threshold contribution to be socially efficient and a Nash Equilibrium, the conditions where this occurs, as given in Equation S20, define the conditions where it is possible to have a socially efficient network with universal threshold contribution.

$$\left\lceil \frac{c_t}{c_p(p + \frac{b}{N-1})} \right\rceil \leq \left\lfloor m(c_t - c_p) \right\rfloor \tag{S20}$$

## S7 Maximum number of pure contributors

In this appendix, I derive the maximum number of pure contributors (those that do not have monitoring or punishing responsibilities in a socially efficient network). Networks with more pure contributors would potentially result if there were marginal benefits to managerial specialization.

First, a network where universal threshold contribution is a Nash equilibrium must have a minimum number of links, as described in Equation S18. Additionally, the maximum number of links that each individual can produce is described in Equation 3 in the main text. From these we can calculate the minimum number of core individuals, $C$, it takes to produce sufficient monitoring links for the network, as shown in Equation S21.

$$C = \left\lceil \frac{K_{min}}{\max(k_{out})} \right\rceil = \left\lceil \frac{K_{min}}{\lfloor m\,(c_t - c_p) \rfloor} \right\rceil \tag{S21}$$

The maximum number of pure contributors, $P$ in the network are the remaining individuals, as shown in Equation S22.

$$P = N - C = N - \left\lceil \frac{K_{min}}{\lfloor m\,(c_t - c_p) \rfloor} \right\rceil \tag{S22}$$

# S8 Analytic calculation of graph efficiency

In this appendix, I show how to analytically calculate the graph efficiency of networks where universal threshold contribution is socially efficient and a Nash equilibrium and the number of pure contributors are maximized.

Starting with Krackhardt's definition of graph efficiency for single-component networks as shown in Equation 5, I will first derive the numerator for the right side of Equation 5, which is the number of links in the network greater than $N-1$. Equation S18 shows $K_{min}$, the minimum number of links needed for contribution in a socially efficient network where universal threshold contribution is a Nash Equilibrium. Therefore, the number of required links in one of these networks greater than $N-1$ is:

$$\text{Number of links greater than } N-1 = K_{min} - (N-1) = N\left\lceil \frac{c_t}{c_p\left(p+\frac{b}{N-1}\right)} \right\rceil - N + 1$$

Next, I will derive the denominator, which is the maximum possible number of links greater than $N-1$. Since the maximum number of possible links in the network is $N(N-1)$, the maximum possible links greater than $N-1$ is $N(N-1) - N + 1$. Simplified, this becomes:

$$\text{Maximum possible links greater than } N-1 = (N-1)^2$$

Therefore, an analytic calculation of graph efficiency for a network where threshold contribution is both a Nash equilibrium and socially efficient can be calculated as shown in Equation S23.

$$E = 1 - \frac{N\left\lceil \frac{c_t}{c_p\left(p+\frac{b}{N-1}\right)} \right\rceil - N + 1}{(N-1)^2} \tag{S23}$$

This efficiency calculation corresponds to the graph efficiencies found in all of the randomly generated networks reported in Appendices S10 and S12. Note that graph efficiency only depends on $K_{min}$ which is also consistent with the tables in the appendices where all networks with the same $K_{min}$ have the same efficiency. Note also that efficiency always increases with $K_{min}$ which is consistent with the results of the paper.

# S9 Analytic calculation of hierarchy lower-bound

Equation 5 in the main text, defines of Krackhardt's graph hierarchy for a single-component graph.

A lower bound for the graph hierarchy of socially efficient networks where universal threshold contribution is a Nash equilibrium and pure contributors are maximized can be calculated starting with Equation S21 which defines a core number of nodes, $C$, who are not pure contributors.

While, for some parameter combinations, networks with higher hierarchy scores are possible (see Appendices S10, S11, and S12, and S13), the lower-bound value of $H$ can be calculated assuming that all core nodes are in each other's path (this maximizes the number of mutually reachable links in the core). Therefore, the number of pairs of reciprocally linked nodes for lower-bound calculations of graph hierarchy is $\frac{C(C-1)}{2}$.

Since, for the lower-bound calculation, all core nodes are in each other's path, but none of the non-core nodes are in each other's path, the number of linked nodes in the network can be calculated as the number of linked nodes that are possible in the network, $\frac{N(N-1)}{2}$, minus the number of links that would be possible between the pure contributors, $\frac{(N-C)(N-C-1)}{2}$.

Substituting these values into Equation 5, gives the lower-bound hierarchy score, $H_L$, for the network as shown in Equation S23.

$$H_L = 1 - \frac{\frac{C(C-1)}{2}}{\frac{N(N-1)}{2} - \frac{(N-C)(N-C-1)}{2}} = 1 - \frac{C-1}{2N-C-1} = \frac{2N-2C}{2N-C-1} \tag{S24}$$

Making substitutions from Equations 2, 3, S21, and S18 in the main text, results in Equation S25.

$$H_L = \frac{2N - 2\left\lceil \frac{K_{min}}{\max(k_{out})} \right\rceil}{2N - \left\lceil \frac{K_{min}}{\max(k_{out})} \right\rceil - 1} = \frac{2N - 2\left\lceil \frac{\frac{Nc_t}{c_p\left(p+\frac{b}{N-1}\right)}}{\lfloor m\left(c_t - c_p\right)\rfloor} \right\rceil}{2N - \left\lceil \frac{\frac{Nc_t}{c_p\left(p+\frac{b}{N-1}\right)}}{\lfloor m\left(c_t - c_p\right)\rfloor} \right\rceil - 1} \tag{S25}$$

Equation S25 simplifies to Equation Equation S26.

$$H_L = \frac{2N - 2\left\lceil \frac{Nc_t}{c_p\left(p+\frac{b}{N-1}\right)\lfloor m\left(c_t - c_p\right)\rfloor} \right\rceil}{2N - \left\lceil \frac{Nc_t}{c_p\left(p+\frac{b}{N-1}\right)\lfloor m\left(c_t - c_p\right)\rfloor} \right\rceil - 1} \tag{S26}$$

This calculates the lower-bound graph hierarchy score for a socially efficient network where threshold contribution is a Nash equilibrium and pure contributors are maximized. These analytic calculations match the lower-bound hierarchy scores from all sets of randomly-generated networks in Appendices S10, S11, S12, and S13, and these scores are often the median score from the randomly generated graphs. However, it is sometimes possible to find networks with much hierarchy hierarchy scores for the same payoff, as shown in Appendices S10, S11, S12, and S13. In the appendices, I also report the highest hierarchy scores found through random and targeted searches.

# S10 Six-node network properties

Table S1 shows the properties socially efficient contribution networks of six nodes with pure contributors maximized for each value of min$(k_{in})$ and max$(k_{out})$ as determined by Equations 2 and 3, respectively. The networks themselves are shown in Figure 4A in the main text.

The graph efficiency score for each condition was calculated analytically as described in Appendix S8 and also measured from each generated graph in R using the “efficiency” function from the *sna* package (version 2.6) in R. The analytic calculation matched the measured value in all cases. Note that the graph efficiency score is lower when Equation 2 is higher and always has the same value for the same Equation 2. This is consistent with the analytical findings.

The graph hierarchy ($H$) score was also measured for each generated network using the “hierarchy” function from the *sna* package (version 2.6) in R. The low scores match those calculated in Appendix S9. Only one network had an alternate network configuration with a different, higher, $H$ score. This is where Equation 2 $= 1$ and Equation 3 $= 2$. The alternate network is shown in Appendix S11.

The “$E \times H$” column multiplies the graph efficiency ($E$) score by the graph hierarchy ($H$) score to give the composite score shown in Figure 4B in the main text.

Figure S1 shows a plot of how these scores change for different values of punishment efficiency and monitoring efficiency.These plots correspond to the boxes in Figure 4. It is similar to the plot in Figure 4B, except that it shows both components ($E$ and $H$) of the composite score.

| $\min(k_{in})$ | $\max(k_{out})$ | Graph efficiency | Graph hierarchy (low) | Graph hierarchy (high) | $E \times H$ (low) | $E \times H$ (high) |
|---|---|---|---|---|---|---|
| 1 | 1 | 0.96 | 0.00 | 0.00 | 0.00 | 0.00 |
| 1 | 2 | 0.96 | **0.75** | **0.91** | 0.72 | 0.87 |
| 1 | 3 | 0.96 | 0.89 | 0.89 | 0.85 | 0.85 |
| 1 | 4 | 0.96 | 0.89 | 0.89 | 0.85 | 0.85 |
| 1 | 5 | 0.96 | 0.89 | 0.89 | 0.85 | 0.85 |
| 2 | 2 | 0.72 | 0.00 | 0.00 | 0.00 | 0.00 |
| 2 | 3 | 0.72 | 0.57 | 0.57 | 0.41 | 0.41 |
| 2 | 4 | 0.72 | 0.75 | 0.75 | 0.54 | 0.54 |
| 2 | 5 | 0.72 | 0.75 | 0.75 | 0.54 | 0.54 |
| 3 | 3 | 0.48 | 0.00 | 0.00 | 0.00 | 0.00 |
| 3 | 4 | 0.48 | 0.33 | 0.33 | 0.16 | 0.16 |
| 3 | 5 | 0.48 | 0.57 | 0.57 | 0.27 | 0.27 |
| 4 | 4 | 0.24 | 0.00 | 0.00 | 0.00 | 0.00 |
| 4 | 5 | 0.24 | 0.33 | 0.33 | 0.08 | 0.08 |
| 5 | 5 | 0.00 | 0.00 | 0.00 | 0.00 | 0.00 |

Table S1: Network properties for six-node socially efficient networks where universal threshold contribution is a Nash equilibrium and pure contributors are maximized. The first two columns give the minimum links needed to incentivize threshold contributions and the maximum number of out-links per node that can be paid for with a threshold contribution. Graph hierarchy minimums and maximums were determined from an exhaustive search of possible networks with the lower bound also calculated analytically as described in Appendix S9. The case where there are differences in hierarchy scores are shown in Appendix S11. Graph efficiency was calculated analytically, as described in Appendices S8, and validated with an exhaustive search. The calculated and simulated values agree. Bold numbers indicate where there is a difference between the high and low graph hierarchy values calculated for simulated network structures.

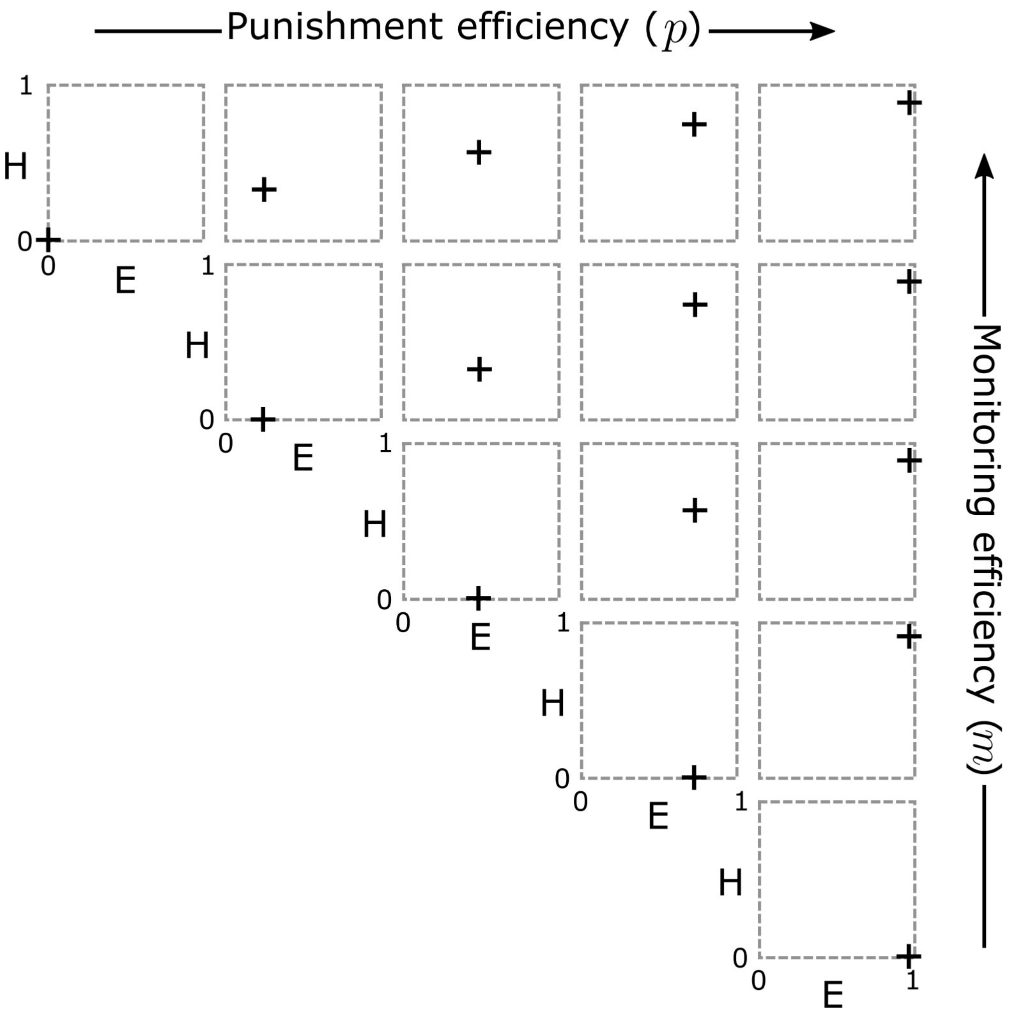


Figure S1: Graph efficiency (x-axes) and graph hierarchy (y-axes) scores for the networks in Figure 4 show that networks become more hierarchical and efficient as monitoring and punishment efficiency increase. As both of these measures approach one, the network approaches a hierarchical out-tree. Consistent with qualitative analysis of the network structures in Figure 4 and Table S1, graph efficiency and hierarchy are low with high monitoring efficiency and low punishment efficiency, indicating a distributed network. When monitoring efficiency is higher, graph hierarchy is higher. When punishment efficiency is higher the graph efficiency is higher. The most graph hierarchical and graph efficient networks occur with high punishment efficiency.

## S11 Alternate six-node network

This is the alternate network structure for a socially efficient contribution network with six pure contributors where $\min(k_{in}) = 1$ and $\max(k_{out}) = 2$. It has a lower graph hierarchy score than the network in Figure 4 because of three-node cycle, instead of the two-node cycle, at its base.

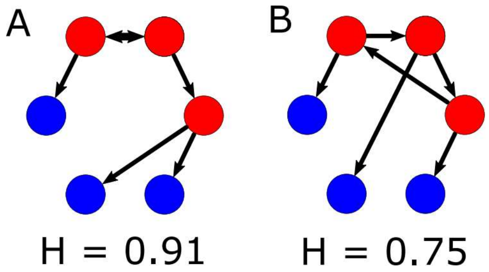


Figure S2: Alternate versions of a socially efficient six-node network where universal threshold contribution is a Nash equilibrium and $\min(k_{in}) = 1$ and $\max(k_{out}) = 2$. These networks have different graph hierarchy scores as shown in Table S1

# S12 Twelve-node network properties

Table S12 shows the properties socially efficient contribution networks of twelve nodes with pure contributors maximized for each value of min$(k_{in})$ and max$(k_{out})$ as determined by Equations 2 and 3, respectively. The networks themselves are shown in Figure 5 in the main text.

The graph efficiency score for each condition was calculated analytically as described in Appendix S8 and also measured from each generated graph in R using the "efficiency" function from the *sna* package (version 2.6) in R. The analytic calculation matched the measured value in all cases. Note that the graph efficiency score is lower when min$(k_{in})$ is higher and always has the same value for the same min$(k_{in})$. This is consistent with the analytical findings.

The graph hierarchy $(H)$ score was also measured for each generated network using the "hierarchy" function from the *sna* package (version 2.6) in R. The "low" scores match those calculated in Appendix S9.

The find the "median" and "high" $H$ scores, I generated a random sample of one million networks socially efficient contribution networks of twelve nodes with pure contributors maximized for each combination of min$(k_{in})$ and max$(k_{out})$ and measured their hierarchy scores. For some parameter combinations, the algorithm did not generate the highest possible $H$ scores, even with one million random samples. To find these more hierarchical networks I conducted a more targeted random search of networks with properties that were likelier to have more paths and few cycles. High $H$ scores are marked with an asterisk if the targeted search found a higher value than the random search. The distributions of the hierarchy scores for these samples are in Tables S3-S68 along with examples of networks with low, median, and high H scores for each combination of min$(k_{in})$ and max$(k_{out})$.

The networks were generated and analyzed in R (version 4.0.3) using the *igraph* package (version 1.2.6) and the *sna* package (version 2.6). Networks were constructed for each combination of minimum in-degree and maximum out-degree where min$(k_{in}) \leq$ max$(k_{out}) < N$. The R code for these procedures is in the OSF repository located at this URL: https://osf.io/hksf4/?view_only=62e607346f7d47ffaa1d6db8312bc20d.

First, the algorithm calculates the maximum possible number of pure contributors using the equation in Appendix S7. Then it generates a connectivity matrix with this number of nodes having no out-degree. Then it calculates the minimum number of links needed to incentivize cooperation as determined by Equation S18, and distributed these links randomly in the rest of the network. Then checked to see if any nodes had an out-degree greater than what is allowed by Equation 3 and if so,

it picked one of those extra out-degrees at random and added it to a random node that had fewer than the maximum out-degree. It repeated this process until the conditions in Equation 3 were met for each node.

One million random twelve-node networks were generated in this manner for each combination of $\min(k_{in})$ and $\max(k_{out})$. Each network was measured with the Krackhardt hierarchy function included in the *sna* R package. The distribution of these scores for each parameter combination as well as a sample network for each score were saved and available in the OSF repository. High, low, and medium hierarchy scores are reported in Table S12. Hierarchy score distributions and example networks and reported for each parameter combination in Tables S3 - S68. The example networks with the median hierarchy scores were used for Figures 5 and 6 in the main text.

The "$E \times H$" column shows the composite hierarchy score which is the graph efficiency ($E$) score multiplied by the graph hierarchy ($H$). The high composite scores are shown in Figure 6 in the main text.

Figure S3 shows the efficiency scores for each network. Figure S4 shows the low $H$ scores. Figure S5 shows the median $H$ scores. Figure S6 shows the high $H$ scores.

Figure S7 shows the low composite scores and Figure 6 shows the median composite scores.

These figures tell a similar story to that in Figure 6 in the main text. The model does not presuppose which network structures would be more common in the real world, but all of them show how hierarchy changes with the efficiency of monitoring and punishment.

| | | Graph | Graph hierarchy (H) | | | Composite $E \times H$ | | |
|---|---|---|---|---|---|---|---|---|
| min($k_{in}$) | max($k_{out}$) | efficiency (E) | (low) | (median) | (high) | (low) | (median) | (high) |
| 1 | 1 | 0.99 | 0.00 | 0.00 | 0.00 | 0.00 | 0.00 | 0.00 |
| 1 | 2 | 0.99 | 0.71 | 0.92 | 0.98 | 0.70 | 0.91 | 0.97 |
| 1 | 3 | 0.99 | 0.84 | 0.96 | 0.97 | 0.83 | 0.95 | 0.96 |
| 1 | 4 | 0.99 | 0.90 | 0.96 | 0.96 | 0.89 | 0.95 | 0.95 |
| 1 | 5 | 0.99 | 0.90 | 0.96 | 0.96 | 0.89 | 0.95 | 0.95 |
| 1 | 6 | 0.99 | 0.95 | 0.95 | 0.95 | 0.94 | 0.94 | 0.94 |
| 1 | 7 | 0.99 | 0.95 | 0.95 | 0.95 | 0.94 | 0.94 | 0.94 |
| 1 | 8 | 0.99 | 0.95 | 0.95 | 0.95 | 0.94 | 0.94 | 0.94 |
| 1 | 9 | 0.99 | 0.95 | 0.95 | 0.95 | 0.94 | 0.94 | 0.94 |
| 1 | 10 | 0.99 | 0.95 | 0.95 | 0.95 | 0.94 | 0.94 | 0.94 |
| 1 | 11 | 0.99 | 0.95 | 0.95 | 0.95 | 0.94 | 0.94 | 0.94 |
| 2 | 2 | 0.89 | 0.00 | 0.00 | 0.00 | 0.00 | 0.00 | 0.00 |
| 2 | 3 | 0.89 | 0.53 | 0.53 | 0.95* | 0.47 | 0.47 | 0.85 |
| 2 | 4 | 0.89 | 0.71 | 0.71 | 0.94 | 0.63 | 0.63 | 0.84 |
| 2 | 5 | 0.89 | 0.78 | 0.78 | 0.93 | 0.69 | 0.69 | 0.83 |
| 2 | 6 | 0.89 | 0.84 | 0.84 | 0.92 | 0.75 | 0.75 | 0.82 |
| 2 | 7 | 0.89 | 0.84 | 0.84 | 0.92 | 0.75 | 0.75 | 0.82 |
| 2 | 8 | 0.89 | 0.90 | 0.90 | 0.90 | 0.80 | 0.80 | 0.80 |
| 2 | 9 | 0.89 | 0.90 | 0.90 | 0.90 | 0.80 | 0.80 | 0.80 |
| 2 | 10 | 0.89 | 0.90 | 0.90 | 0.90 | 0.80 | 0.80 | 0.80 |
| 2 | 11 | 0.89 | 0.90 | 0.90 | 0.90 | 0.80 | 0.80 | 0.80 |
| 3 | 3 | 0.79 | 0.00 | 0.00 | 0.00 | 0.00 | 0.00 | 0.00 |
| 3 | 4 | 0.79 | 0.43 | 0.43 | 0.84* | 0.34 | 0.34 | 0.66 |
| 3 | 5 | 0.79 | 0.53 | 0.53 | 0.90* | 0.42 | 0.42 | 0.71 |
| 3 | 6 | 0.79 | 0.71 | 0.71 | 0.88 | 0.56 | 0.56 | 0.70 |
| 3 | 7 | 0.79 | 0.71 | 0.71 | 0.88 | 0.56 | 0.56 | 0.70 |
| 3 | 8 | 0.79 | 0.78 | 0.78 | 0.87 | 0.62 | 0.62 | 0.69 |
| 3 | 9 | 0.79 | 0.84 | 0.84 | 0.84 | 0.66 | 0.66 | 0.66 |
| 3 | 10 | 0.79 | 0.84 | 0.84 | 0.84 | 0.66 | 0.66 | 0.66 |
| 3 | 11 | 0.79 | 0.84 | 0.84 | 0.84 | 0.66 | 0.66 | 0.66 |

| | | Graph | Graph hierarchy (H) | | | Composite $E \times H$ | | |
|---|---|---|---|---|---|---|---|---|
| $\min(k_{in})$ | $\max(k_{out})$ | efficiency (E) | (low) | (median) | (high) | (low) | (median) | (high) |
| 4 | 4 | 0.69 | 0.00 | 0.00 | 0.00 | 0.00 | 0.00 | 0.00 |
| 4 | 5 | 0.69 | 0.31 | 0.31 | 0.75* | 0.21 | 0.21 | 0.52 |
| 4 | 6 | 0.69 | 0.53 | 0.53 | 0.78* | 0.37 | 0.37 | 0.54 |
| 4 | 7 | 0.69 | 0.63 | 0.63 | 0.63 | 0.43 | 0.43 | 0.43 |
| 4 | 8 | 0.69 | 0.71 | 0.71 | 0.71 | 0.49 | 0.49 | 0.49 |
| 4 | 9 | 0.69 | 0.71 | 0.71 | 0.80* | 0.49 | 0.49 | 0.55 |
| 4 | 10 | 0.69 | 0.78 | 0.78 | 0.78 | 0.54 | 0.54 | 0.54 |
| 4 | 11 | 0.69 | 0.78 | 0.78 | 0.78 | 0.54 | 0.54 | 0.54 |
| 5 | 5 | 0.60 | 0.00 | 0.00 | 0.00 | 0.00 | 0.00 | 0.00 |
| 5 | 6 | 0.60 | 0.31 | 0.31 | 0.31 | 0.19 | 0.19 | 0.19 |
| 5 | 7 | 0.60 | 0.43 | 0.43 | 0.43 | 0.26 | 0.26 | 0.26 |
| 5 | 8 | 0.60 | 0.53 | 0.53 | 0.65 | 0.32 | 0.32 | 0.32 |
| 5 | 9 | 0.60 | 0.63 | 0.63 | 0.63 | 0.38 | 0.38 | 0.38 |
| 5 | 10 | 0.60 | 0.71 | 0.71 | 0.71 | 0.43 | 0.43 | 0.43 |
| 5 | 11 | 0.60 | 0.71 | 0.71 | 0.71 | 0.43 | 0.43 | 0.43 |
| 6 | 6 | 0.50 | 0.00 | 0.00 | 0.00 | 0.00 | 0.00 | 0.00 |
| 6 | 7 | 0.50 | 0.17 | 0.17 | 0.17 | 0.09 | 0.09 | 0.09 |
| 6 | 8 | 0.50 | 0.43 | 0.43 | 0.43 | 0.22 | 0.22 | 0.22 |
| 6 | 9 | 0.50 | 0.53 | 0.53 | 0.53 | 0.27 | 0.27 | 0.27 |
| 6 | 10 | 0.50 | 0.53 | 0.53 | 0.65 | 0.27 | 0.27 | 0.33 |
| 6 | 11 | 0.50 | 0.63 | 0.63 | 0.63 | 0.32 | 0.32 | 0.32 |
| 7 | 7 | 0.40 | 0.00 | 0.00 | 0.00 | 0.00 | 0.00 | 0.00 |
| 7 | 8 | 0.40 | 0.17 | 0.17 | 0.17 | 0.07 | 0.07 | 0.07 |
| 7 | 9 | 0.40 | 0.31 | 0.31 | 0.31 | 0.12 | 0.12 | 0.12 |
| 7 | 10 | 0.40 | 0.43 | 0.43 | 0.43 | 0.17 | 0.17 | 0.17 |
| 7 | 11 | 0.40 | 0.53 | 0.53 | 0.53 | 0.21 | 0.21 | 0.21 |
| 8 | 8 | 0.30 | 0.00 | 0.00 | 0.00 | 0.00 | 0.00 | 0.00 |
| 8 | 9 | 0.30 | 0.17 | 0.17 | 0.17 | 0.05 | 0.05 | 0.05 |
| 8 | 10 | 0.30 | 0.31 | 0.31 | 0.31 | 0.09 | 0.09 | 0.09 |
| 8 | 11 | 0.30 | 0.43 | 0.43 | 0.43 | 0.13 | 0.13 | 0.13 |
| 9 | 9 | 0.20 | 0.00 | 0.00 | 0.00 | 0.00 | 0.00 | 0.00 |
| 9 | 10 | 0.20 | 0.17 | 0.17 | 0.17 | 0.03 | 0.03 | 0.03 |
| 9 | 11 | 0.20 | 0.31 | 0.31 | 0.31 | 0.06 | 0.06 | 0.06 |

| | | Graph | Graph hierarchy (H) | | | Composite $E \times H$ | | |
|---|---|---|---|---|---|---|---|---|
| min$(k_{in})$ | max$(k_{out})$ | efficiency (E) | (low) | (median) | (high) | (low) | (median) | (high) |
| 10 | 10 | 0.10 | 0.00 | 0.00 | 0.00 | 0.00 | 0.00 | 0.00 |
| 10 | 11 | 0.10 | 0.17 | 0.17 | 0.17 | 0.02 | 0.02 | 0.02 |
| 11 | 11 | 0.00 | 0.00 | 0.00 | 0.00 | 0.00 | 0.00 | 0.00 |

Table S2: Network properties for twelve-node socially efficient networks where universal threshold contribution is a Nash equilibrium and pure contributors are maximized. The first two columns give the minimum links needed to incentivize threshold contributions and the maximum number of out-links per node that can be paid for with a threshold contribution. Measures of graph hierarchy and graph efficiency were calculated from simulated graphs, as described in the main text. Graph efficiency and the lower bound on graph hierarchy were also calculated analytically, as described in Appendices S8 and S9. The calculated and simulated values agree. Bold numbers for simulated hierarchy scores indicate where there is a difference between the high and low graph hierarchy scores. Bold numbers in the calculated hierarchy scores indicate where the simulated graphs did not find the lowest hierarchical case.

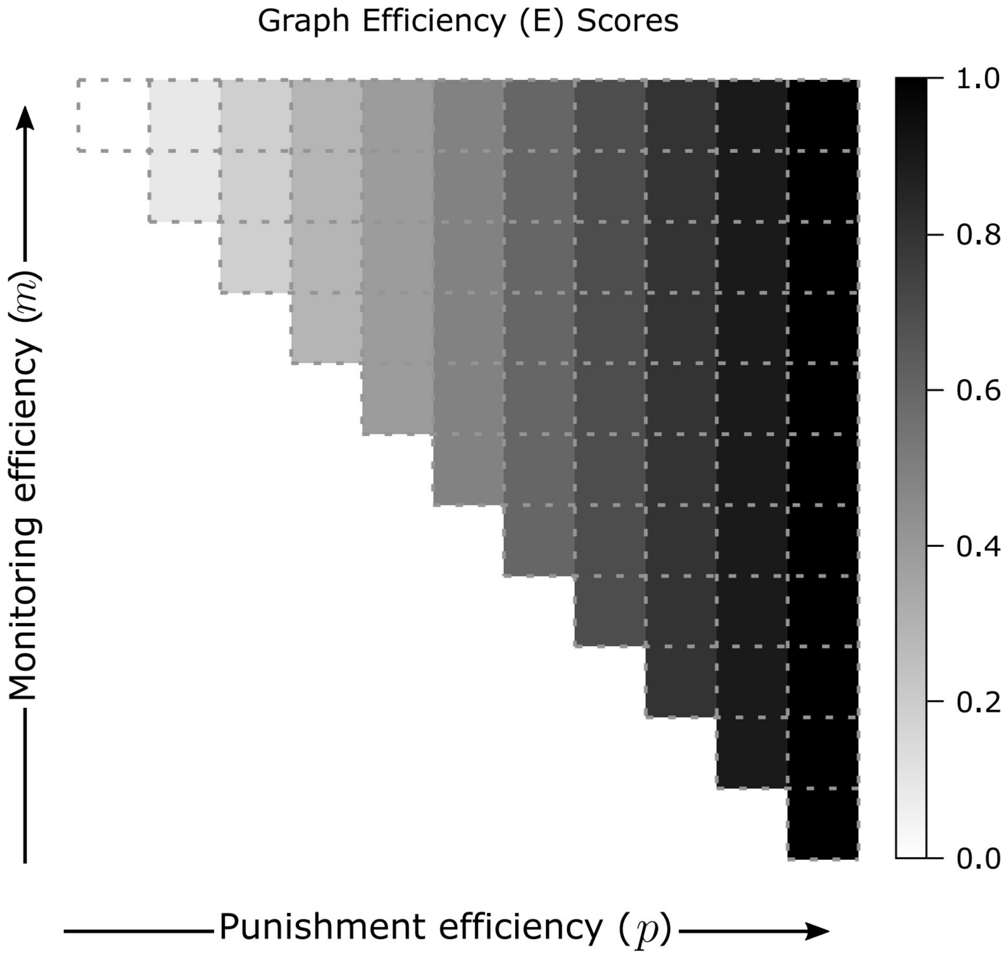


Figure S3: Graph efficiency scores for twelve-node networks shown in Figure 5 in the main text, but also for any socially efficient network with those parameter values. Graph efficiency is a component of the composite scores in Figures 6, S7, and 6. Graph efficiency increases with punishment efficiency, but does not change with monitoring efficiency. This is consistent with the analytic calculation of graph efficiency from Appendix S8 and from Equation 5 in the main text.

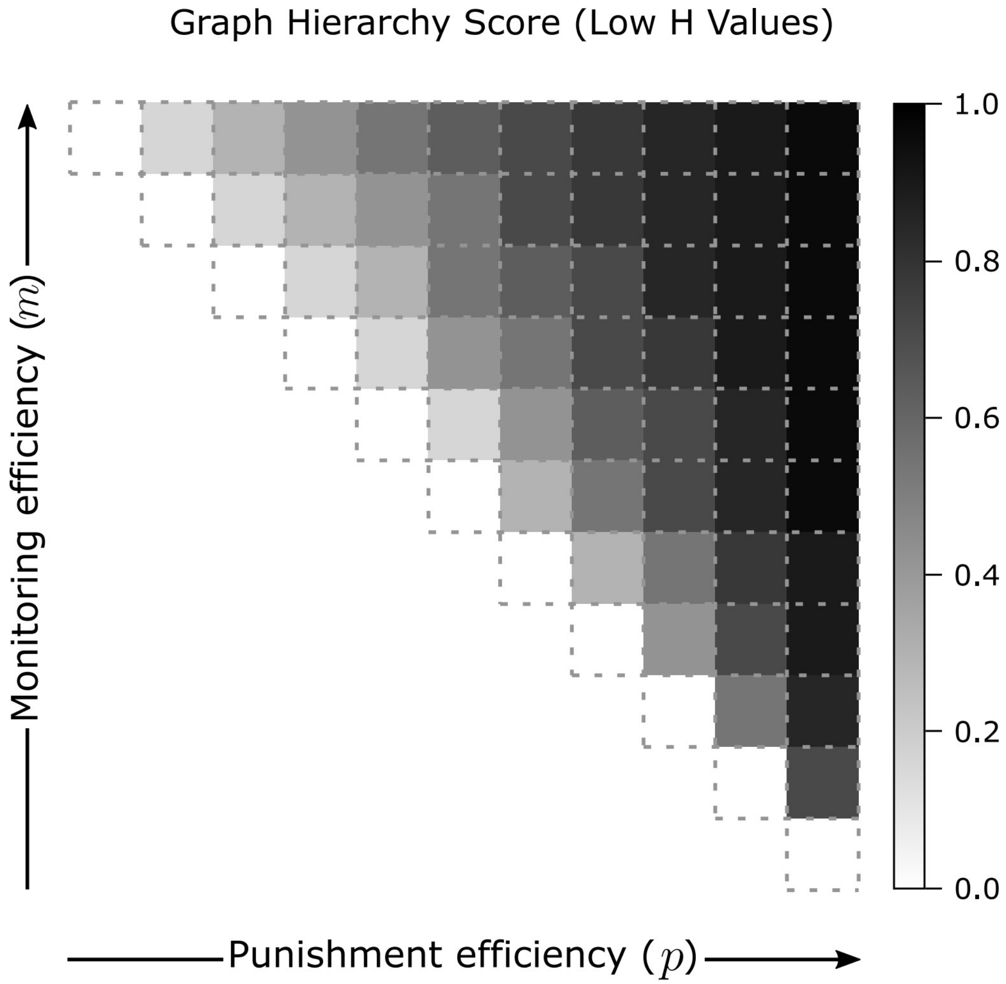


Figure S4: Graph hierarchy scores for socially efficient twelve-node networks assuming the lowest possible hierarchy score as calculated through the method in Appendix S9, listed in Table S12, and found for the specific networks shown in Tables S3 - S68. These lower-bound graph hierarchy scores are component of the composite scores in Figure S7. Lower-bound hierarchy scores increase with both monitoring and punishment efficiency consistent with the analytic derivation in Appendix S9.

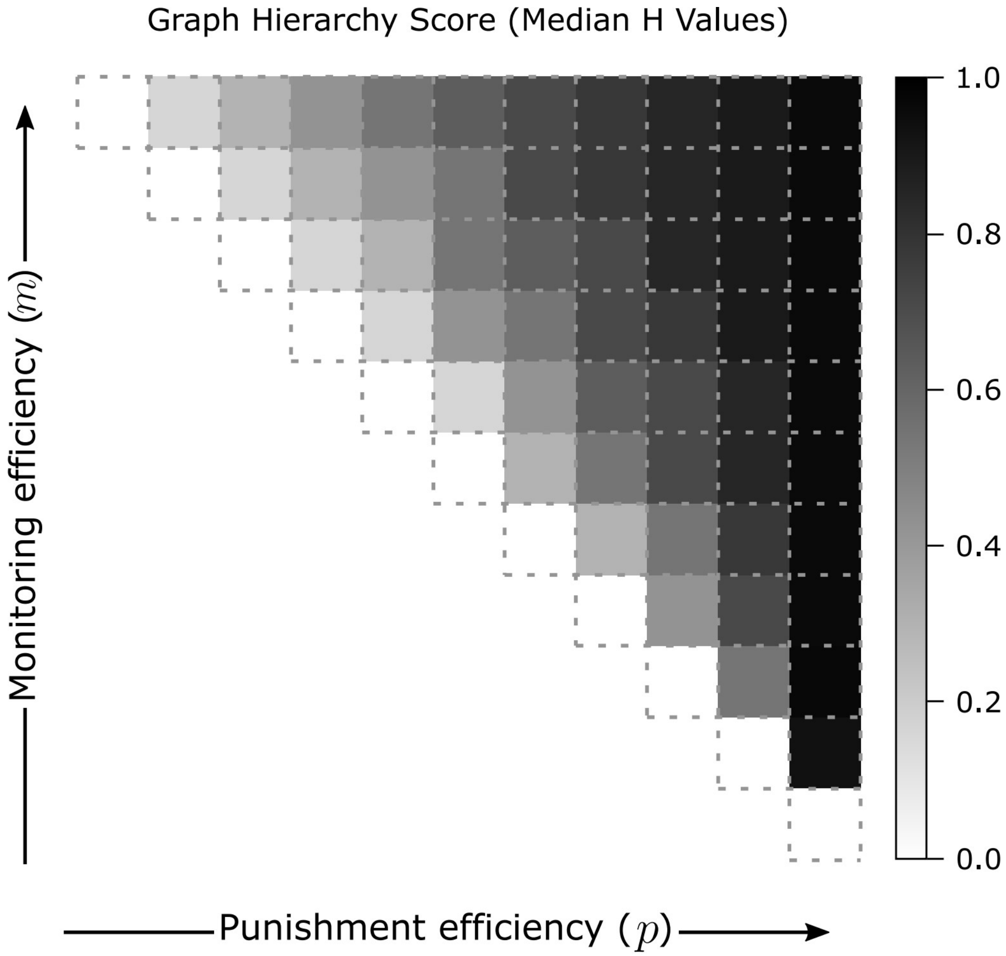


Figure S5: Graph hierarchy scores for socially efficient twelve-node networks assuming the median hierarchy score from randomly generated networks as listed in Table S12, and shown in Tables S3 - S68 for each parameter value. With a few exceptions, the median networks have the same hierarchy scores as the lower-bound networks. Therefore, this plot is similar to Figure S4 and hierarchy scores also increase with both punishment and monitoring efficiency. These graph hierarchy scores are component of the composite scores in Figure 6.

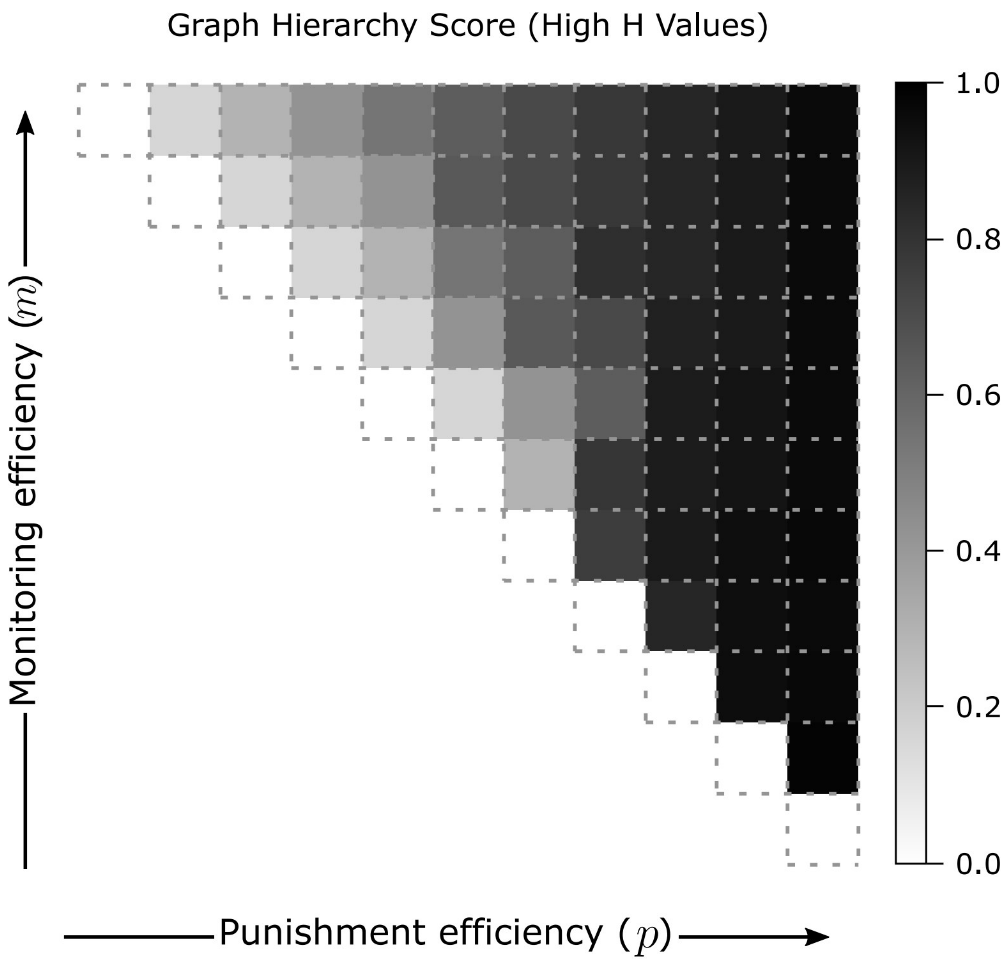


Figure S6: Graph hierarchy scores for socially efficient twelve-node networks assuming the highest hierarchy score found from both a random and a target search of possible networks. The relationship between hierarchy score, monitoring efficiency and punishment efficiency is more complicated than for the lower and median $H$ values in Figures S4 and S6, but still generally increase with both monitoring and punishment efficiencies. These graph hierarchy scores are component of the composite scores in Figure 6 in the main text.

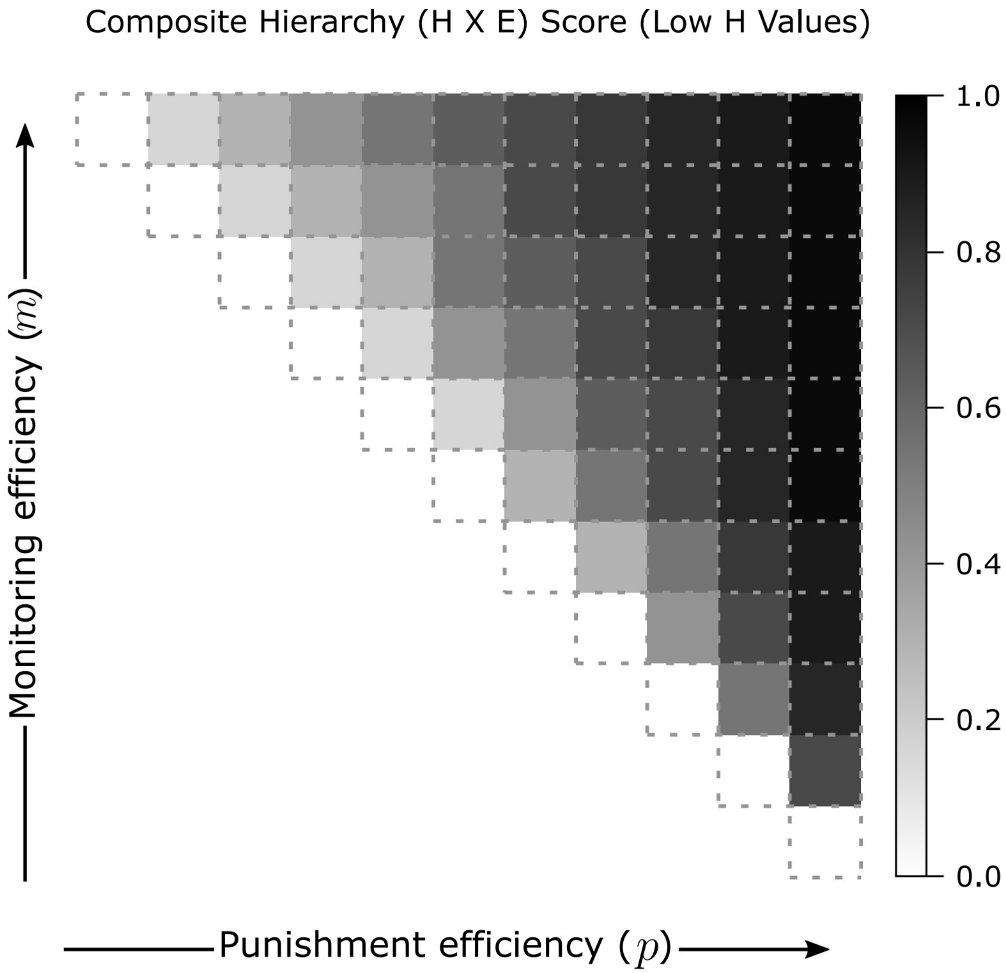


Figure S7: Composite hierarchy scores for socially efficient twelve-node networks assuming the lowest possible hierarchy score as calculated through the method in Appendix S9. The composite is made up of the efficiency scores in Figure S3 multiplied by the hierarchy scores in Figure S4.

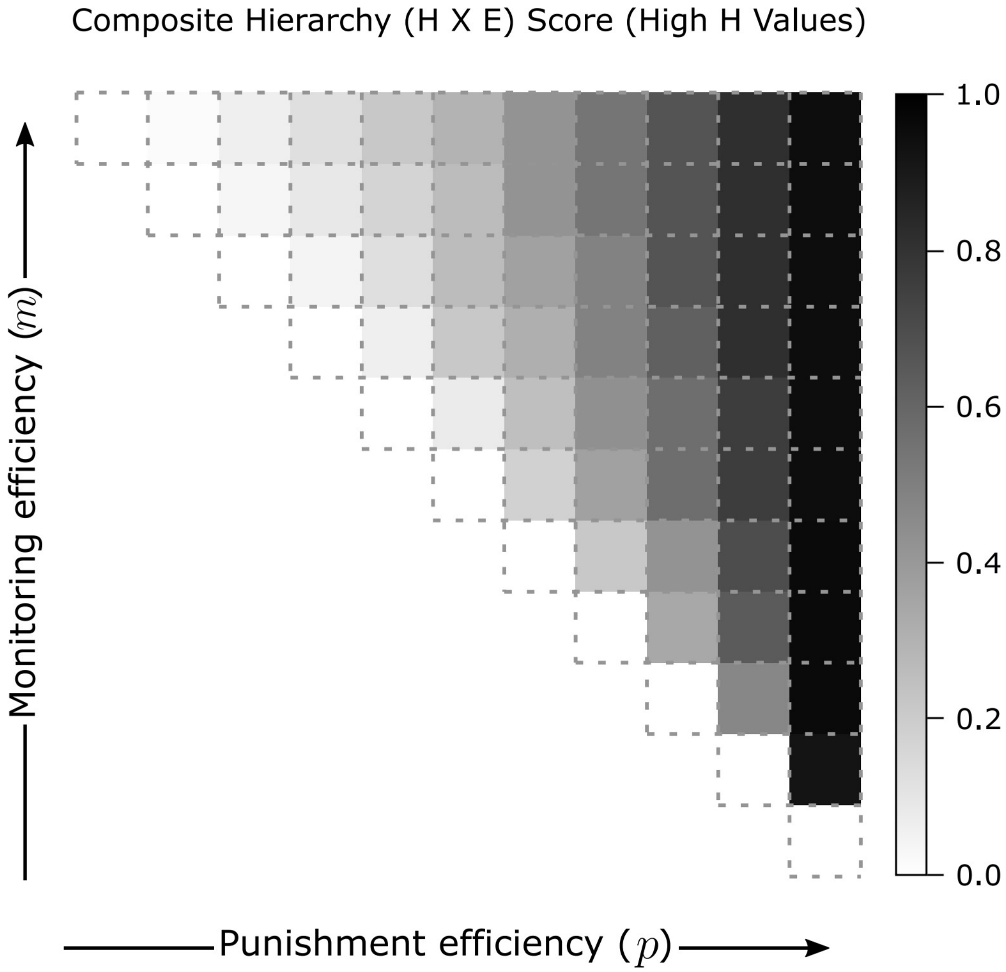


Figure S8: Composite hierarchy scores for socially efficient twelve-node networks assuming the highest found hierarchy score as found through random and targeted search. The composite is made up of the efficiency scores in Figure S3 multiplied by the hierarchy scores in Figure S6.

Tables S3 to S68 show information for each combination of min$(k_{in})$ and max$(k_{out})$ for socially efficient twelve-node networks where universal threshold contribution is a Nash equilibrium. The tables show:

- The number of nodes, $N = 12$;
- The minimum in-degree required to incentivize information for each individual, min$(k_{in})$, as calculated according to Equation 2;
- The maximum out-degree for each node under the contribution threshold, max$(k_{out})$, as calculated according to Equation 3;
- The range of the monitoring efficiency and punishment efficiency parameters, $m$ and $p$, under which the networks are socially efficient, also calculated from Equations 2 and 3;
- The maximum number of pure contributors under those conditions, calculated according to the procedure described in Appendix S7;
- The graph efficiency, $E$, score of these networks as calculated according to the procedure in Appendix S8;
- The minimum graph hierarchy, $H$, score of these networks as calculated according to the procedure in Appendix S9;
- A plot showing the distribution of $H$ scores for a sample of one-million randomly generated networks under the min$(k_{in})$ and max$(k_{out})$ constraints with pure contributors maximized;
- Low, median, and high $H$ scores from Table S12;
- Example networks for the low, median and high $H$ scores. Pure contributors are blue and the rest of the individuals are red. The number in each node is that node's out degree. All nodes have the same in-degree of min$(k_{in})$. Only one network is shown for each numerical $H$ score.

| $N = 12$ | $\min(k_{in}) = 1$ | $\max(k_{out}) = 1$ |
|---|---|---|
| Parameter range: | $\frac{3}{c_t - c_p} < m < \frac{3}{c_t - c_p}$ | $\frac{c_t}{c_p} - \frac{b}{11} < p < \frac{c_t}{2c_p} - \frac{b}{11}$ |
| Max pure contributors ($C$): | $N - \left\lceil \frac{N \min(k_{in})}{\max(k_{out})} \right\rceil = 12 - \left\lceil \frac{12}{1} \right\rceil = 0$ | |
| Graph Efficiency ($E$): | $E = 1 - \frac{N \min(k_{in}) - N + 1}{(N-1)^2} = 0.9917$ | |
| Minimum Graph Hierarchy: | $\frac{2N - 2C}{2N - C - 1} = 0/11 = 0.0000$ | |
| Graph hierarchy score distribution from 1,000,000 random samples: 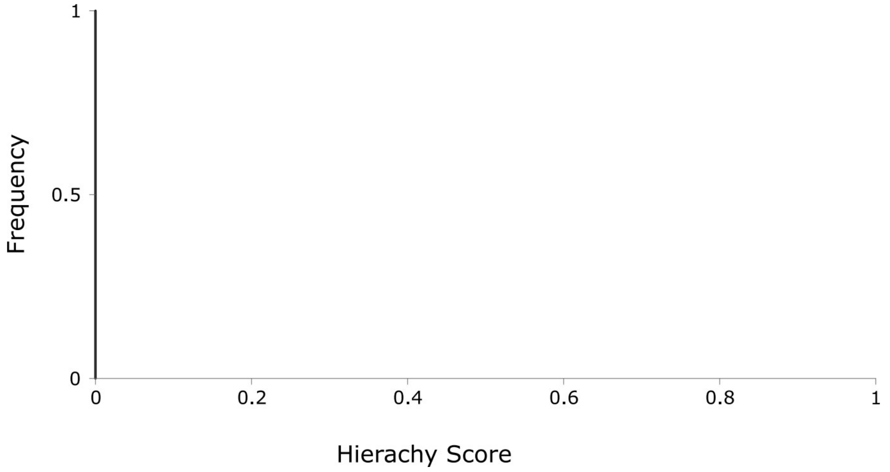 | | |
| Low H score 0.0000 | Median H score 0.0000 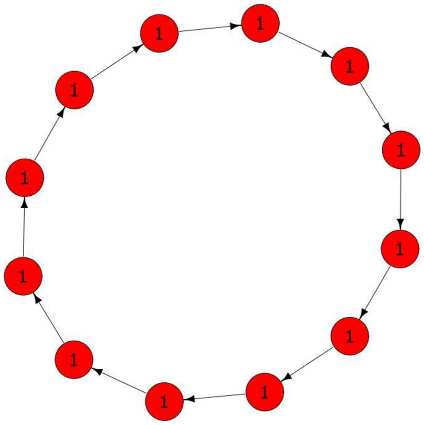 | High H score 0.0000 |

Table S3: Properties of socially efficient networks when $\min(k_{in}) = 1$, $\max(k_{out}) = 1$, universal threshold contribution is a Nash equilibrium, and pure contributors are maximized.

<table>
<tr><td>$N = 12$</td><td>$\min(k_{in}) = 1$</td><td>$\max(k_{out}) = 2$</td></tr>
<tr><td>Parameter range:</td><td>$\frac{4}{c_t - c_p} < m < \frac{4}{c_t - c_p}$</td><td>$\frac{c_t}{c_p} - \frac{b}{11} < p < \frac{c_t}{2c_p} - \frac{b}{11}$</td></tr>
<tr><td>Max pure contributors ($N - C$):</td><td colspan="2">$N - \left\lceil \frac{N\min(k_{in})}{\max(k_{out})} \right\rceil = 12 - \left\lceil \frac{12}{2} \right\rceil = 6$</td></tr>
<tr><td>Graph Efficiency ($E$):</td><td colspan="2">$E = 1 - \frac{N\min(k_{in}) - N + 1}{(N-1)^2} = 0.9917$</td></tr>
<tr><td>Minimum Graph Hierarchy:</td><td colspan="2">$\frac{2N-2C}{2N-C-1} = 12/17 = 0.7059$</td></tr>
<tr><td colspan="3">Graph hierarchy score distribution from 1,000,000 random samples:<br>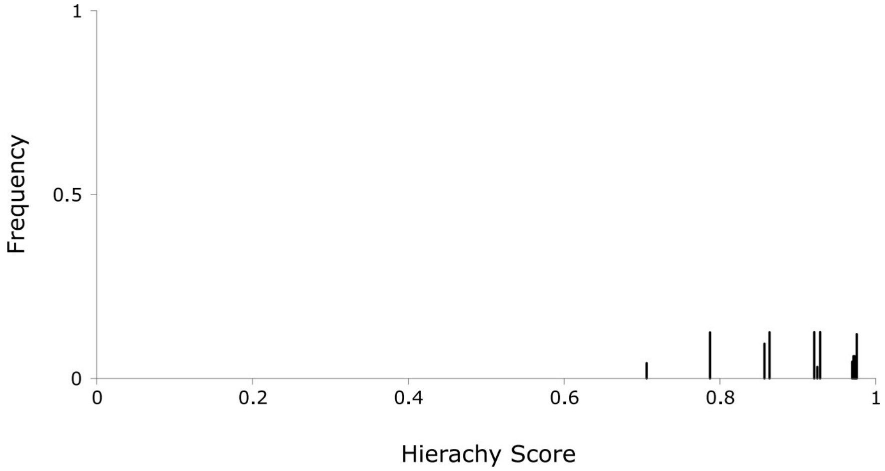
</td></tr>
<tr><td>Low H score<br>0.7059<br>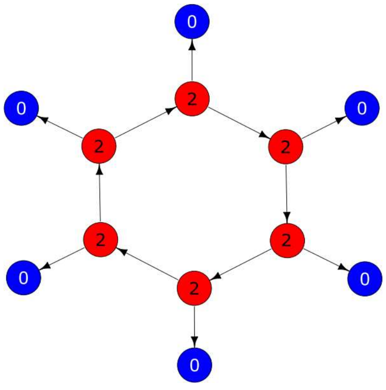</td><td>Median H score<br>0.9211<br>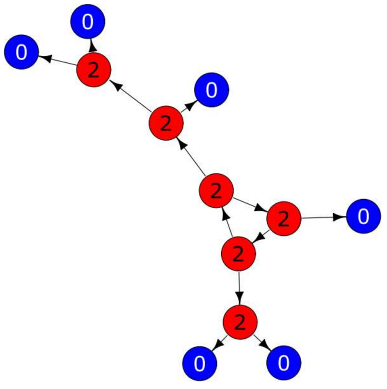</td><td>High H score<br>0.9756<br>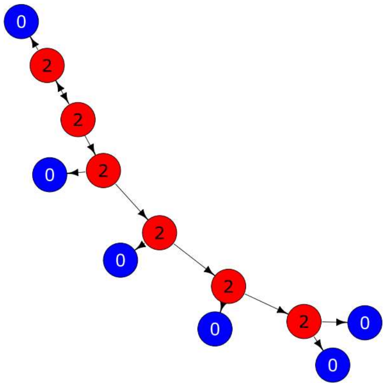</td></tr>
</table>

Table S4: Properties of socially efficient networks when $\min(k_{in}) = 1$, $\max(k_{out}) = 2$, universal threshold contribution is a Nash equilibrium, and pure contributors are maximized. All possible networks and $H$ scores for these conditions are in Appendix S13.

| | | |
|---|---|---|
| $N = 12$ | $\min(k_{in}) = 1$ | $\max(k_{out}) = 3$ |
| Parameter range: | $\frac{4}{c_t - c_p} < m < \frac{4}{c_t - c_p}$ | $\frac{c_t}{c_p} - \frac{b}{11} < p < \frac{c_t}{2c_p} - \frac{b}{11}$ |
| Max pure contributors ($C$): | $N - \left\lceil \frac{N \min(k_{in})}{\max(k_{out})} \right\rceil = 12 - \left\lceil \frac{12}{3} \right\rceil = 8$ | |
| Graph Efficiency ($E$): | $E = 1 - \frac{N \min(k_{in}) - N + 1}{(N-1)^2} = 0.9917$ | |
| Minimum Graph Hierarchy: | $\frac{2N - 2C}{2N - C - 1} = 16/19 = 0.8421$ | |
| Graph hierarchy score distribution from 1,000,000 random samples: 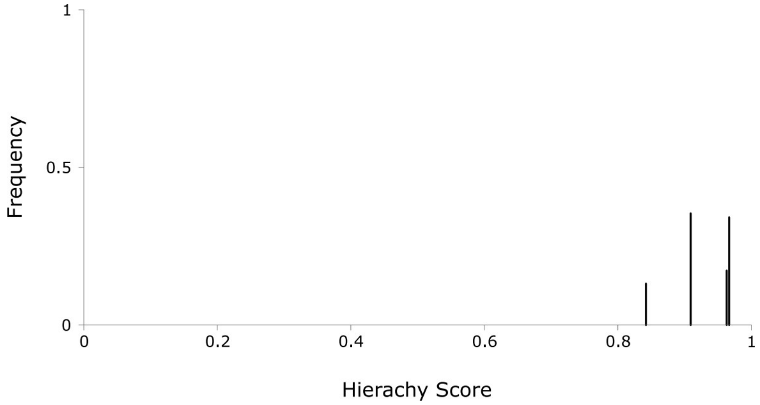  | | |
| Low H score 0.8421 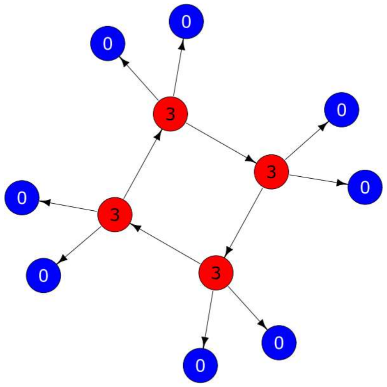 | Median H score 0.9630 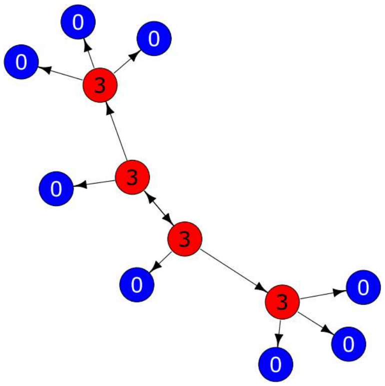 | High H score 0.9667 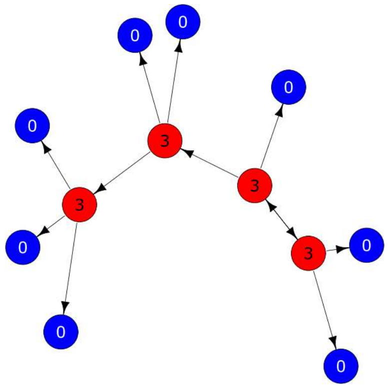 |

Table S5: Properties of socially efficient networks when $\min(k_{in}) = 1$, $\max(k_{out}) = 3$, universal threshold contribution is a Nash equilibrium, and pure contributors are maximized. All possible networks and $H$ scores for these conditions are in Appendix S13.

| $N = 12$ | $\min(k_{in}) = 1$ | $\max(k_{out}) = 4$ |
| --- | --- | --- |
| Parameter range: | $\frac{4}{c_t - c_p} < m < \frac{4}{c_t - c_p}$ | $\frac{c_t}{c_p} - \frac{b}{11} < p < \frac{c_t}{2c_p} - \frac{b}{11}$ |
| Max pure contributors ($C$): | $N - \left\lceil \frac{N \min(k_{in})}{\max(k_{out})} \right\rceil = 12 - \left\lceil \frac{12}{4} \right\rceil = 9$ | |
| Graph Efficiency ($E$): | $E = 1 - \frac{N \min(k_{in}) - N + 1}{(N-1)^2} = 0.9917$ | |
| Minimum Graph Hierarchy: | $\frac{2N - 2C}{2N - C - 1} = 18/20 = 0.9000$ | |
| Graph hierarchy score distribution from 1,000,000 random samples: 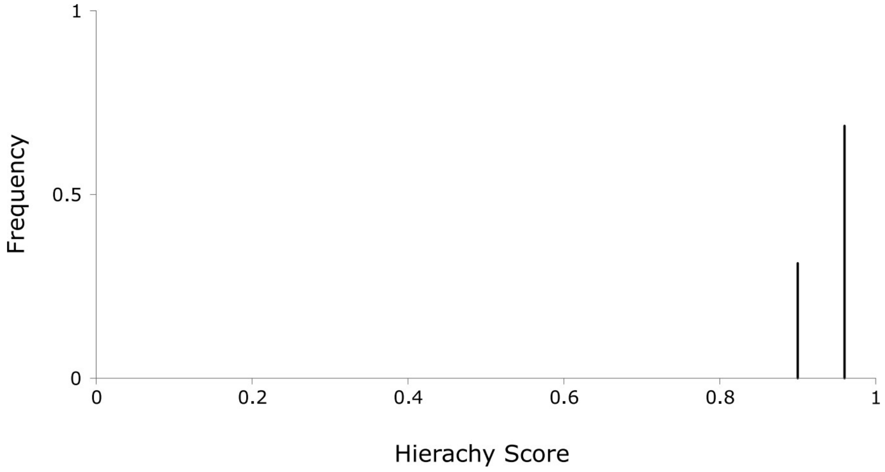  | | |
| Low H score<br>0.9000<br>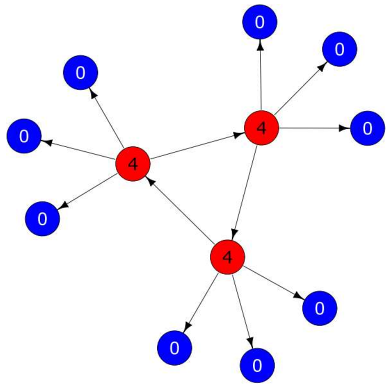  | Median H score<br>0.9600 | High H score<br>0.9600<br>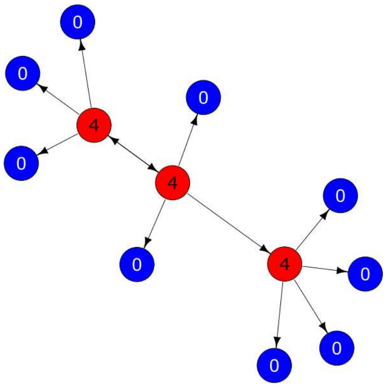  |

Table S6: Properties of socially efficient networks when $\min(k_{in}) = 1$, $\max(k_{out}) = 4$, universal threshold contribution is a Nash equilibrium, and pure contributors are maximized. All possible networks and $H$ scores for these conditions are in Appendix S13.

| $N = 12$ | $\min(k_{in}) = 1$ | $\max(k_{out}) = 5$ |
|---|---|---|
| Parameter range: | $\frac{4}{c_t - c_p} < m < \frac{4}{c_t - c_p}$ | $\frac{c_t}{c_p} - \frac{b}{11} < p < \frac{c_t}{2c_p} - \frac{b}{11}$ |
| Max pure contributors ($C$): | $N - \left\lceil \frac{N \min(k_{in})}{\max(k_{out})} \right\rceil = 12 - \left\lceil \frac{12}{5} \right\rceil = 9$ | |
| Graph Efficiency ($E$): | $E = 1 - \frac{N \min(k_{in}) - N + 1}{(N-1)^2} = 0.9917$ | |
| Minimum Graph Hierarchy: | $\frac{2N - 2C}{2N - C - 1} = 18/20 = 0.9000$ | |

Graph hierarchy score distribution from 1,000,000 random samples:

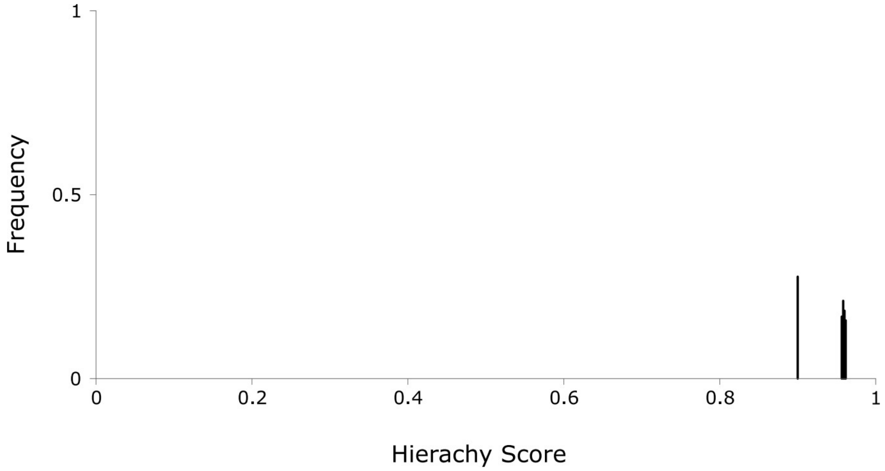


| Low H score | Median H score | High H score |
|---|---|---|
| 0.9000 | 0.9583 | 0.9615 |
| 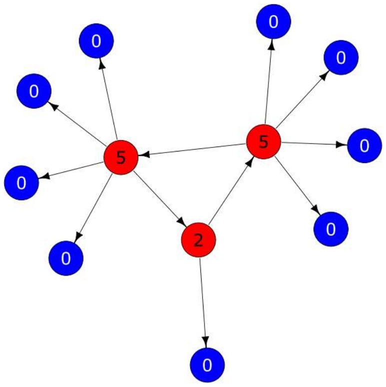 | 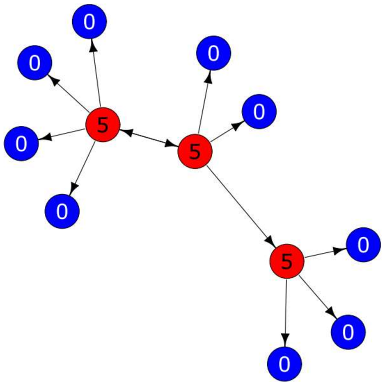 | 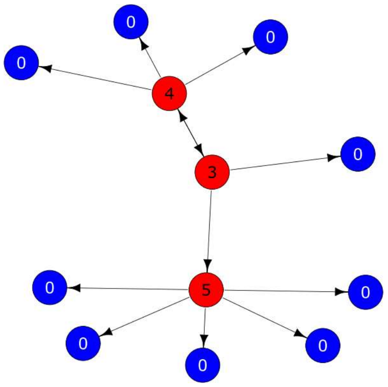 |

Table S7: Properties of socially efficient networks when $\min(k_{in}) = 1$, $\max(k_{out}) = 5$, universal threshold contribution is a Nash equilibrium, and pure contributors are maximized. All possible networks and $H$ scores for these conditions are in Appendix S13.

| | | |
|---|---|---|
| $N = 12$ | $\min(k_{in}) = 1$ | $\max(k_{out}) = 6$ |
| Parameter range: | $\frac{4}{c_t - c_p} < m < \frac{4}{c_t - c_p}$ | $\frac{c_t}{c_p} - \frac{b}{11} < p < \frac{c_t}{2c_p} - \frac{b}{11}$ |
| Max pure contributors ($C$): | $N - \left\lceil \frac{N\min(k_{in})}{\max(k_{out})} \right\rceil = 12 - \left\lceil \frac{12}{6} \right\rceil = 10$ | |
| Graph Efficiency ($E$): | $E = 1 - \frac{N\min(k_{in}) - N + 1}{(N-1)^2} = 0.9917$ | |
| Minimum Graph Hierarchy: | $\frac{2N-2C}{2N-C-1} = 20/21 = 0.9524$ | |

Graph hierarchy score distribution from 1,000,000 random samples:

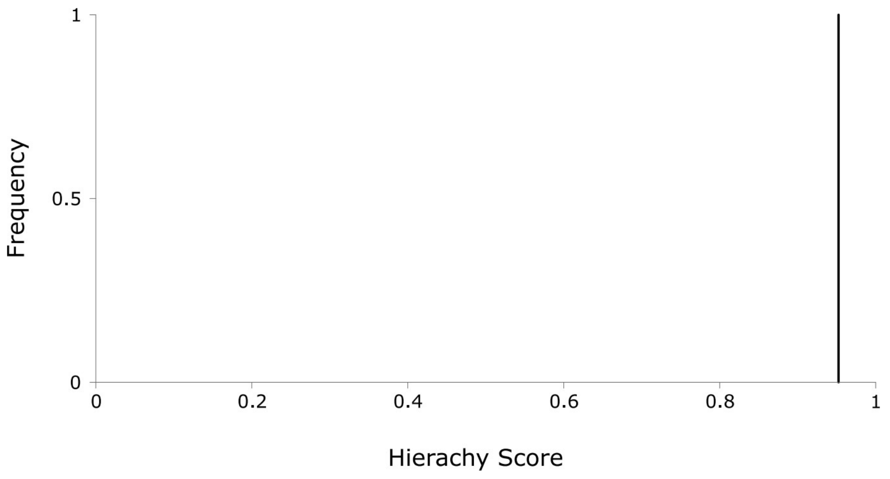


| Low H score | Median H score | High H score |
|---|---|---|
| 0.9524 | 0.9524 | 0.9524 |

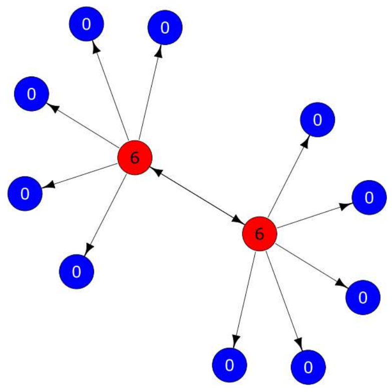


Table S8: Properties of socially efficient networks when $\min(k_{in}) = 1$, $\max(k_{out}) = 6$, universal threshold contribution is a Nash equilibrium, and pure contributors are maximized. All possible networks and $H$ scores for these conditions are in Appendix S13.

| $N = 12$ | $\min(k_{in}) = 1$ | $\max(k_{out}) = 7$ |
|---|---|---|
| Parameter range: | $\frac{4}{c_t - c_p} < m < \frac{4}{c_t - c_p}$ | $\frac{c_t}{c_p} - \frac{b}{11} < p < \frac{c_t}{2c_p} - \frac{b}{11}$ |
| Max pure contributors ($C$): | $N - \left\lceil \frac{N \min(k_{in})}{\max(k_{out})} \right\rceil = 12 - \left\lceil \frac{12}{7} \right\rceil = 10$ | |
| Graph Efficiency ($E$): | $E = 1 - \frac{N \min(k_{in}) - N + 1}{(N-1)^2} = 0.9917$ | |
| Minimum Graph Hierarchy: | $\frac{2N - 2C}{2N - C - 1} = 20/21 = 0.9524$ | |
| Graph hierarchy score distribution from 1,000,000 random samples: | | |
| Low H score 0.9524 | Median H score 0.9524 | High H score 0.9524 |

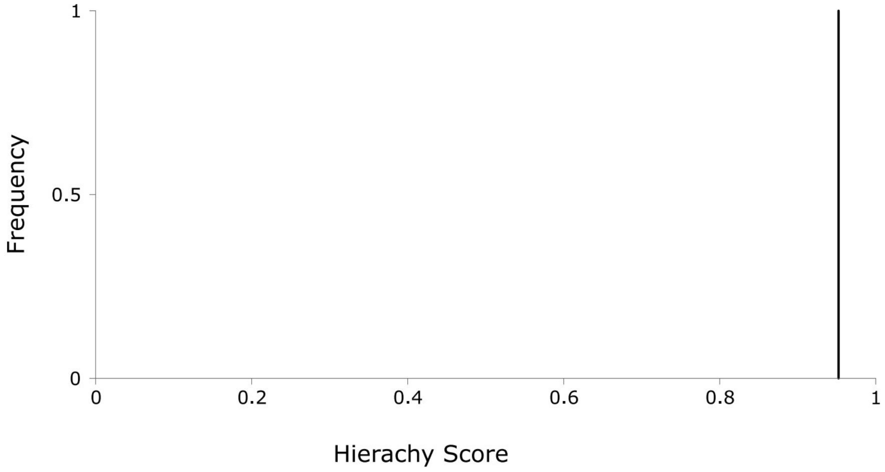


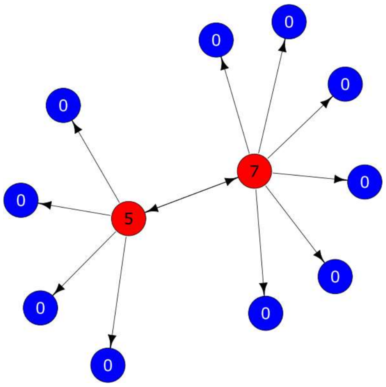


Table S9: Properties of socially efficient networks when $\min(k_{in}) = 1$, $\max(k_{out}) = 7$, universal threshold contribution is a Nash equilibrium, and pure contributors are maximized. All possible networks and $H$ scores for these conditions are in Appendix S13.

| $N = 12$ | $\min(k_{in}) = 1$ | $\max(k_{out}) = 8$ |
|---|---|---|
| Parameter range: | $\frac{4}{c_t - c_p} < m < \frac{4}{c_t - c_p}$ | $\frac{c_t}{c_p} - \frac{b}{11} < p < \frac{c_t}{2c_p} - \frac{b}{11}$ |
| Max pure contributors ($C$): | $N - \left\lceil \frac{N \min(k_{in})}{\max(k_{out})} \right\rceil = 12 - \left\lceil \frac{12}{8} \right\rceil = 10$ | |
| Graph Efficiency ($E$): | $E = 1 - \frac{N \min(k_{in}) - N + 1}{(N-1)^2} = 0.9917$ | |
| Minimum Graph Hierarchy: | $\frac{2N-2C}{2N-C-1} = 20/21 = 0.9524$ | |

Graph hierarchy score distribution from 1,000,000 random samples:

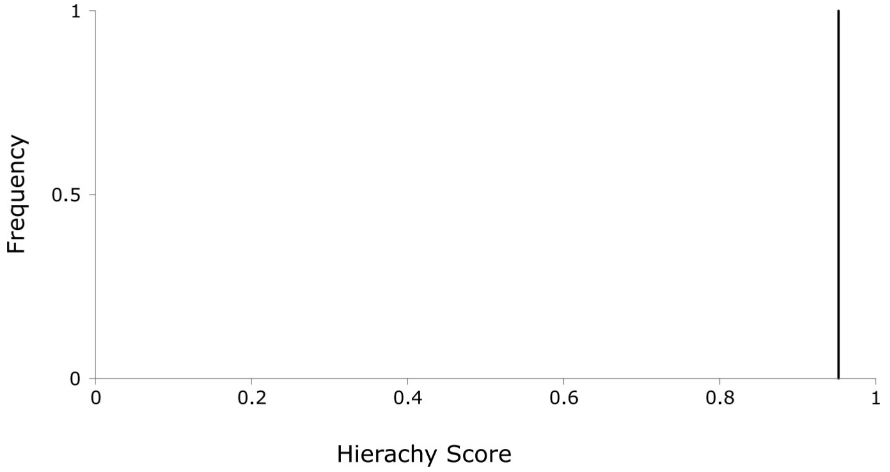


| Low H score | Median H score | High H score |
|---|---|---|
| 0.9524 | 0.9524 | 0.9524 |

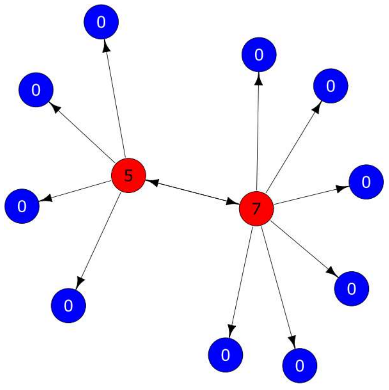


Table S10: Properties of socially efficient networks when $\min(k_{in}) = 1$, $\max(k_{out}) = 8$, universal threshold contribution is a Nash equilibrium, and pure contributors are maximized. All possible networks and $H$ scores for these conditions are in Appendix S13.

| $N = 12$ | $\min(k_{in}) = 1$ | $\max(k_{out}) = 9$ |
|---|---|---|
| Parameter range: | $\frac{4}{c_t - c_p} < m < \frac{4}{c_t - c_p}$ | $\frac{c_t}{c_p} - \frac{b}{11} < p < \frac{c_t}{2c_p} - \frac{b}{11}$ |
| Max pure contributors ($C$): | $N - \left\lceil \frac{N \min(k_{in})}{\max(k_{out})} \right\rceil = 12 - \left\lceil \frac{12}{9} \right\rceil = 10$ | |
| Graph Efficiency ($E$): | $E = 1 - \frac{N \min(k_{in}) - N + 1}{(N-1)^2} = 0.9917$ | |
| Minimum Graph Hierarchy: | $\frac{2N - 2C}{2N - C - 1} = 20/21 = 0.9524$ | |

Graph hierarchy score distribution from 1,000,000 random samples:

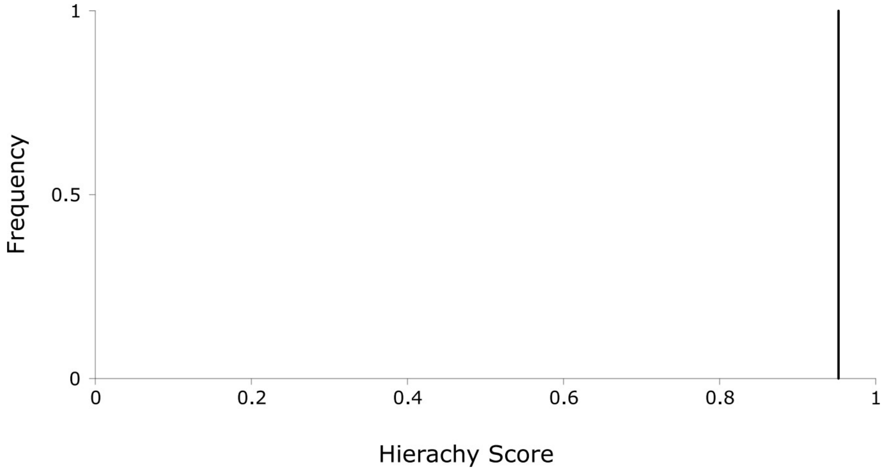


| Low H score | Median H score | High H score |
|---|---|---|
| 0.9524 | 0.9524 | 0.9524 |

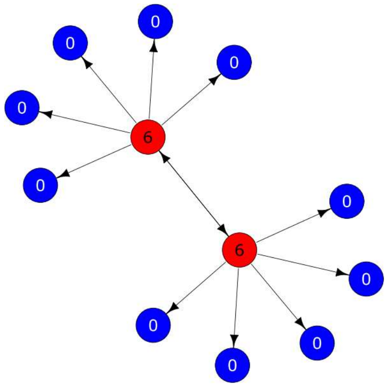


Table S11: Properties of socially efficient networks when $\min(k_{in}) = 1$, $\max(k_{out}) = 9$, universal threshold contribution is a Nash equilibrium, and pure contributors are maximized. All possible networks and $H$ scores for these conditions are in Appendix S13.

| | | |
|---|---|---|
| $N = 12$ | $\min(k_{in}) = 1$ | $\max(k_{out}) = 10$ |
| Parameter range: | $\frac{4}{c_t - c_p} < m < \frac{4}{c_t - c_p}$ | $\frac{c_t}{c_p} - \frac{b}{11} < p < \frac{c_t}{2c_p} - \frac{b}{11}$ |
| Max pure contributors ($C$): | $N - \left\lceil \frac{N\min(k_{in})}{\max(k_{out})} \right\rceil = 12 - \left\lceil \frac{12}{10} \right\rceil = 10$ | |
| Graph Efficiency ($E$): | $E = 1 - \frac{N\min(k_{in}) - N + 1}{(N-1)^2} = 0.9917$ | |
| Minimum Graph Hierarchy: | $\frac{2N-2C}{2N-C-1} = 20/21 = 0.9524$ | |

Graph hierarchy score distribution from 1,000,000 random samples:

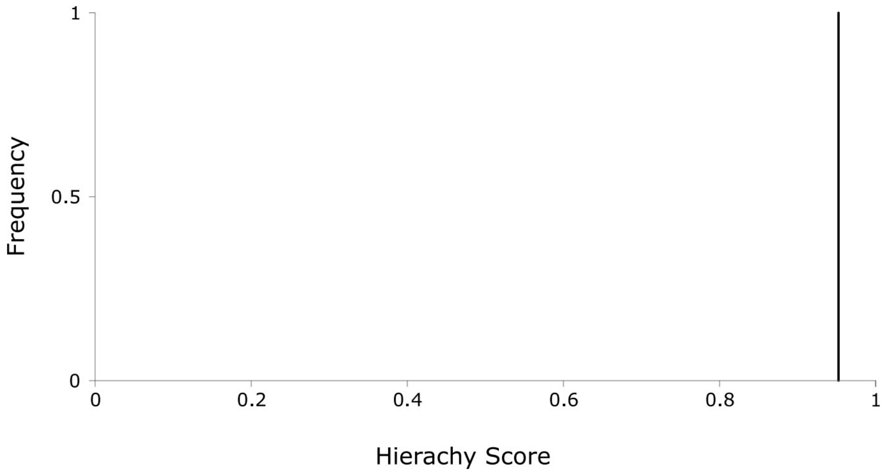


| Low H score | Median H score | High H score |
|---|---|---|
| 0.9524 | 0.9524 | 0.9524 |

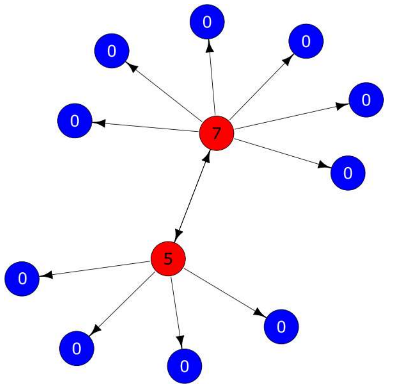


Table S12: Properties of socially efficient networks when $\min(k_{in}) = 1$, $\max(k_{out}) = 10$, universal threshold contribution is a Nash equilibrium, and pure contributors are maximized. All possible networks and $H$ scores for these conditions are in Appendix S13.

| | | |
|---|---|---|
| $N = 12$ | $\min(k_{in}) = 1$ | $\max(k_{out}) = 11$ |
| Parameter range: | $\frac{4}{c_t - c_p} < m < \frac{4}{c_t - c_p}$ | $\frac{c_t}{c_p} - \frac{b}{11} < p < \frac{c_t}{2c_p} - \frac{b}{11}$ |
| Max pure contributors ($C$): | $N - \left\lceil \frac{N \min(k_{in})}{\max(k_{out})} \right\rceil = 12 - \left\lceil \frac{12}{11} \right\rceil = 10$ | |
| Graph Efficiency ($E$): | $E = 1 - \frac{N \min(k_{in}) - N + 1}{(N-1)^2} = 0.9917$ | |
| Minimum Graph Hierarchy: | $\frac{2N - 2C}{2N - C - 1} = 20/21 = 0.9524$ | |

Graph hierarchy score distribution from 1,000,000 random samples:

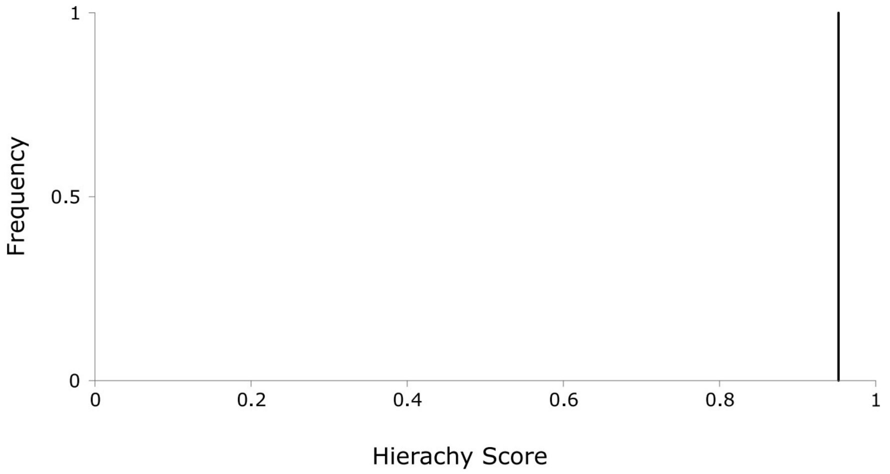


| Low H score | Median H score | High H score |
|---|---|---|
| 0.9524 | 0.9524 | 0.9524 |

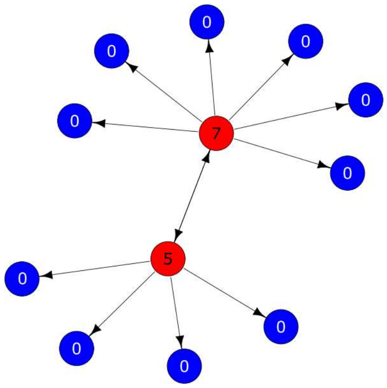

Table S13: Properties of socially efficient networks when $\min(k_{in}) = 1$, $\max(k_{out}) = 11$, universal threshold contribution is a Nash equilibrium, and pure contributors are maximized. All possible networks and $H$ scores for these conditions are in Appendix S13.

| | | |
|---|---|---|
| $N = 12$ | $\min(k_{in}) = 2$ | $\max(k_{out}) = 2$ |
| Parameter range: | $\frac{4}{c_t - c_p} < m < \frac{4}{c_t - c_p}$ | $\frac{c_t}{c_p} - \frac{b}{11} < p < \frac{c_t}{2c_p} - \frac{b}{11}$ |
| Max pure contributors ($C$): | $N - \left\lceil \frac{N \min(k_{in})}{\max(k_{out})} \right\rceil = 12 - \left\lceil \frac{24}{2} \right\rceil = 0$ | |
| Graph Efficiency ($E$): | $E = 1 - \frac{N \min(k_{in}) - N + 1}{(N-1)^2} = 0.8926$ | |
| Minimum Graph Hierarchy: | $\frac{2N-2C}{2N-C-1} = 0/11 = 0.0000$ | |
| Graph hierarchy score distribution from 1,000,000 random samples: | | |
| 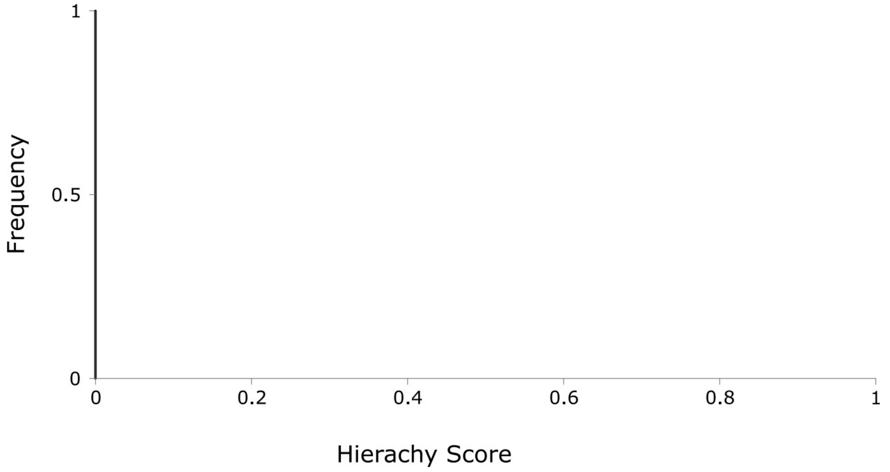 | | |
| Low H score 0.0000 | Median H score 0.0000 | High H score 0.0000 |
| | 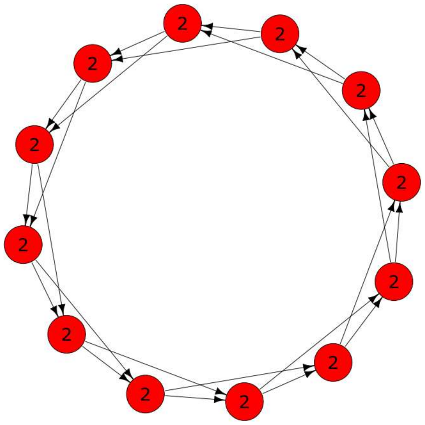 | |



Table S14: Properties of socially efficient networks when $\min(k_{in}) = 2$, $\max(k_{out}) = 2$, universal threshold contribution is a Nash equilibrium, and pure contributors are maximized.

| | | |
|---|---|---|
| $N = 12$ | $\min(k_{in}) = 2$ | $\max(k_{out}) = 3$ |
| Parameter range: | $\frac{4}{c_t - c_p} < m < \frac{4}{c_t - c_p}$ | $\frac{c_t}{c_p} - \frac{b}{11} < p < \frac{c_t}{2c_p} - \frac{b}{11}$ |
| Max pure contributors ($C$): | $N - \left\lceil \frac{N \min(k_{in})}{\max(k_{out})} \right\rceil = 12 - \left\lceil \frac{24}{3} \right\rceil = 4$ | |
| Graph Efficiency ($E$): | $E = 1 - \frac{N \min(k_{in}) - N + 1}{(N-1)^2} = 0.8926$ | |
| Minimum Graph Hierarchy: | $\frac{2N-2C}{2N-C-1} = 8/15 = 0.5333$ | |

Graph hierarchy score distribution from 1,000,000 random samples:

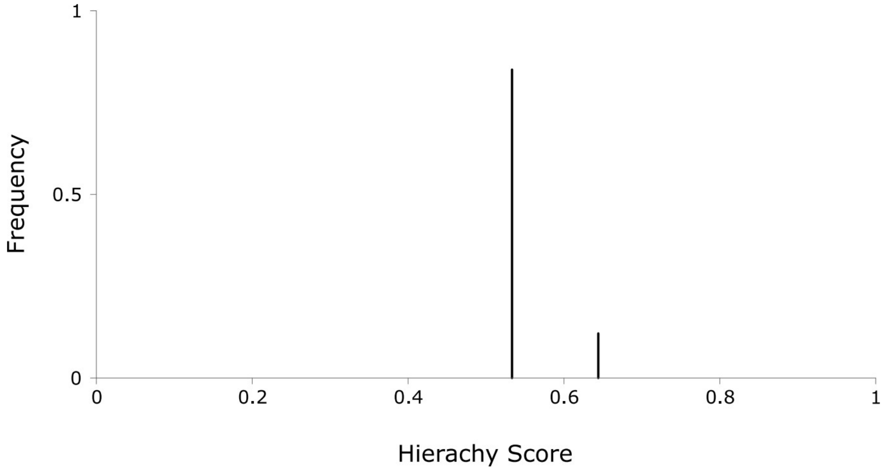


| Low H score | Median H score | High H score* |
|---|---|---|
| 0.5333 | 0.5333 | 0.9492 |

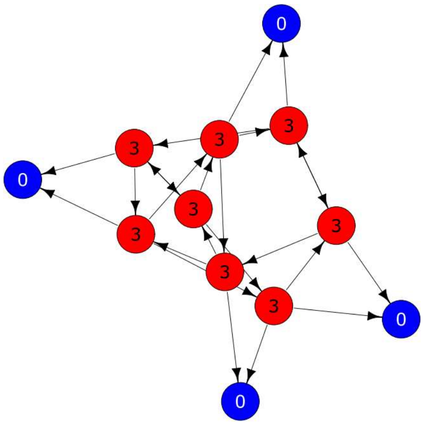

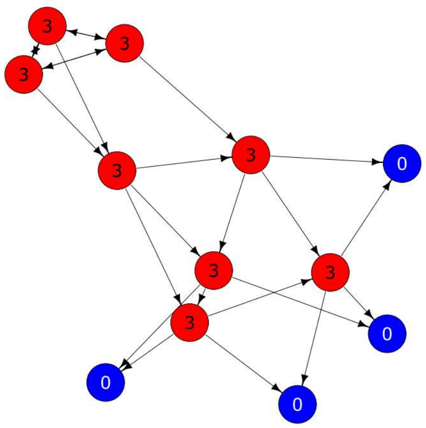

Table S15: Properties of socially efficient networks when $\min(k_{in}) = 2$, $\max(k_{out}) = 3$, universal threshold contribution is a Nash equilibrium, and pure contributors are maximized. The high $H$ score was found with a targeted search.

| $N = 12$ | $\min(k_{in}) = 2$ | $\max(k_{out}) = 4$ |
|---|---|---|
| Parameter range: | $\frac{4}{c_t - c_p} < m < \frac{4}{c_t - c_p}$ | $\frac{c_t}{c_p} - \frac{b}{11} < p < \frac{c_t}{2c_p} - \frac{b}{11}$ |
| Max pure contributors ($C$): | $N - \left\lceil \frac{N\min(k_{in})}{\max(k_{out})} \right\rceil = 12 - \left\lceil \frac{24}{4} \right\rceil = 6$ | |
| Graph Efficiency ($E$): | $E = 1 - \frac{N\min(k_{in}) - N + 1}{(N-1)^2} = 0.8926$ | |
| Minimum Graph Hierarchy: | $\frac{2N-2C}{2N-C-1} = 12/17 = 0.7059$ | |

Graph hierarchy score distribution from 1,000,000 random samples:

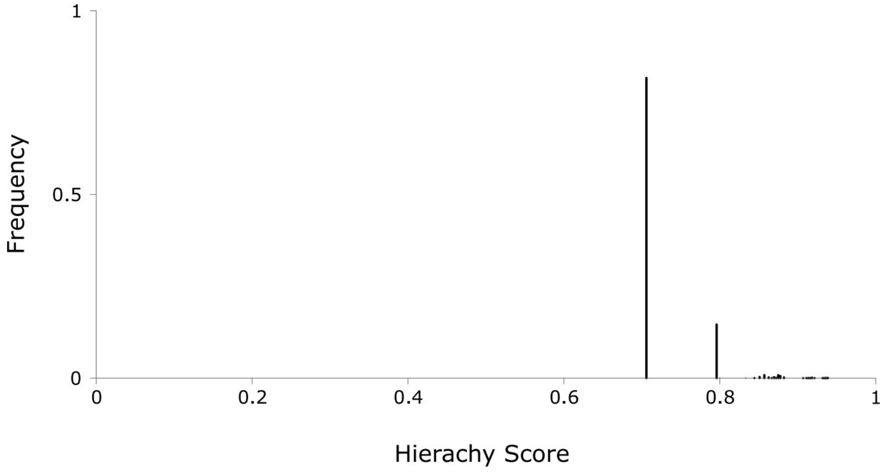


| Low H score | Median H score | High H score |
|---|---|---|
| 0.7059 | 0.7059 | 0.9388 |

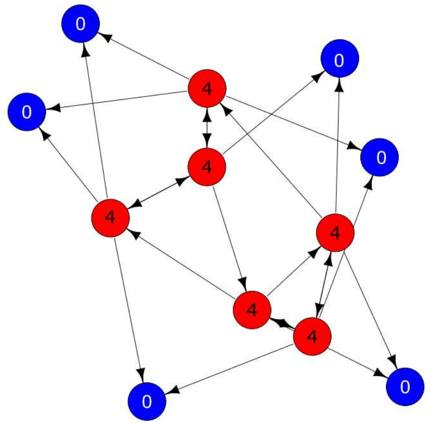

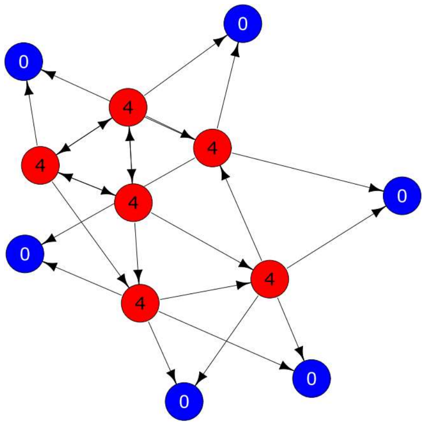

Table S16: Properties of socially efficient networks when $\min(k_{in}) = 2$, $\max(k_{out}) = 4$, universal threshold contribution is a Nash equilibrium, and pure contributors are maximized.

| $N = 12$ | $\min(k_{in}) = 2$ | $\max(k_{out}) = 5$ |
|---|---|---|
| Parameter range: | $\frac{4}{c_t - c_p} < m < \frac{4}{c_t - c_p}$ | $\frac{c_t}{c_p} - \frac{b}{11} < p < \frac{c_t}{2c_p} - \frac{b}{11}$ |
| Max pure contributors ($C$): | $N - \left\lceil \frac{N\min(k_{in})}{\max(k_{out})} \right\rceil = 12 - \left\lceil \frac{24}{5} \right\rceil = 7$ | |
| Graph Efficiency ($E$): | $E = 1 - \frac{N\min(k_{in}) - N + 1}{(N-1)^2} = 0.8926$ | |
| Minimum Graph Hierarchy: | $\frac{2N - 2C}{2N - C - 1} = 14/18 = 0.7778$ | |

Graph hierarchy score distribution from 1,000,000 random samples:

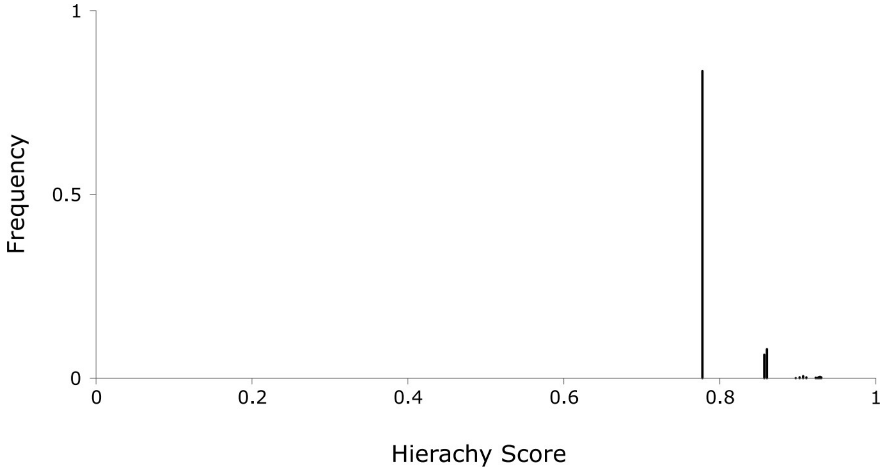


| Low H score | Median H score | High H score |
|---|---|---|
| 0.7778 | 0.7778 | 0.9302 |

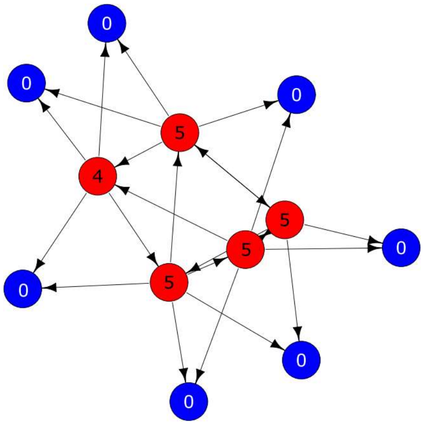


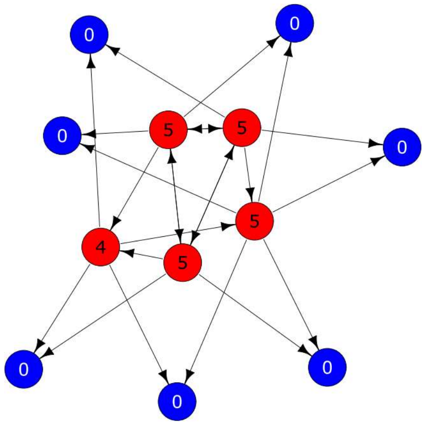


Table S17: Properties of socially efficient networks when $\min(k_{in}) = 2$, $\max(k_{out}) = 5$, universal threshold contribution is a Nash equilibrium, and pure contributors are maximized.

| | | |
|---|---|---|
| $N = 12$ | $\min(k_{in}) = 2$ | $\max(k_{out}) = 6$ |
| Parameter range: | $\frac{4}{c_t - c_p} < m < \frac{4}{c_t - c_p}$ | $\frac{c_t}{c_p} - \frac{b}{11} < p < \frac{c_t}{2c_p} - \frac{b}{11}$ |
| Max pure contributors ($C$): | $N - \left\lceil \frac{N \min(k_{in})}{\max(k_{out})} \right\rceil = 12 - \left\lceil \frac{24}{6} \right\rceil = 8$ | |
| Graph Efficiency ($E$): | $E = 1 - \frac{N \min(k_{in}) - N + 1}{(N-1)^2} = 0.8926$ | |
| Minimum Graph Hierarchy: | $\frac{2N - 2C}{2N - C - 1} = 16/19 = 0.8421$ | |

Graph hierarchy score distribution from 1,000,000 random samples:

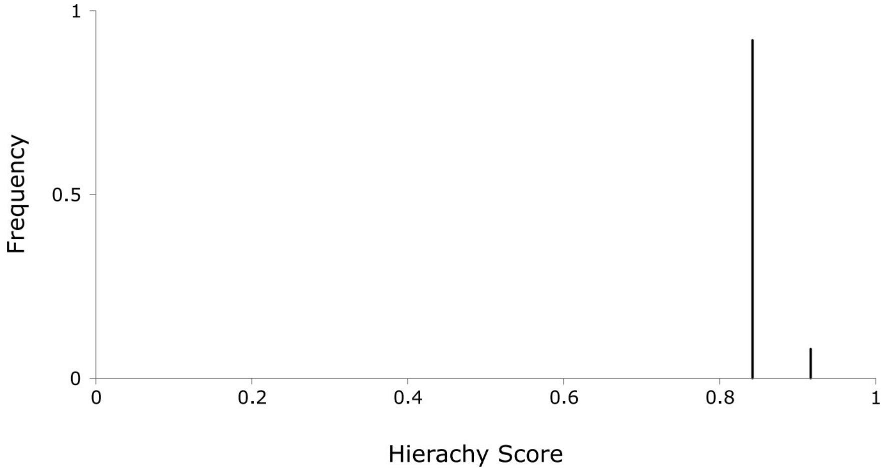


| Low H score | Median H score | High H score |
|---|---|---|
| 0.8421 | 0.8421 | 0.9167 |

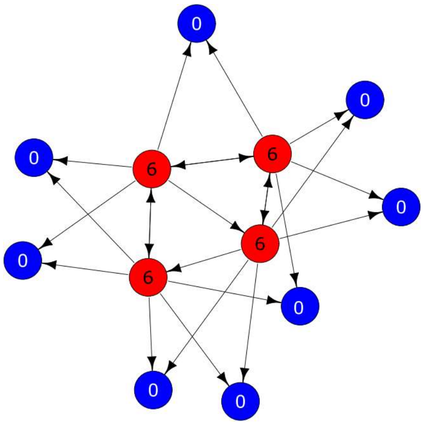

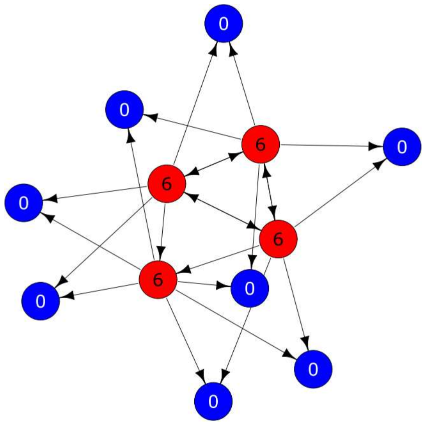

Table S18: Properties of socially efficient networks when $\min(k_{in}) = 2$, $\max(k_{out}) = 6$, universal threshold contribution is a Nash equilibrium, and pure contributors are maximized.

| | | |
|---|---|---|
| $N = 12$ | $\min(k_{in}) = 2$ | $\max(k_{out}) = 7$ |
| Parameter range: | $\frac{4}{c_t - c_p} < m < \frac{4}{c_t - c_p}$ | $\frac{c_t}{c_p} - \frac{b}{11} < p < \frac{c_t}{2c_p} - \frac{b}{11}$ |
| Max pure contributors ($C$): | $N - \left\lceil \frac{N \min(k_{in})}{\max(k_{out})} \right\rceil = 12 - \left\lceil \frac{24}{7} \right\rceil = 8$ | |
| Graph Efficiency ($E$): | $E = 1 - \frac{N \min(k_{in}) - N + 1}{(N-1)^2} = 0.8926$ | |
| Minimum Graph Hierarchy: | $\frac{2N-2C}{2N-C-1} = 18/20 = 0.9000$ | |

Graph hierarchy score distribution from 1,000,000 random samples:

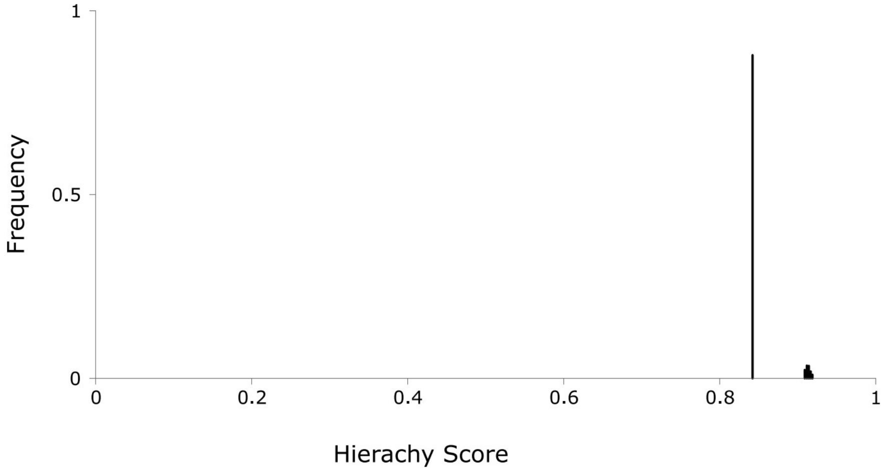


| Low H score | Median H score | High H score |
|---|---|---|
| 0.8421 | 0.8421 | 0.9189 |

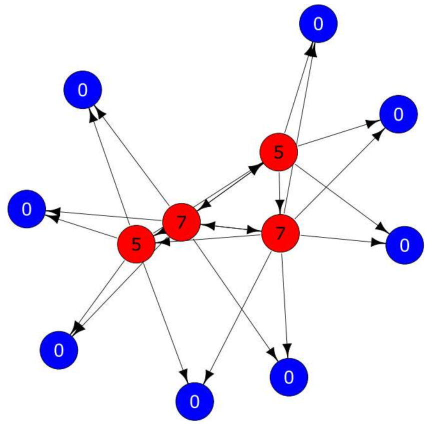


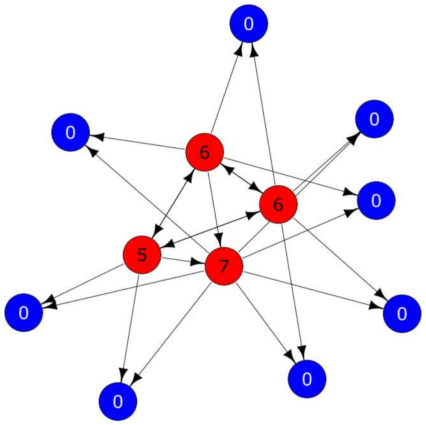


Table S19: Properties of socially efficient networks when $\min(k_{in}) = 2$, $\max(k_{out}) = 7$, universal threshold contribution is a Nash equilibrium, and pure contributors are maximized.

| | | |
|---|---|---|
| $N = 12$ | $\min(k_{in}) = 2$ | $\max(k_{out}) = 8$ |
| Parameter range: | $\frac{4}{c_t - c_p} < m < \frac{4}{c_t - c_p}$ | $\frac{c_t}{c_p} - \frac{b}{11} < p < \frac{c_t}{2c_p} - \frac{b}{11}$ |
| Max pure contributors ($C$): | $N - \left\lceil \frac{N\min(k_{in})}{\max(k_{out})} \right\rceil = 12 - \left\lceil \frac{24}{8} \right\rceil = 9$ | |
| Graph Efficiency ($E$): | $E = 1 - \frac{N\min(k_{in}) - N + 1}{(N-1)^2} = 0.8926$ | |
| Minimum Graph Hierarchy: | $\frac{2N-2C}{2N-C-1} = 18/20 = 0.9000$ | |

Graph hierarchy score distribution from 1,000,000 random samples:

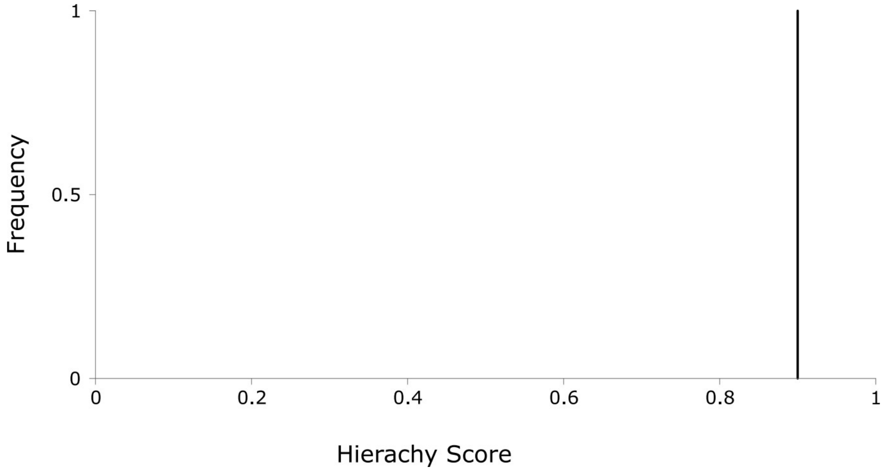


| Low H score | Median H score | High H score |
|---|---|---|
| 0.9000 | 0.9000 | 0.9000 |

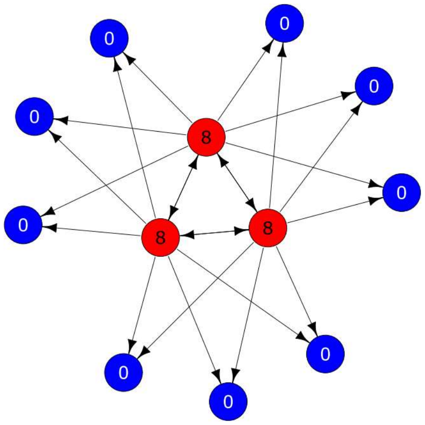


Table S20: Properties of socially efficient networks when $\min(k_{in}) = 2$, $\max(k_{out}) = 8$, universal threshold contribution is a Nash equilibrium, and pure contributors are maximized.

| | | |
|---|---|---|
| $N = 12$ | $\min(k_{in}) = 2$ | $\max(k_{out}) = 9$ |
| Parameter range: | $\frac{4}{c_t - c_p} < m < \frac{4}{c_t - c_p}$ | $\frac{c_t}{c_p} - \frac{b}{11} < p < \frac{c_t}{2c_p} - \frac{b}{11}$ |
| Max pure contributors ($C$): | $N - \left\lceil \frac{N \min(k_{in})}{\max(k_{out})} \right\rceil = 12 - \left\lceil \frac{24}{9} \right\rceil = 9$ | |
| Graph Efficiency ($E$): | $E = 1 - \frac{N \min(k_{in}) - N + 1}{(N-1)^2} = 0.8926$ | |
| Minimum Graph Hierarchy: | $\frac{2N-2C}{2N-C-1} = 18/20 = 0.9000$ | |

Graph hierarchy score distribution from 1,000,000 random samples:

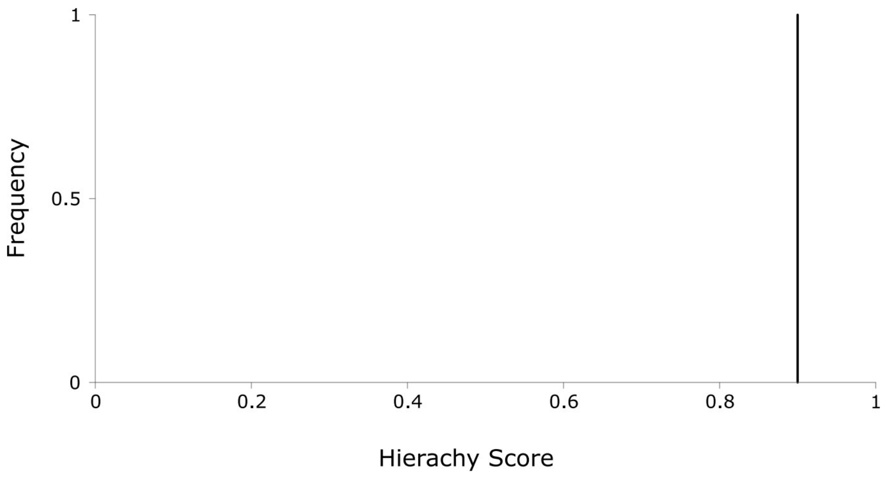


| Low H score | Median H score | High H score |
|---|---|---|
| 0.9000 | 0.9000 | 0.9000 |

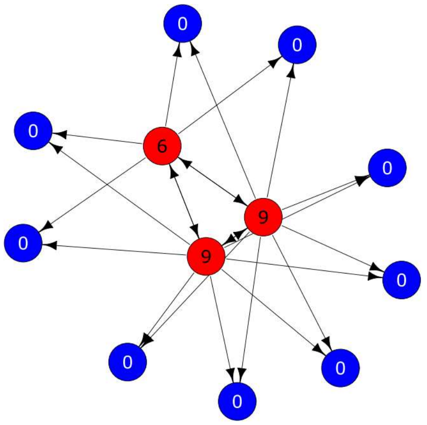

Table S21: Properties of socially efficient networks when $\min(k_{in}) = 2$, $\max(k_{out}) = 9$, universal threshold contribution is a Nash equilibrium, and pure contributors are maximized.

| | | |
|---|---|---|
| $N = 12$ | $\min(k_{in}) = 2$ | $\max(k_{out}) = 10$ |
| Parameter range: | $\frac{4}{c_t - c_p} < m < \frac{4}{c_t - c_p}$ | $\frac{c_t}{c_p} - \frac{b}{11} < p < \frac{c_t}{2c_p} - \frac{b}{11}$ |
| Max pure contributors ($C$): | $N - \left\lceil \frac{N \min(k_{in})}{\max(k_{out})} \right\rceil = 12 - \left\lceil \frac{24}{10} \right\rceil = 9$ | |
| Graph Efficiency ($E$): | $E = 1 - \frac{N \min(k_{in}) - N + 1}{(N-1)^2} = 0.8926$ | |
| Minimum Graph Hierarchy: | $\frac{2N - 2C}{2N - C - 1} = 18/20 = 0.9000$ | |

Graph hierarchy score distribution from 1,000,000 random samples:

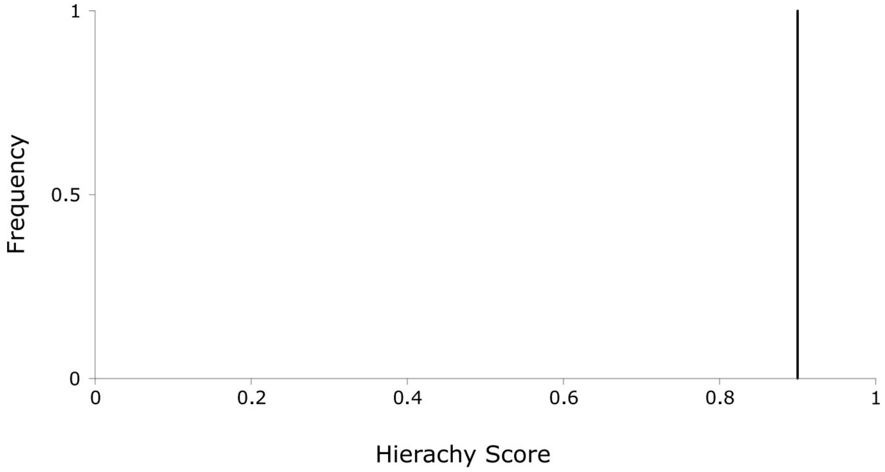


| Low H score | Median H score | High H score |
|---|---|---|
| 0.9000 | 0.9000 | 0.9000 |

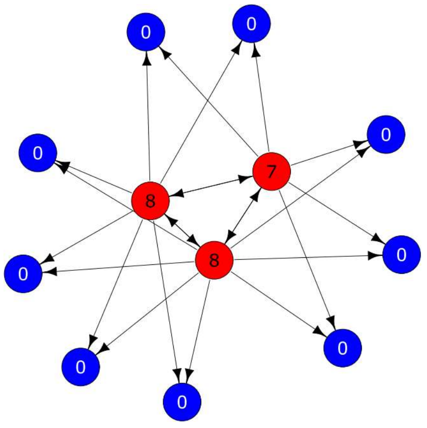

Table S22: Properties of socially efficient networks when $\min(k_{in}) = 2$, $\max(k_{out}) = 10$, universal threshold contribution is a Nash equilibrium, and pure contributors are maximized.

| | | |
|---|---|---|
| $N = 12$ | $\min(k_{in}) = 2$ | $\max(k_{out}) = 11$ |
| Parameter range: | $\frac{4}{c_t - c_p} < m < \frac{4}{c_t - c_p}$ | $\frac{c_t}{c_p} - \frac{b}{11} < p < \frac{c_t}{2c_p} - \frac{b}{11}$ |
| Max pure contributors ($C$): | $N - \left\lceil \frac{N\min(k_{in})}{\max(k_{out})} \right\rceil = 12 - \left\lceil \frac{24}{11} \right\rceil = 9$ | |
| Graph Efficiency ($E$): | $E = 1 - \frac{N\min(k_{in}) - N + 1}{(N-1)^2} = 0.8926$ | |
| Minimum Graph Hierarchy: | $\frac{2N-2C}{2N-C-1} = 18/20 = 0.9000$ | |

Graph hierarchy score distribution from 1,000,000 random samples:

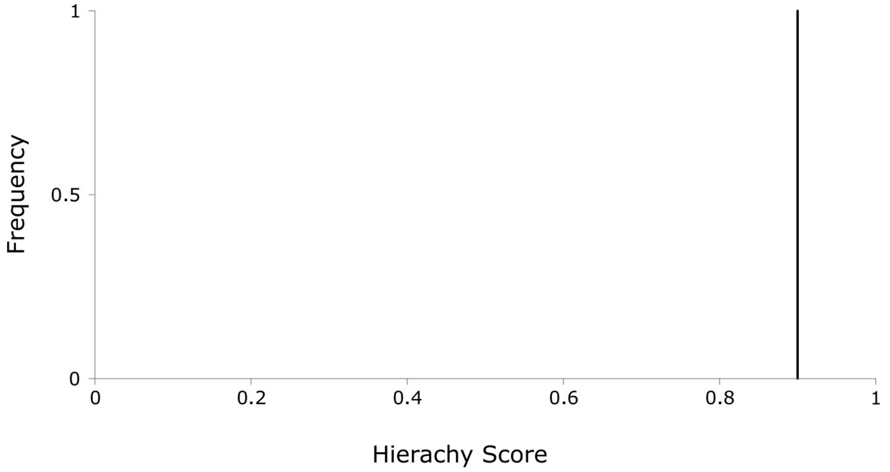


| Low H score | Median H score | High H score |
|---|---|---|
| 0.9000 | 0.9000 | 0.9000 |

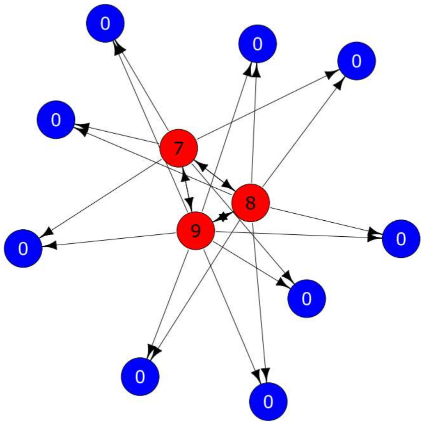


Table S23: Properties of socially efficient networks when $\min(k_{in}) = 2$, $\max(k_{out}) = 11$, universal threshold contribution is a Nash equilibrium, and pure contributors are maximized.

| | | |
|---|---|---|
| $N = 12$ | $\min(k_{in}) = 3$ | $\max(k_{out}) = 3$ |
| Parameter range: | $\frac{4}{c_t - c_p} < m < \frac{4}{c_t - c_p}$ | $\frac{c_t}{c_p} - \frac{b}{11} < p < \frac{c_t}{2c_p} - \frac{b}{11}$ |
| Max pure contributors ($C$): | $N - \left\lceil \frac{N \min(k_{in})}{\max(k_{out})} \right\rceil = 12 - \left\lceil \frac{36}{3} \right\rceil = 0$ | |
| Graph Efficiency ($E$): | $E = 1 - \frac{N \min(k_{in}) - N + 1}{(N-1)^2} = 0.7934$ | |
| Minimum Graph Hierarchy: | $\frac{2N-2C}{2N-C-1} = 0/11 = 0.0000$ | |
| Graph hierarchy score distribution from 1,000,000 random samples: 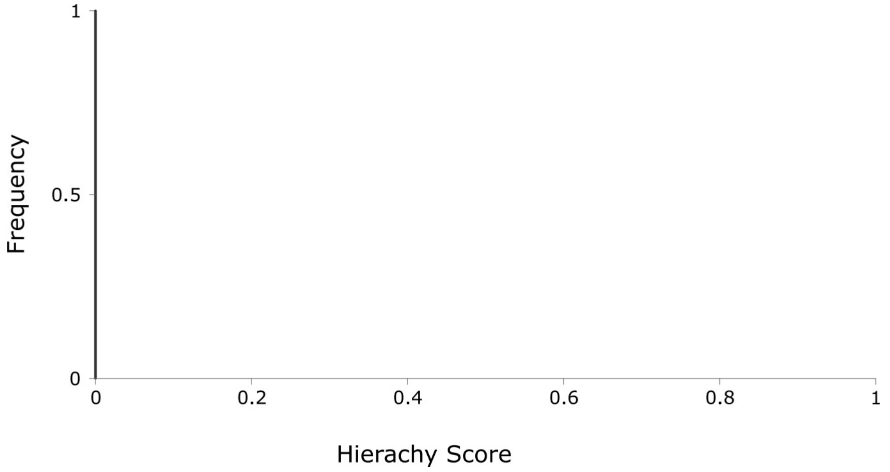  | | |
| Low H score<br>0.0000 | Median H score<br>0.0000 | High H score<br>0.0000 |
| | 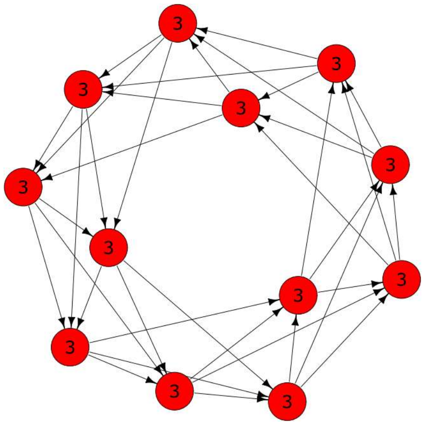  | |

Table S24: Properties of socially efficient networks when $\min(k_{in}) = 3$, $\max(k_{out}) = 3$, universal threshold contribution is a Nash equilibrium, and pure contributors are maximized.

| | | |
|---|---|---|
| $N = 12$ | $\min(k_{in}) = 3$ | $\max(k_{out}) = 4$ |
| Parameter range: | $\frac{4}{c_t - c_p} < m < \frac{4}{c_t - c_p}$ | $\frac{c_t}{c_p} - \frac{b}{11} < p < \frac{c_t}{2c_p} - \frac{b}{11}$ |
| Max pure contributors ($C$): | $N - \left\lceil \frac{N \min(k_{in})}{\max(k_{out})} \right\rceil = 12 - \left\lceil \frac{36}{4} \right\rceil = 3$ | |
| Graph Efficiency ($E$): | $E = 1 - \frac{N \min(k_{in}) - N + 1}{(N-1)^2} = 0.7934$ | |
| Minimum Graph Hierarchy: | $\frac{2N-2C}{2N-C-1} = 86/14 = 0.4286$ | |
| Graph hierarchy score distribution from 1,000,000 random samples: 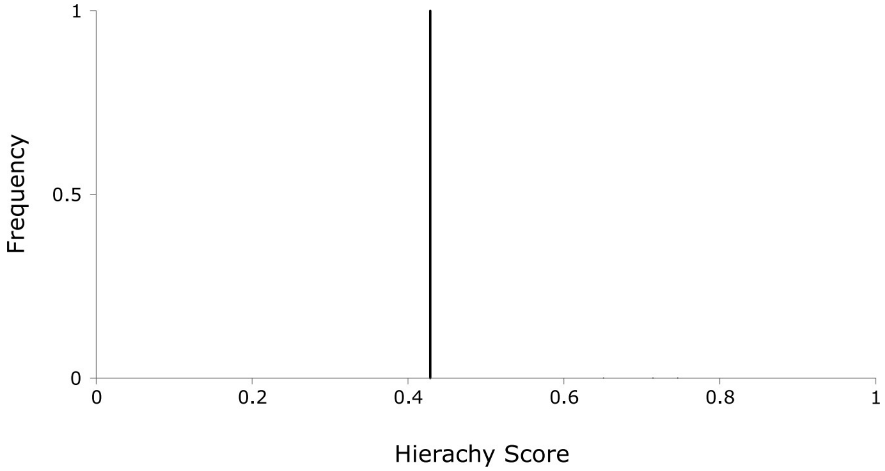  | | |
| Low H score 0.4286 | Median H score 0.4286 | High H score* 0.8413 |
| 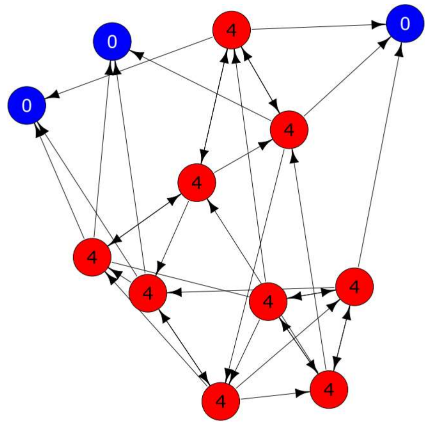 | | 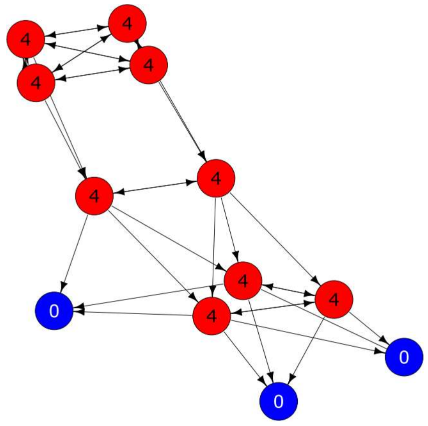 |

Table S25: Properties of socially efficient networks when $\min(k_{in}) = 3$, $\max(k_{out}) = 4$, universal threshold contribution is a Nash equilibrium, and pure contributors are maximized. The high $H$ score was found with a targeted search.

| | | |
|---|---|---|
| $N = 12$ | $\min(k_{in}) = 3$ | $\max(k_{out}) = 5$ |
| Parameter range: | $\frac{4}{c_t - c_p} < m < \frac{4}{c_t - c_p}$ | $\frac{c_t}{c_p} - \frac{b}{11} < p < \frac{c_t}{2c_p} - \frac{b}{11}$ |
| Max pure contributors ($C$): | $N - \left\lceil \frac{N \min(k_{in})}{\max(k_{out})} \right\rceil = 12 - \left\lceil \frac{36}{5} \right\rceil = 4$ | |
| Graph Efficiency ($E$): | $E = 1 - \frac{N \min(k_{in}) - N + 1}{(N-1)^2} = 0.7934$ | |
| Minimum Graph Hierarchy: | $\frac{2N-2C}{2N-C-1} = 8/15 = 0.5333$ | |

Graph hierarchy score distribution from 1,000,000 random samples:

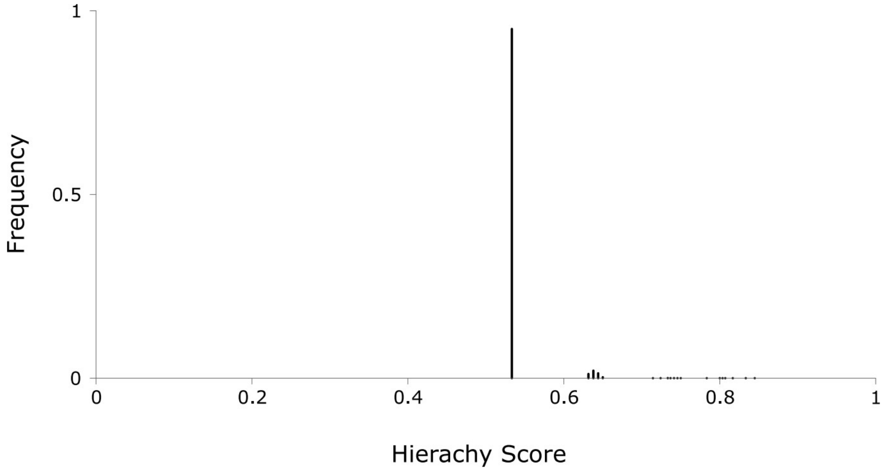


| Low H score | Median H score | High H score |
|---|---|---|
| 0.5333 | 0.5333 | 0.9000 |

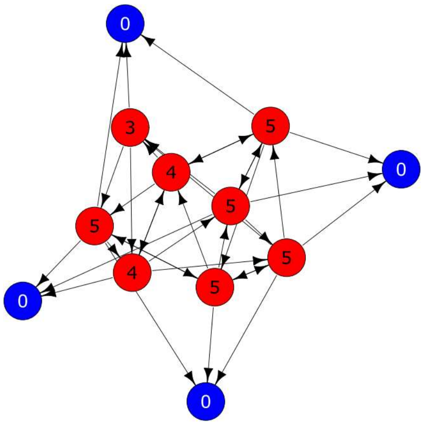


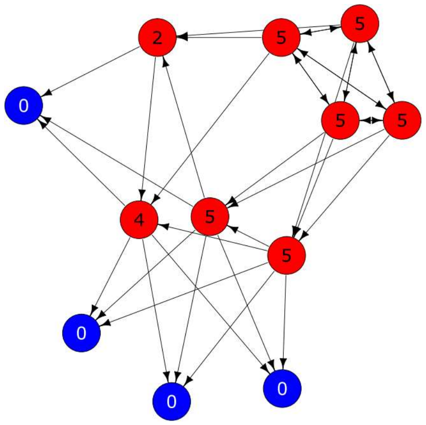


Table S26: Properties of socially efficient networks when $\min(k_{in}) = 3$, $\max(k_{out}) = 5$, universal threshold contribution is a Nash equilibrium, and pure contributors are maximized.

<table>
<tr><td>$N = 12$</td><td>$\min(k_{in}) = 3$</td><td>$\max(k_{out}) = 6$</td></tr>
<tr><td>Parameter range:</td><td>$\frac{4}{c_t - c_p} < m < \frac{4}{c_t - c_p}$</td><td>$\frac{c_t}{c_p} - \frac{b}{11} < p < \frac{c_t}{2c_p} - \frac{b}{11}$</td></tr>
<tr><td>Max pure contributors ($C$):</td><td colspan="2">$N - \left\lceil \frac{N \min(k_{in})}{\max(k_{out})} \right\rceil = 12 - \left\lceil \frac{36}{6} \right\rceil = 6$</td></tr>
<tr><td>Graph Efficiency ($E$):</td><td colspan="2">$E = 1 - \frac{N \min(k_{in}) - N + 1}{(N-1)^2} = 0.7934$</td></tr>
<tr><td>Minimum Graph Hierarchy:</td><td colspan="2">$\frac{2N - 2C}{2N - C - 1} = 12/17 = 0.7059$</td></tr>
<tr><td colspan="3">Graph hierarchy score distribution from 1,000,000 random samples:<br>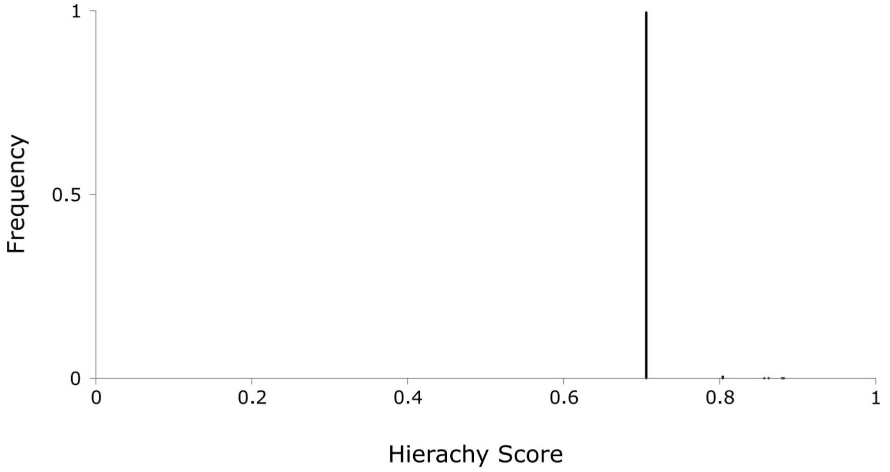<br></td></tr>
<tr><td colspan="2">Low H score 0.7059     Median H score 0.7059<br>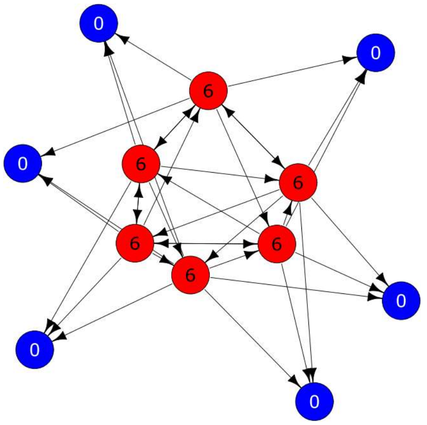</td><td>High H score 0.8824<br>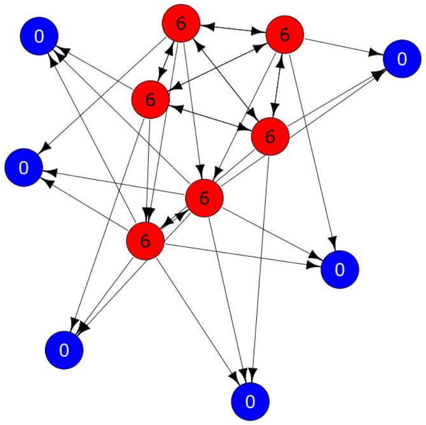</td></tr>
</table>

Table S27: Properties of socially efficient networks when $\min(k_{in}) = 3$, $\max(k_{out}) = 6$, universal threshold contribution is a Nash equilibrium, and pure contributors are maximized.

| $N = 12$ | $\min(k_{in}) = 3$ | $\max(k_{out}) = 7$ |
|---|---|---|
| Parameter range: | $\frac{4}{c_t - c_p} < m < \frac{4}{c_t - c_p}$ | $\frac{c_t}{c_p} - \frac{b}{11} < p < \frac{c_t}{2c_p} - \frac{b}{11}$ |
| Max pure contributors ($C$): | $N - \left\lceil \frac{N \min(k_{in})}{\max(k_{out})} \right\rceil = 12 - \left\lceil \frac{36}{7} \right\rceil = 6$ | |
| Graph Efficiency ($E$): | $E = 1 - \frac{N \min(k_{in}) - N + 1}{(N-1)^2} = 0.7934$ | |
| Minimum Graph Hierarchy: | $\frac{2N - 2C}{2N - C - 1} = 12/17 = 0.7059$ | |
| Graph hierarchy score distribution from 1,000,000 random samples: | | |
| Low H score 0.7059 | Median H score 0.7059 | High H score 0.8824 |

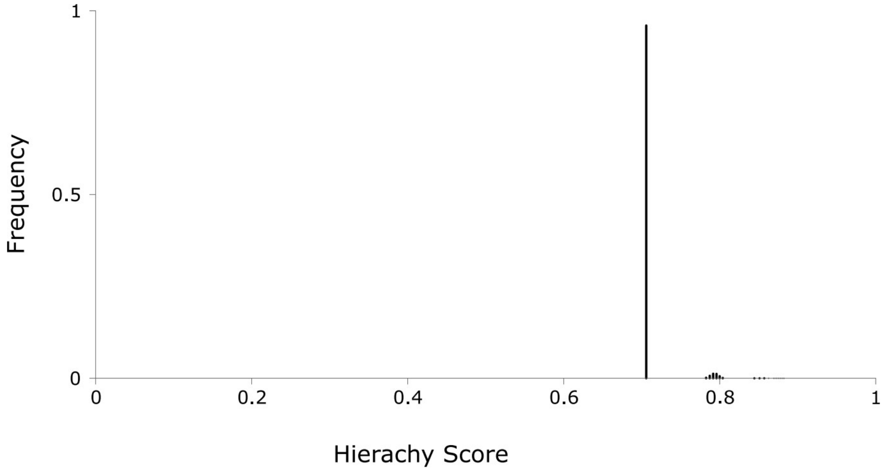


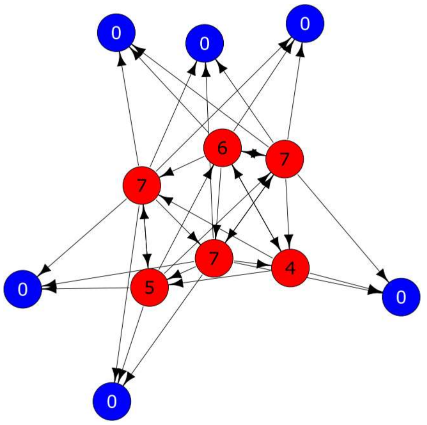

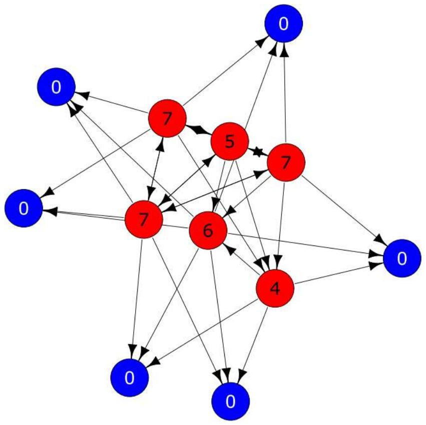

Table S28: Properties of socially efficient networks when $\min(k_{in}) = 3$, $\max(k_{out}) = 7$, universal threshold contribution is a Nash equilibrium, and pure contributors are maximized.

| | | |
|---|---|---|
| $N = 12$ | $\min(k_{in}) = 3$ | $\max(k_{out}) = 8$ |
| Parameter range: | $\frac{4}{c_t - c_p} < m < \frac{4}{c_t - c_p}$ | $\frac{c_t}{c_p} - \frac{b}{11} < p < \frac{c_t}{2c_p} - \frac{b}{11}$ |
| Max pure contributors ($C$): | $N - \left\lceil \frac{N\min(k_{in})}{\max(k_{out})} \right\rceil = 12 - \left\lceil \frac{36}{8} \right\rceil = 7$ | |
| Graph Efficiency ($E$): | $E = 1 - \frac{N\min(k_{in}) - N + 1}{(N-1)^2} = 0.7934$ | |
| Minimum Graph Hierarchy: | $\frac{2N-2C}{2N-C-1} = 14/18 = 0.7778$ | |

Graph hierarchy score distribution from 1,000,000 random samples:

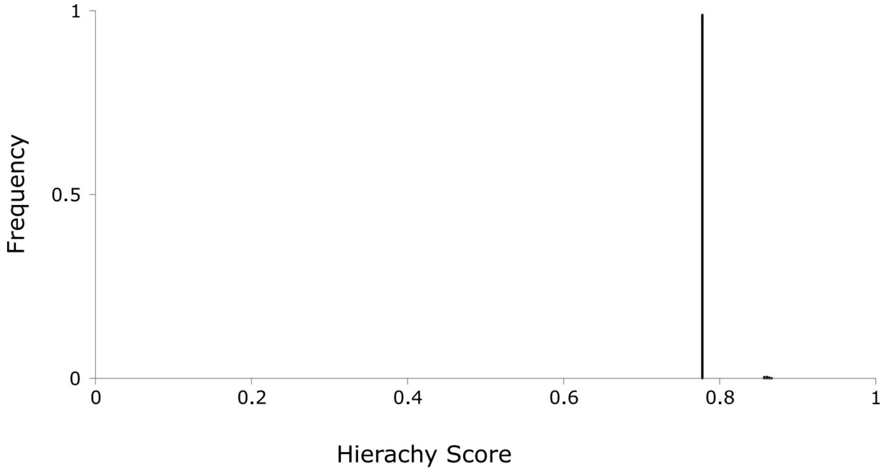


| Low H score | Median H score | High H score |
|---|---|---|
| 0.7778 | 0.7778 | 0.8667 |

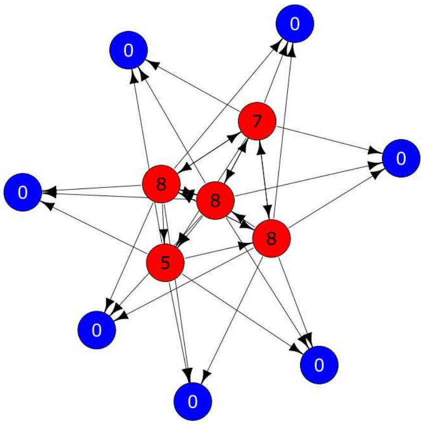


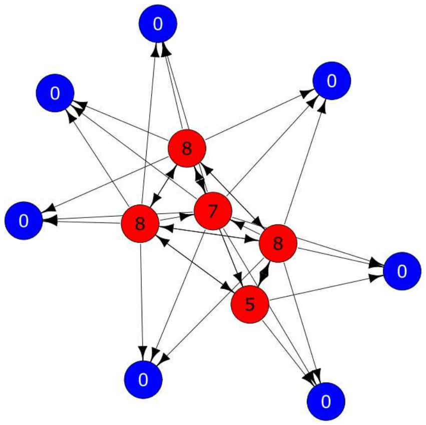


Table S29: Properties of socially efficient networks when $\min(k_{in}) = 3$, $\max(k_{out}) = 8$, universal threshold contribution is a Nash equilibrium, and pure contributors are maximized.

| | | |
|---|---|---|
| $N = 12$ | $\min(k_{in}) = 3$ | $\max(k_{out}) = 9$ |
| Parameter range: | $\frac{4}{c_t - c_p} < m < \frac{4}{c_t - c_p}$ | $\frac{c_t}{c_p} - \frac{b}{11} < p < \frac{c_t}{2c_p} - \frac{b}{11}$ |
| Max pure contributors ($C$): | $N - \left\lceil \frac{N\min(k_{in})}{\max(k_{out})} \right\rceil = 12 - \left\lceil \frac{36}{9} \right\rceil = 8$ | |
| Graph Efficiency ($E$): | $E = 1 - \frac{N\min(k_{in}) - N + 1}{(N-1)^2} = 0.7934$ | |
| Minimum Graph Hierarchy: | $\frac{2N-2C}{2N-C-1} = 16/19 = 0.8421$ | |

Graph hierarchy score distribution from 1,000,000 random samples:

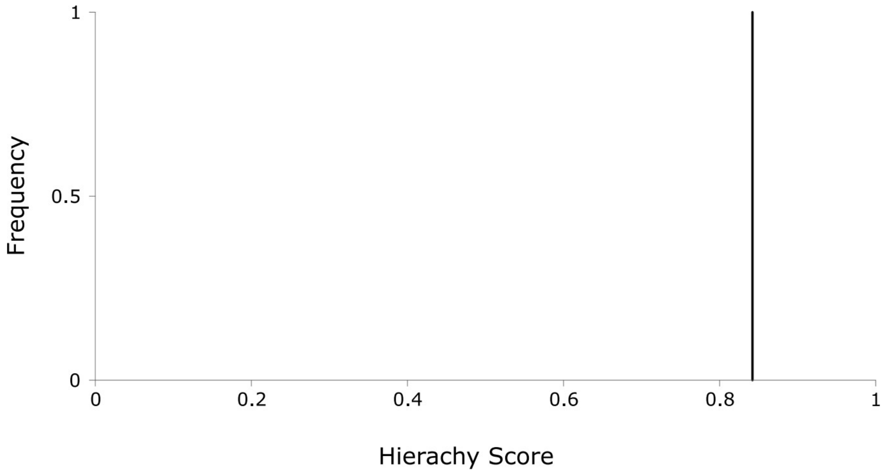


| Low H score | Median H score | High H score |
|---|---|---|
| 0.8421 | 0.8421 | 0.8421 |

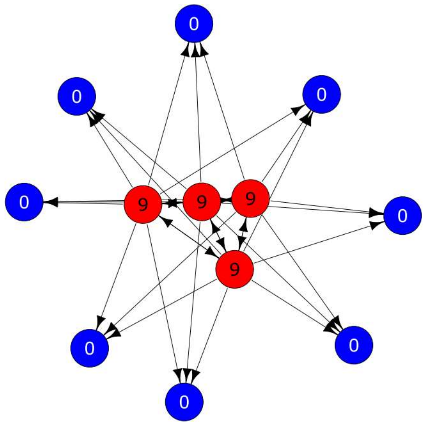


Table S30: Properties of socially efficient networks when $\min(k_{in}) = 3$, $\max(k_{out}) = 9$, universal threshold contribution is a Nash equilibrium, and pure contributors are maximized.

| | | |
|---|---|---|
| $N = 12$ | $\min(k_{in}) = 3$ | $\max(k_{out}) = 10$ |
| Parameter range: | $\frac{4}{c_t - c_p} < m < \frac{4}{c_t - c_p}$ | $\frac{c_t}{c_p} - \frac{b}{11} < p < \frac{c_t}{2c_p} - \frac{b}{11}$ |
| Max pure contributors ($C$): | $N - \left\lceil \frac{N \min(k_{in})}{\max(k_{out})} \right\rceil = 12 - \left\lceil \frac{36}{10} \right\rceil = 8$ | |
| Graph Efficiency ($E$): | $E = 1 - \frac{N \min(k_{in}) - N + 1}{(N-1)^2} = 0.7934$ | |
| Minimum Graph Hierarchy: | $\frac{2N-2C}{2N-C-1} = 16/19 = 0.8421$ | |

Graph hierarchy score distribution from 1,000,000 random samples:

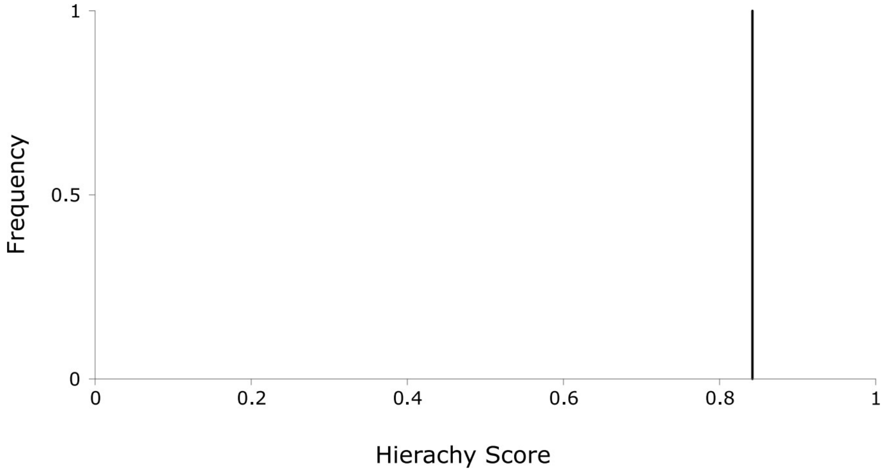


| Low H score | Median H score | High H score |
|---|---|---|
| 0.8421 | 0.8421 | 0.8421 |

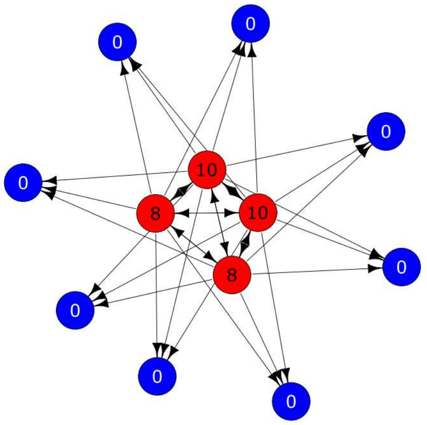


Table S31: Properties of socially efficient networks when $\min(k_{in}) = 3$, $\max(k_{out}) = 10$, universal threshold contribution is a Nash equilibrium, and pure contributors are maximized.

| $N = 12$ | $\min(k_{in}) = 3$ | $\max(k_{out}) = 11$ |
|---|---|---|
| Parameter range: | $\frac{4}{c_t - c_p} < m < \frac{4}{c_t - c_p}$ | $\frac{c_t}{c_p} - \frac{b}{11} < p < \frac{c_t}{2c_p} - \frac{b}{11}$ |
| Max pure contributors ($C$): | $N - \left\lceil \frac{N\min(k_{in})}{\max(k_{out})} \right\rceil = 12 - \left\lceil \frac{36}{11} \right\rceil = 8$ | |
| Graph Efficiency ($E$): | $E = 1 - \frac{N\min(k_{in}) - N + 1}{(N-1)^2} = 0.7934$ | |
| Minimum Graph Hierarchy: | $\frac{2N-2C}{2N-C-1} = 16/19 = 0.8421$ | |

Graph hierarchy score distribution from 1,000,000 random samples:

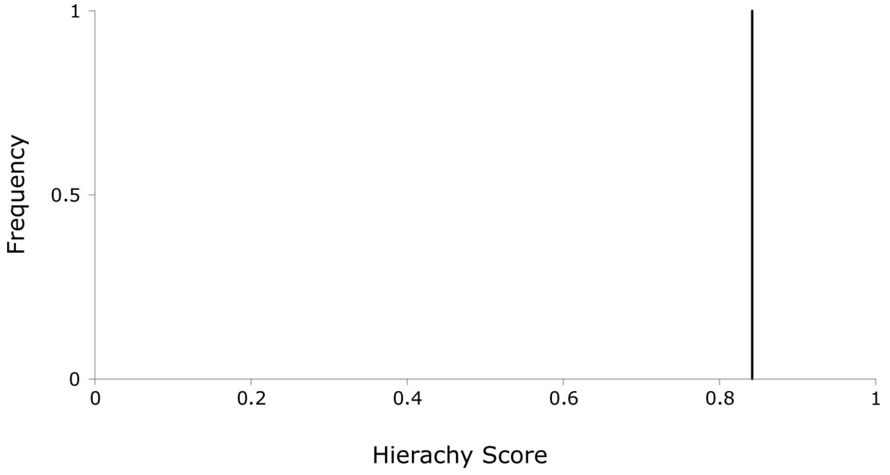


| Low H score | Median H score | High H score |
|---|---|---|
| 0.8421 | 0.8421 | 0.8421 |

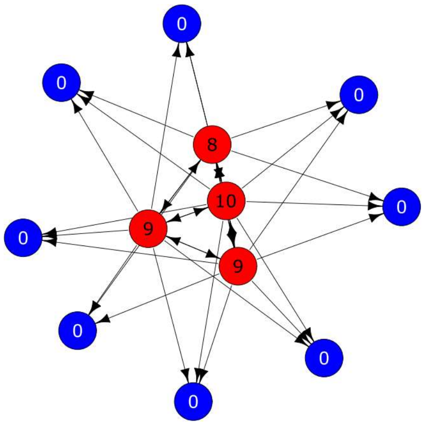


Table S32: Properties of socially efficient networks when $\min(k_{in}) = 3$, $\max(k_{out}) = 11$, universal threshold contribution is a Nash equilibrium, and pure contributors are maximized.

| $N = 12$ | $\min(k_{in}) = 4$ | $\max(k_{out}) = 4$ |
|---|---|---|
| Parameter range: | $\frac{4}{c_t-c_p} < m < \frac{4}{c_t-c_p}$ | $\frac{c_t}{c_p} - \frac{b}{11} < p < \frac{c_t}{2c_p} - \frac{b}{11}$ |
| Max pure contributors ($C$): | $N - \left\lceil \frac{N \min(k_{in})}{\max(k_{out})} \right\rceil = 12 - \left\lceil \frac{48}{4} \right\rceil = 0$ | |
| Graph Efficiency ($E$): | $E = 1 - \frac{N \min(k_{in}) - N + 1}{(N-1)^2} = 0.76942$ | |
| Minimum Graph Hierarchy: | $\frac{2N-2C}{2N-C-1} = 0/11 = 0.0000$ | |
| Graph hierarchy score distribution from 1,000,000 random samples: 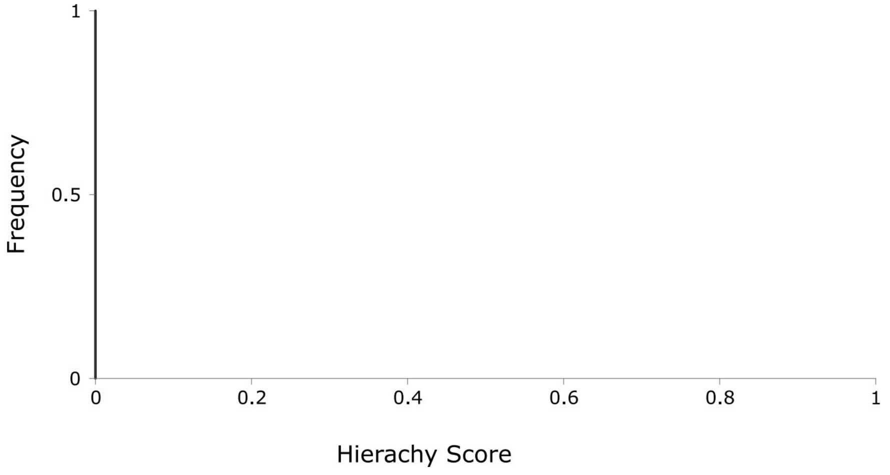  | | |
| Low H score 0.0000 | Median H score 0.0000 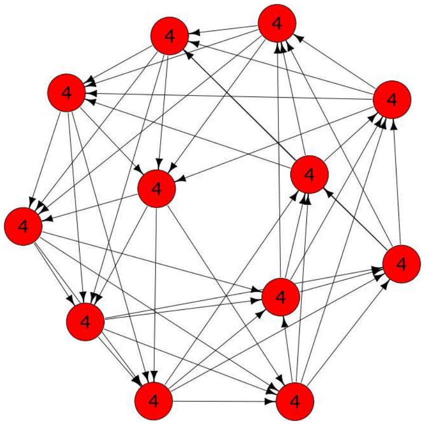 | High H score 0.0000 |

Table S33: Properties of socially efficient networks when $\min(k_{in}) = 4$, $\max(k_{out}) = 4$, universal threshold contribution is a Nash equilibrium, and pure contributors are maximized.

| $N = 12$ | $\min(k_{in}) = 4$ | $\max(k_{out}) = 5$ |
|---|---|---|
| Parameter range: | $\frac{4}{c_t - c_p} < m < \frac{4}{c_t - c_p}$ | $\frac{c_t}{c_p} - \frac{b}{11} < p < \frac{c_t}{2c_p} - \frac{b}{11}$ |
| Max pure contributors ($C$): | $N - \left\lceil \frac{N \min(k_{in})}{\max(k_{out})} \right\rceil = 12 - \left\lceil \frac{48}{5} \right\rceil = 2$ | |
| Graph Efficiency ($E$): | $E = 1 - \frac{N \min(k_{in}) - N + 1}{(N-1)^2} = 0.76942$ | |
| Minimum Graph Hierarchy: | $\frac{2N-2C}{2N-C-1} = 4/13 = 0.3077$ | |

Graph hierarchy score distribution from 1,000,000 random samples:

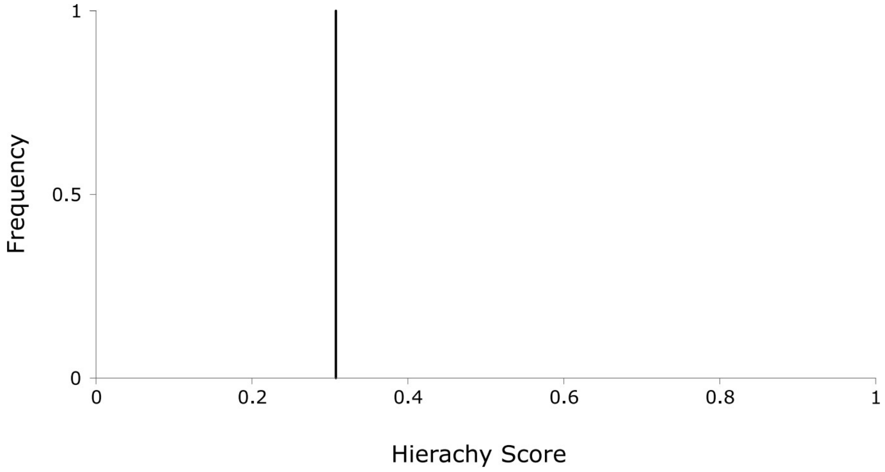


| Low H score | Median H score | High H score* |
|---|---|---|
| 0.3077 | 0.3077 | 0.7538 |

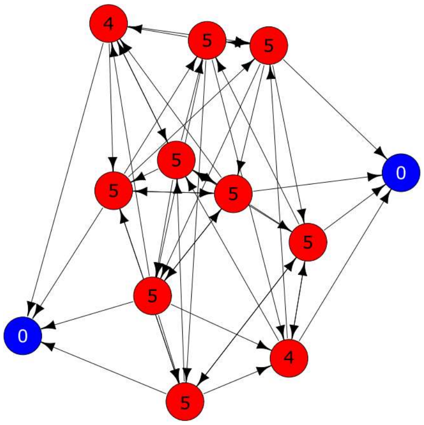

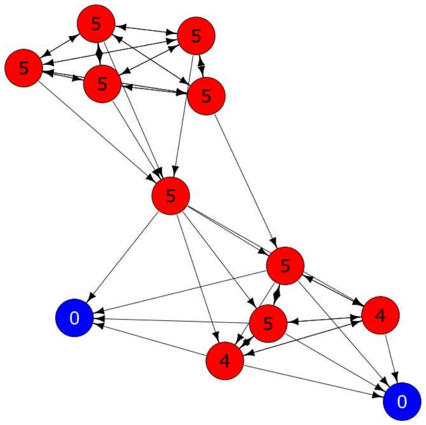

Table S34: Properties of socially efficient networks when $\min(k_{in}) = 4$, $\max(k_{out}) = 5$, universal threshold contribution is a Nash equilibrium, and pure contributors are maximized.

| $N = 12$ | $\min(k_{in}) = 4$ | $\max(k_{out}) = 6$ |
|---|---|---|
| Parameter range: | $\frac{4}{c_t - c_p} < m < \frac{4}{c_t - c_p}$ | $\frac{c_t}{c_p} - \frac{b}{11} < p < \frac{c_t}{2c_p} - \frac{b}{11}$ |
| Max pure contributors ($C$): | $N - \left\lceil \frac{N \min(k_{in})}{\max(k_{out})} \right\rceil = 12 - \left\lceil \frac{48}{6} \right\rceil = 4$ | |
| Graph Efficiency ($E$): | $E = 1 - \frac{N \min(k_{in}) - N + 1}{(N-1)^2} = 0.76942$ | |
| Minimum Graph Hierarchy: | $\frac{2N-2C}{2N-C-1} = 8/15 = 0.5333$ | |

Graph hierarchy score distribution from 1,000,000 random samples:

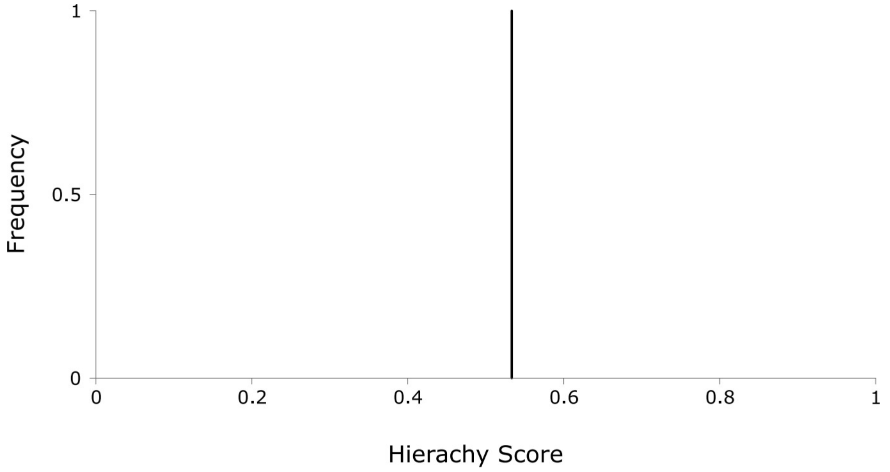


| Low H score | Median H score | High H score |
|---|---|---|
| 0.5333 | 0.5333 | 0.7833 |

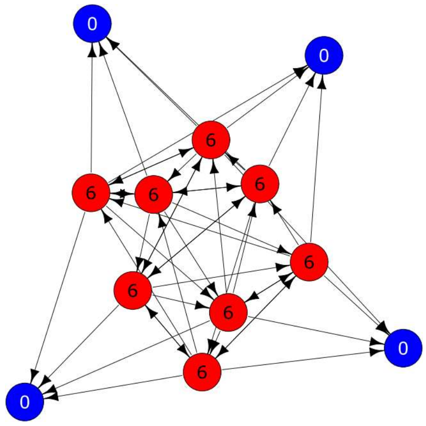

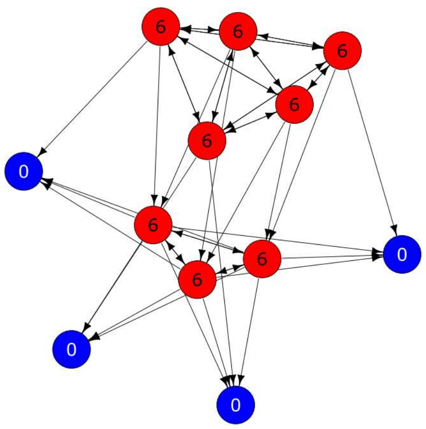

Table S35: Properties of socially efficient networks when $\min(k_{in}) = 4$, $\max(k_{out}) = 6$, universal threshold contribution is a Nash equilibrium, and pure contributors are maximized.

| | | |
|---|---|---|
| $N = 12$ | $\min(k_{in}) = 4$ | $\max(k_{out}) = 7$ |
| Parameter range: | $\frac{4}{c_t - c_p} < m < \frac{4}{c_t - c_p}$ | $\frac{c_t}{c_p} - \frac{b}{11} < p < \frac{c_t}{2c_p} - \frac{b}{11}$ |
| Max pure contributors ($C$): | $N - \left\lceil \frac{N \min(k_{in})}{\max(k_{out})} \right\rceil = 12 - \left\lceil \frac{48}{7} \right\rceil = 5$ | |
| Graph Efficiency ($E$): | $E = 1 - \frac{N \min(k_{in}) - N + 1}{(N-1)^2} = 0.76942$ | |
| Minimum Graph Hierarchy: | $\frac{2N-2C}{2N-C-1} = 10/16 = 0.6250$ | |

Graph hierarchy score distribution from 1,000,000 random samples:

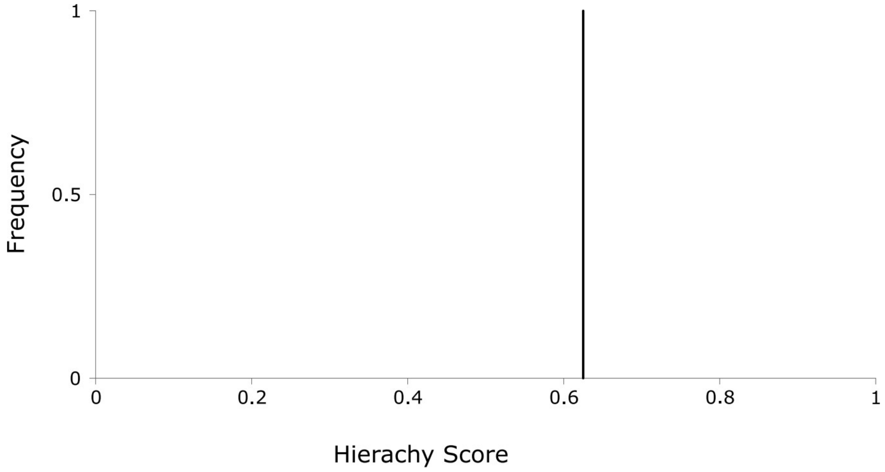


| Low H score | Median H score | High H score |
|---|---|---|
| 0.6250 | 0.6250 | 0.6250 |

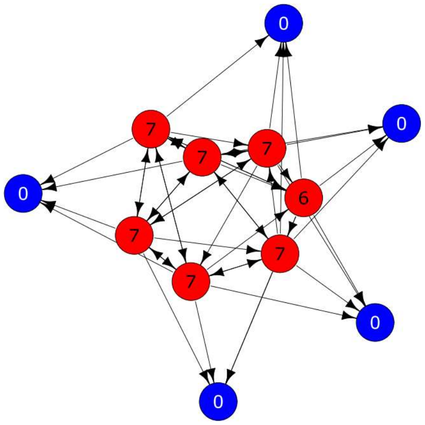


Table S36: Properties of socially efficient networks when $\min(k_{in}) = 4$, $\max(k_{out}) = 7$, universal threshold contribution is a Nash equilibrium, and pure contributors are maximized.

| | | |
|---|---|---|
| $N = 12$ | $\min(k_{in}) = 4$ | $\max(k_{out}) = 8$ |
| Parameter range: | $\frac{4}{c_t - c_p} < m < \frac{4}{c_t - c_p}$ | $\frac{c_t}{c_p} - \frac{b}{11} < p < \frac{c_t}{2c_p} - \frac{b}{11}$ |
| Max pure contributors ($C$): | $N - \left\lceil \frac{N\min(k_{in})}{\max(k_{out})} \right\rceil = 12 - \left\lceil \frac{48}{8} \right\rceil = 6$ | |
| Graph Efficiency ($E$): | $E = 1 - \frac{N\min(k_{in}) - N + 1}{(N-1)^2} = 0.76942$ | |
| Minimum Graph Hierarchy: | $\frac{2N-2C}{2N-C-1} = 12/17 = 0.7059$ | |

Graph hierarchy score distribution from 1,000,000 random samples:

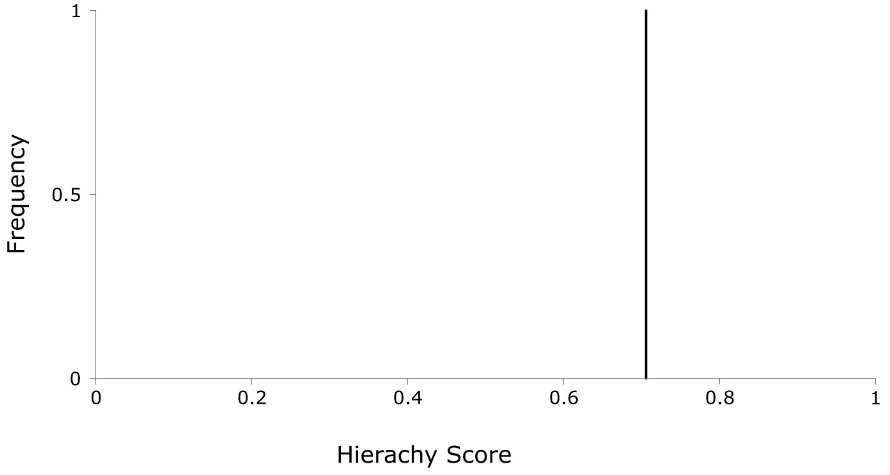


| Low H score | Median H score | High H score |
|---|---|---|
| 0.7059 | 0.7059 | 0.0759 |

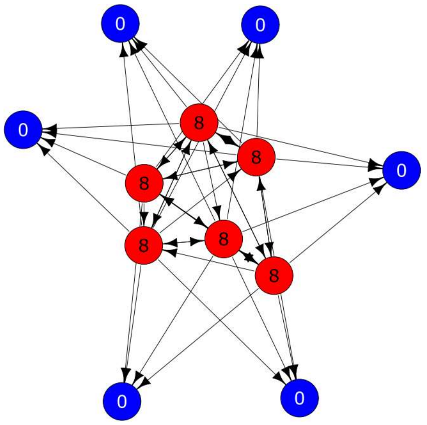


Table S37: Properties of socially efficient networks when $\min(k_{in}) = 4$, $\max(k_{out}) = 8$, universal threshold contribution is a Nash equilibrium, and pure contributors are maximized.

| | | |
|---|---|---|
| $N = 12$ | $\min(k_{in}) = 4$ | $\max(k_{out}) = 9$ |
| Parameter range: | $\frac{4}{c_t - c_p} < m < \frac{4}{c_t - c_p}$ | $\frac{c_t}{c_p} - \frac{b}{11} < p < \frac{c_t}{2c_p} - \frac{b}{11}$ |
| Max pure contributors ($C$): | $N - \left\lceil \frac{N \min(k_{in})}{\max(k_{out})} \right\rceil = 12 - \left\lceil \frac{48}{9} \right\rceil = 6$ | |
| Graph Efficiency ($E$): | $E = 1 - \frac{N \min(k_{in}) - N + 1}{(N-1)^2} = 0.76942$ | |
| Minimum Graph Hierarchy: | $\frac{2N - 2C}{2N - C - 1} = 12/17 = 0.7059$ | |

Graph hierarchy score distribution from 1,000,000 random samples:

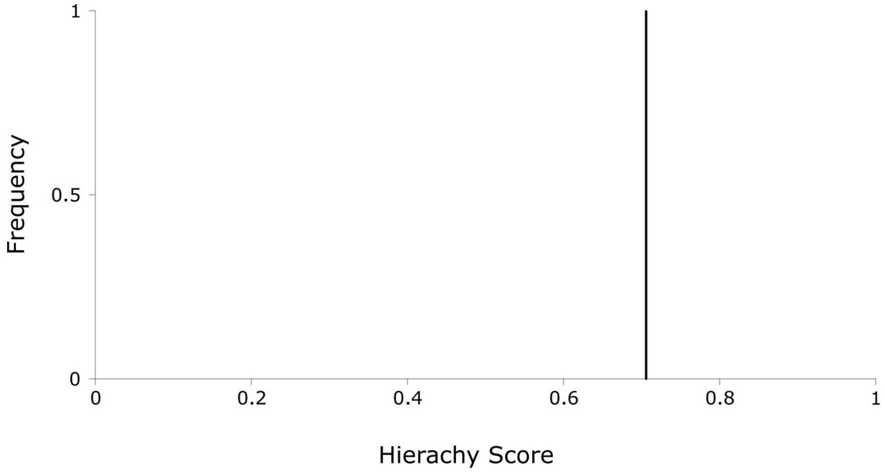


| Low H score | Median H score | High H score* |
|---|---|---|
| 0.7059 | 0.7059 | 0.8039 |

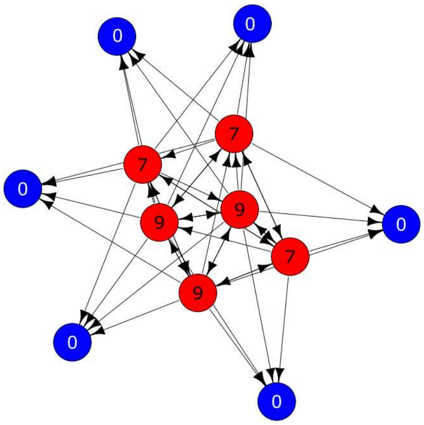

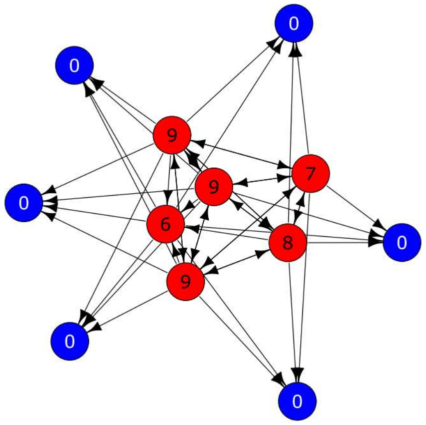

Table S38: Properties of socially efficient networks when $\min(k_{in}) = 4$, $\max(k_{out}) = 9$, universal threshold contribution is a Nash equilibrium, and pure contributors are maximized. The high $H$ score was found with a targeted search.

| $N = 12$ | $\min(k_{in}) = 4$ | $\max(k_{out}) = 10$ |
|---|---|---|
| Parameter range: | $\frac{4}{c_t - c_p} < m < \frac{4}{c_t - c_p}$ | $\frac{c_t}{c_p} - \frac{b}{11} < p < \frac{c_t}{2c_p} - \frac{b}{11}$ |
| Max pure contributors ($C$): | $N - \left\lceil \frac{N \min(k_{in})}{\max(k_{out})} \right\rceil = 12 - \left\lceil \frac{48}{10} \right\rceil = 7$ | |
| Graph Efficiency ($E$): | $E = 1 - \frac{N \min(k_{in}) - N + 1}{(N-1)^2} = 0.76942$ | |
| Minimum Graph Hierarchy: | $\frac{2N-2C}{2N-C-1} = 14/18 = 0.7778$ | |
| Graph hierarchy score distribution from 1,000,000 random samples: | | |

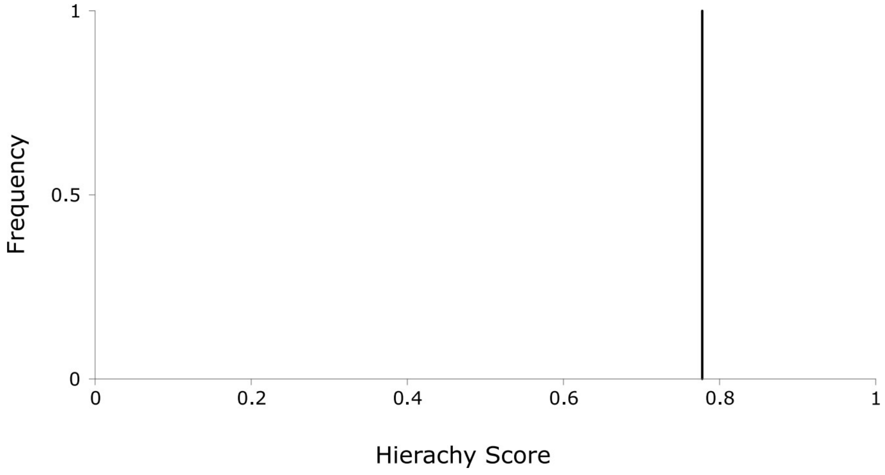


| Low H score | Median H score | High H score |
|---|---|---|
| 0.7778 | 0.7778 | 0.7778 |

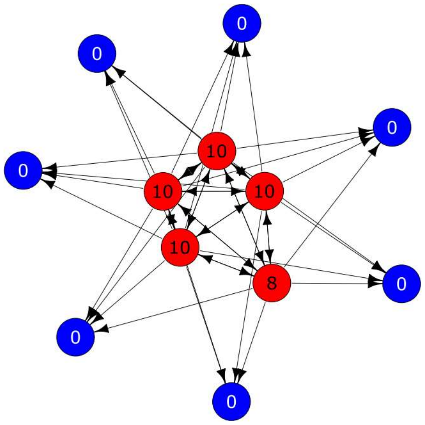


Table S39: Properties of socially efficient networks when $\min(k_{in}) = 4$, $\max(k_{out}) = 10$, universal threshold contribution is a Nash equilibrium, and pure contributors are maximized.

| $N = 12$ | $\min(k_{in}) = 4$ | $\max(k_{out}) = 11$ |
|---|---|---|
| Parameter range: | $\frac{4}{c_t - c_p} < m < \frac{4}{c_t - c_p}$ | $\frac{c_t}{c_p} - \frac{b}{11} < p < \frac{c_t}{2c_p} - \frac{b}{11}$ |
| Max pure contributors ($C$): | $N - \left\lceil \frac{N\min(k_{in})}{\max(k_{out})} \right\rceil = 12 - \left\lceil \frac{24}{11} \right\rceil = 7$ | |
| Graph Efficiency ($E$): | $E = 1 - \frac{N\min(k_{in}) - N + 1}{(N-1)^2} = 0.76942$ | |
| Minimum Graph Hierarchy: | $\frac{2N-2C}{2N-C-1} = 14/18 = 0.7778$ | |

Graph hierarchy score distribution from 1,000,000 random samples:

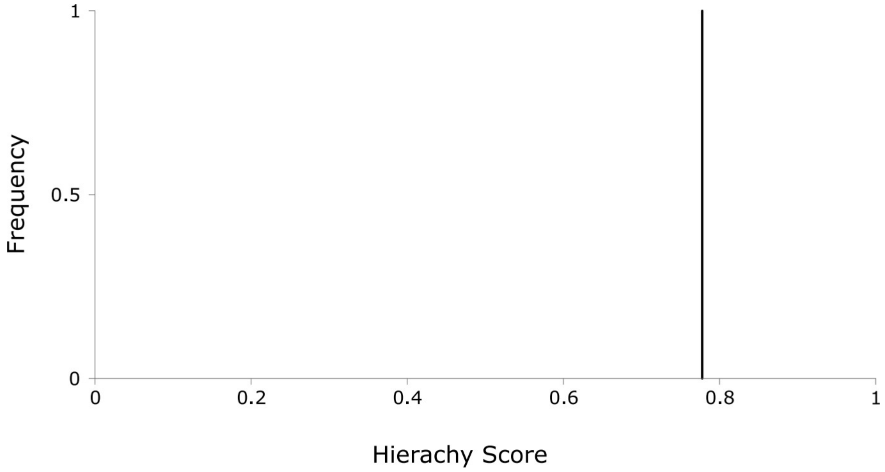


| Low H score | Median H score | High H score |
|---|---|---|
| 0.7778 | 0.7778 | 0.7778 |

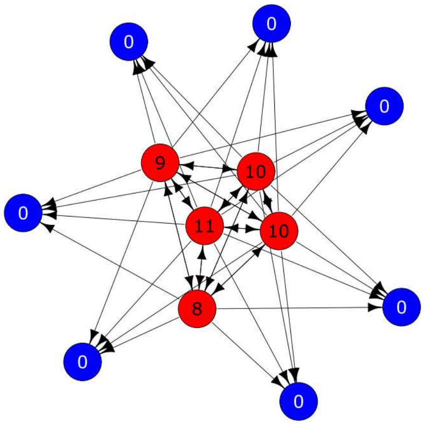


Table S40: Properties of socially efficient networks when $\min(k_{in}) = 4$, $\max(k_{out}) = 11$, universal threshold contribution is a Nash equilibrium, and pure contributors are maximized.

| | | |
|---|---|---|
| $N = 12$ | $\min(k_{in}) = 5$ | $\max(k_{out}) = 5$ |
| Parameter range: | $\frac{4}{c_t - c_p} < m < \frac{4}{c_t - c_p}$ | $\frac{c_t}{c_p} - \frac{b}{11} < p < \frac{c_t}{2c_p} - \frac{b}{11}$ |
| Max pure contributors ($C$): | $N - \left\lceil \frac{N \min(k_{in})}{\max(k_{out})} \right\rceil = 12 - \left\lceil \frac{60}{5} \right\rceil = 0$ | |
| Graph Efficiency ($E$): | $E = 1 - \frac{N \min(k_{in}) - N + 1}{(N-1)^2} = 0.5950$ | |
| Minimum Graph Hierarchy: | $\frac{2N-2C}{2N-C-1} = 0/11 = 0.0000$ | |

Graph hierarchy score distribution from 1,000,000 random samples:

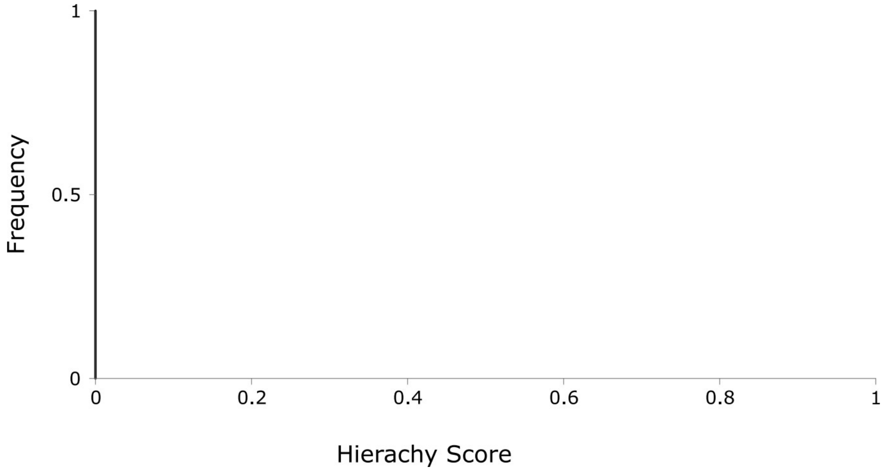


| Low H score | Median H score | High H score |
|---|---|---|
| 0.0000 | 0.0000 | 0.0000 |

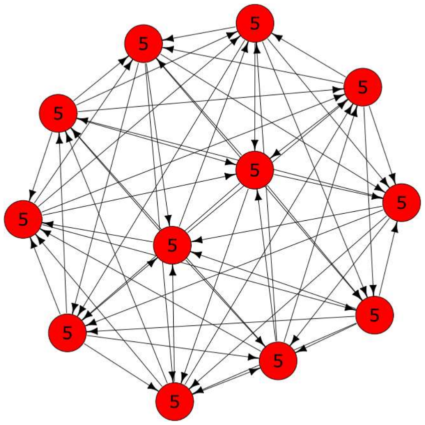


Table S41: Properties of socially efficient networks when $\min(k_{in}) = 5$, $\max(k_{out}) = 5$, universal threshold contribution is a Nash equilibrium, and pure contributors are maximized.

| | | |
|---|---|---|
| $N = 12$ | $\min(k_{in}) = 5$ | $\max(k_{out}) = 6$ |
| Parameter range: | $\frac{4}{c_t - c_p} < m < \frac{4}{c_t - c_p}$ | $\frac{c_t}{c_p} - \frac{b}{11} < p < \frac{c_t}{2c_p} - \frac{b}{11}$ |
| Max pure contributors ($C$): | $N - \left\lceil \frac{N \min(k_{in})}{\max(k_{out})} \right\rceil = 12 - \left\lceil \frac{60}{6} \right\rceil = 2$ | |
| Graph Efficiency ($E$): | $E = 1 - \frac{N \min(k_{in}) - N + 1}{(N-1)^2} = 0.5950$ | |
| Minimum Graph Hierarchy: | $\frac{2N-2C}{2N-C-1} = 4/13 = 0.3077$ | |

Graph hierarchy score distribution from 1,000,000 random samples:

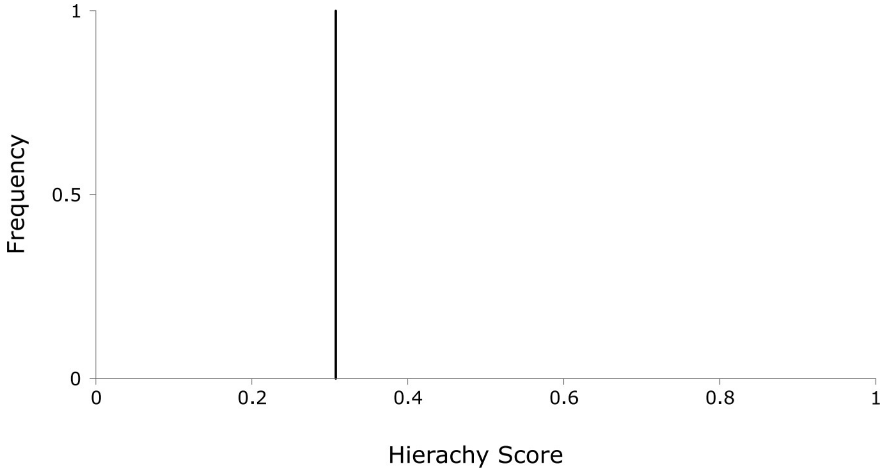


| Low H score | Median H score | High H score |
|---|---|---|
| 0.3077 | 0.3077 | 0.3077 |

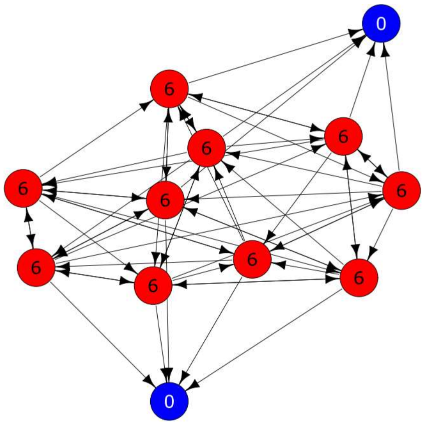


Table S42: Properties of socially efficient networks when $\min(k_{in}) = 5$, $\max(k_{out}) = 6$, universal threshold contribution is a Nash equilibrium, and pure contributors are maximized.

| | | |
|---|---|---|
| $N = 12$ | $\min(k_{in}) = 5$ | $\max(k_{out}) = 7$ |
| Parameter range: | $\frac{4}{c_t - c_p} < m < \frac{4}{c_t - c_p}$ | $\frac{c_t}{c_p} - \frac{b}{11} < p < \frac{c_t}{2c_p} - \frac{b}{11}$ |
| Max pure contributors ($C$): | $N - \left\lceil \frac{N\min(k_{in})}{\max(k_{out})} \right\rceil = 12 - \left\lceil \frac{60}{7} \right\rceil = 3$ | |
| Graph Efficiency ($E$): | $E = 1 - \frac{N\min(k_{in}) - N + 1}{(N-1)^2} = 0.5950$ | |
| Minimum Graph Hierarchy: | $\frac{2N-2C}{2N-C-1} = 6/14 = 0.4286$ | |

Graph hierarchy score distribution from 1,000,000 random samples:

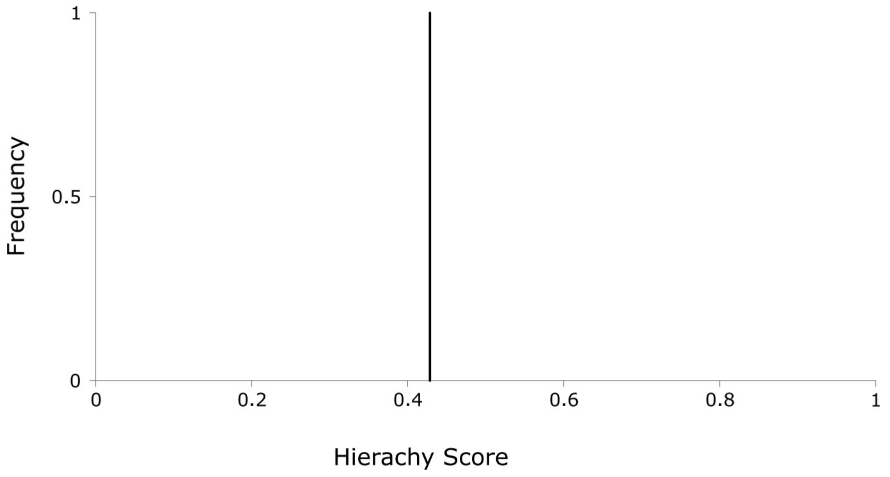


| Low H score | Median H score | High H score |
|---|---|---|
| 0.4286 | 0.4286 | 0.4286 |

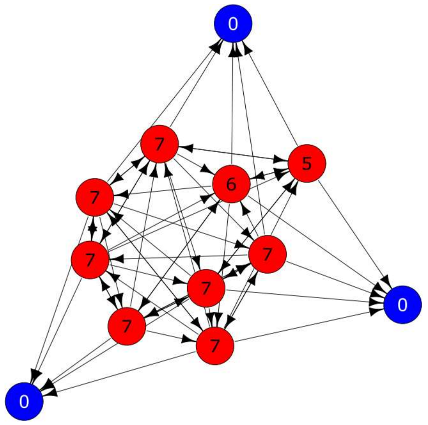


Table S43: Properties of socially efficient networks when $\min(k_{in}) = 5$, $\max(k_{out}) = 7$, universal threshold contribution is a Nash equilibrium, and pure contributors are maximized.

| $N = 12$ | $\min(k_{in}) = 5$ | $\max(k_{out}) = 8$ |
|---|---|---|
| Parameter range: | $\frac{4}{c_t - c_p} < m < \frac{4}{c_t - c_p}$ | $\frac{c_t}{c_p} - \frac{b}{11} < p < \frac{c_t}{2c_p} - \frac{b}{11}$ |
| Max pure contributors ($C$): | $N - \left\lceil \frac{N \min(k_{in})}{\max(k_{out})} \right\rceil = 12 - \left\lceil \frac{60}{8} \right\rceil = 4$ | |
| Graph Efficiency ($E$): | $E = 1 - \frac{N \min(k_{in}) - N + 1}{(N-1)^2} = 0.5950$ | |
| Minimum Graph Hierarchy: | $\frac{2N - 2C}{2N - C - 1} = 8/15 = 0.5333$ | |

Graph hierarchy score distribution from 1,000,000 random samples:

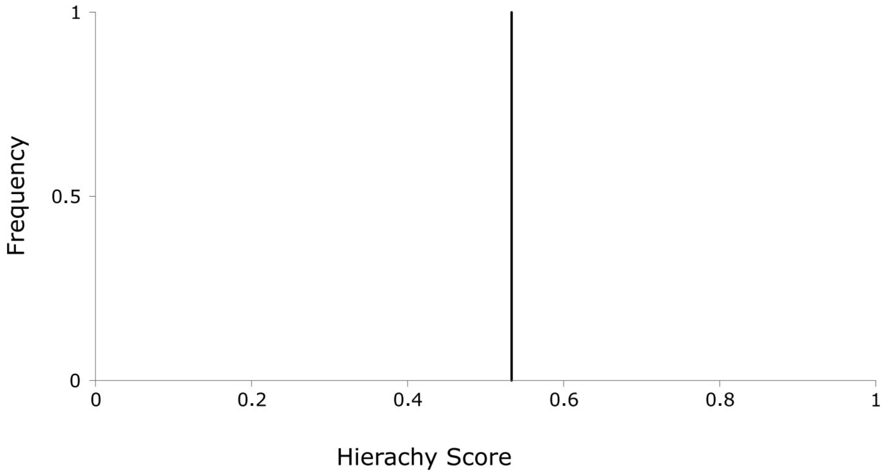


| Low H score | Median H score | High H score |
|---|---|---|
| 0.5333 | 0.5333 | 0.6500 |

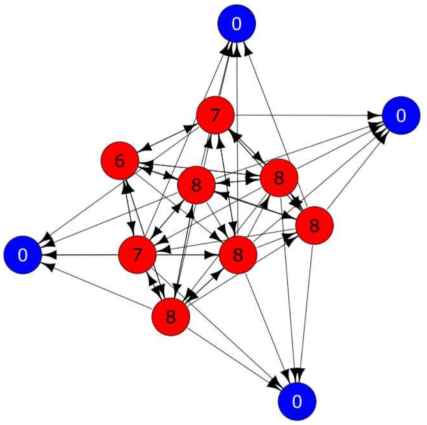


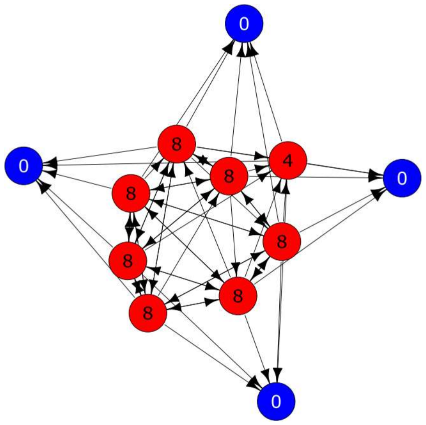


Table S44: Properties of socially efficient networks when $\min(k_{in}) = 5$, $\max(k_{out}) = 8$, universal threshold contribution is a Nash equilibrium, and pure contributors are maximized.

| $N = 12$ | $\min(k_{in}) = 5$ | $\max(k_{out}) = 9$ |
|---|---|---|
| Parameter range: | $\frac{4}{c_t-c_p} < m < \frac{4}{c_t-c_p}$ | $\frac{c_t}{c_p} - \frac{b}{11} < p < \frac{c_t}{2c_p} - \frac{b}{11}$ |
| Max pure contributors ($C$): | $N - \left\lceil \frac{N\min(k_{in})}{\max(k_{out})} \right\rceil = 12 - \left\lceil \frac{60}{9} \right\rceil = 5$ | |
| Graph Efficiency ($E$): | $E = 1 - \frac{N\min(k_{in})-N+1}{(N-1)^2} = 0.5950$ | |
| Minimum Graph Hierarchy: | $\frac{2N-2C}{2N-C-1} = 10/16 = 0.6250$ | |

Graph hierarchy score distribution from 1,000,000 random samples:

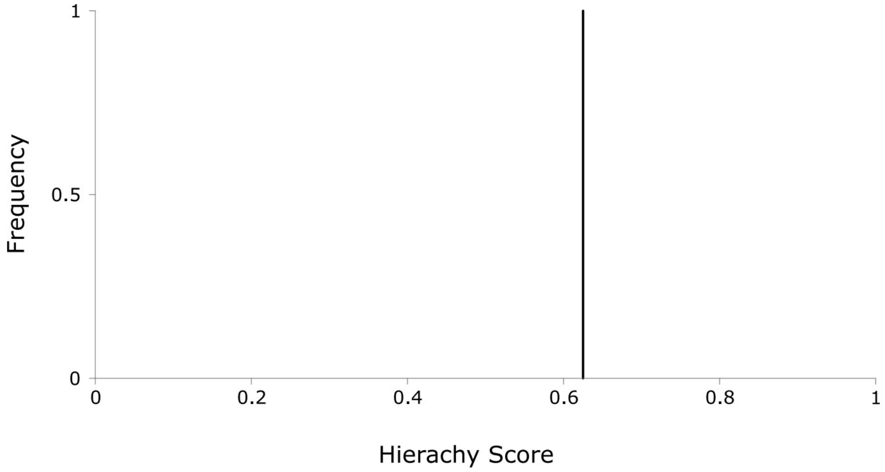


| Low H score | Median H score | High H score |
|---|---|---|
| 0.6250 | 0.6250 | 0.6250 |

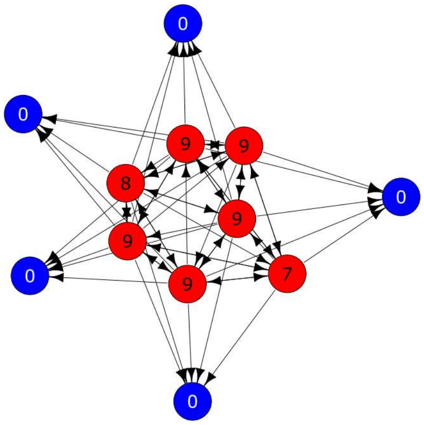


Table S45: Properties of socially efficient networks when $\min(k_{in}) = 5$, $\max(k_{out}) = 9$, universal threshold contribution is a Nash equilibrium, and pure contributors are maximized.

| | | |
|---|---|---|
| $N = 12$ | $\min(k_{in}) = 5$ | $\max(k_{out}) = 10$ |
| Parameter range: | $\frac{4}{c_t - c_p} < m < \frac{4}{c_t - c_p}$ | $\frac{c_t}{c_p} - \frac{b}{11} < p < \frac{c_t}{2c_p} - \frac{b}{11}$ |
| Max pure contributors ($C$): | $N - \left\lceil \frac{N \min(k_{in})}{\max(k_{out})} \right\rceil = 12 - \left\lceil \frac{60}{10} \right\rceil = 6$ | |
| Graph Efficiency ($E$): | $E = 1 - \frac{N \min(k_{in}) - N + 1}{(N-1)^2} = 0.5950$ | |
| Minimum Graph Hierarchy: | $\frac{2N-2C}{2N-C-1} = 12/17 = 0.7059$ | |
| Graph hierarchy score distribution from 1,000,000 random samples: | | |
| 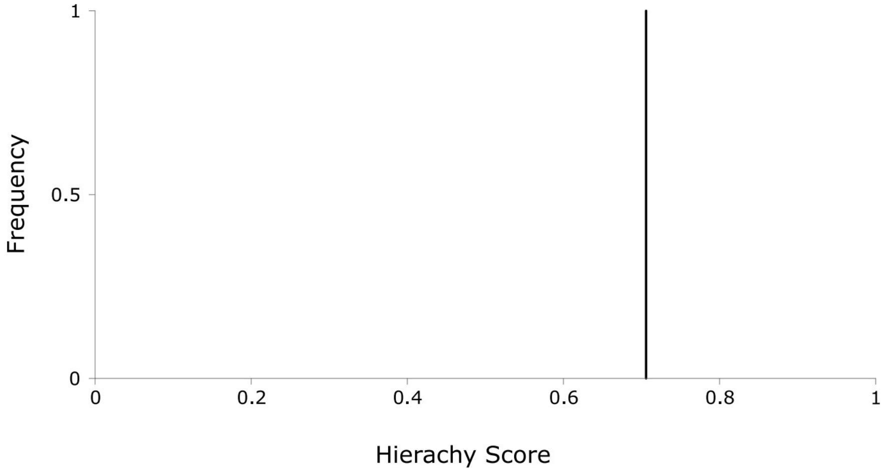 | | |
| Low H score 0.7059 | Median H score 0.7059 | High H score 0.7059 |
| | 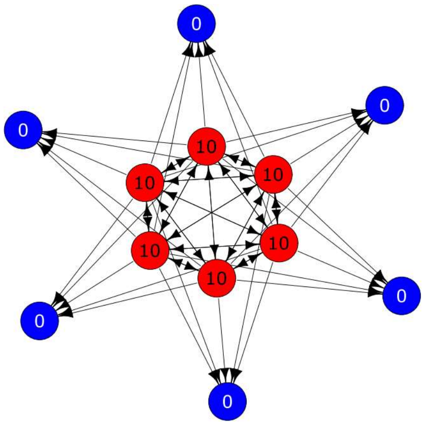 | |

Table S46: Properties of socially efficient networks when $\min(k_{in}) = 5$, $\max(k_{out}) = 10$, universal threshold contribution is a Nash equilibrium, and pure contributors are maximized.

| | | |
|---|---|---|
| $N = 12$ | $\min(k_{in}) = 5$ | $\max(k_{out}) = 11$ |
| Parameter range: | $\frac{4}{c_t - c_p} < m < \frac{4}{c_t - c_p}$ | $\frac{c_t}{c_p} - \frac{b}{11} < p < \frac{c_t}{2c_p} - \frac{b}{11}$ |
| Max pure contributors ($C$): | $N - \left\lceil \frac{N \min(k_{in})}{\max(k_{out})} \right\rceil = 12 - \left\lceil \frac{60}{11} \right\rceil = 6$ | |
| Graph Efficiency ($E$): | $E = 1 - \frac{N \min(k_{in}) - N + 1}{(N-1)^2} = 0.5950$ | |
| Minimum Graph Hierarchy: | $\frac{2N-2C}{2N-C-1} = 12/17 = 0.7059$ | |

Graph hierarchy score distribution from 1,000,000 random samples:

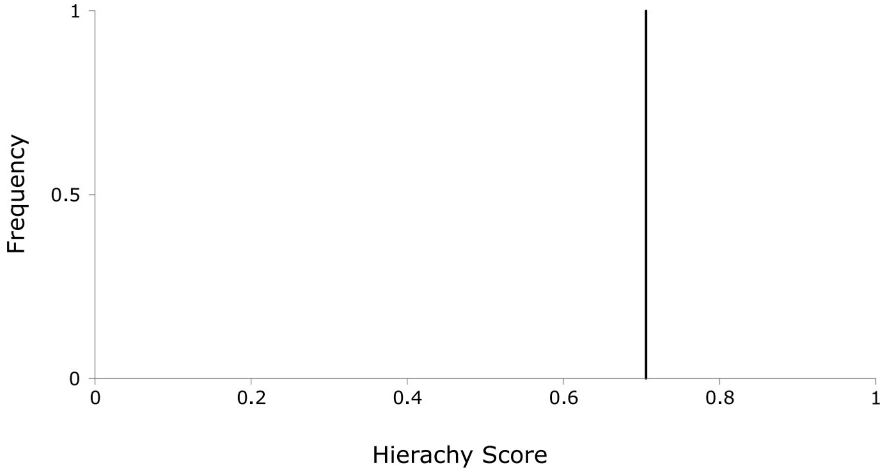


| Low H score | Median H score | High H score |
|---|---|---|
| 0.7059 | 0.7059 | 0.7059 |

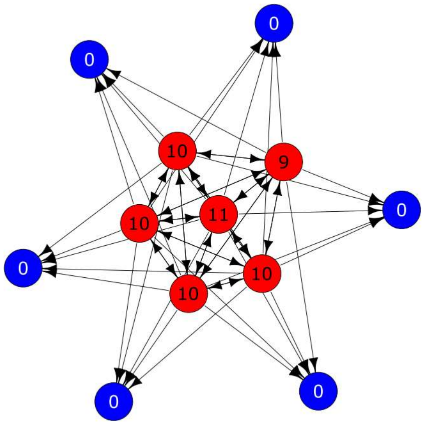


Table S47: Properties of socially efficient networks when $\min(k_{in}) = 5$, $\max(k_{out}) = 11$, universal threshold contribution is a Nash equilibrium, and pure contributors are maximized.

| | | |
|---|---|---|
| $N = 12$ | $\min(k_{in}) = 6$ | $\max(k_{out}) = 6$ |
| Parameter range: | $\frac{4}{c_t - c_p} < m < \frac{4}{c_t - c_p}$ | $\frac{c_t}{c_p} - \frac{b}{11} < p < \frac{c_t}{2c_p} - \frac{b}{11}$ |
| Max pure contributors ($C$): | $N - \left\lceil \frac{N \min(k_{in})}{\max(k_{out})} \right\rceil = 12 - \left\lceil \frac{72}{6} \right\rceil = 0$ | |
| Graph Efficiency ($E$): | $E = 1 - \frac{N \min(k_{in}) - N + 1}{(N-1)^2} = 0.4959$ | |
| Minimum Graph Hierarchy: | $\frac{2N-2C}{2N-C-1} = 0/11 = 0.0000$ | |

Graph hierarchy score distribution from 1,000,000 random samples:

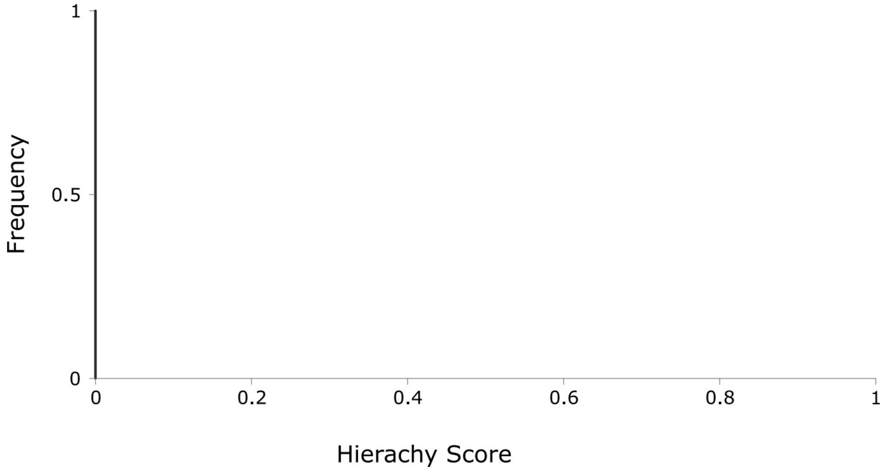


| Low H score | Median H score | High H score |
|---|---|---|
| 0.0000 | 0.0000 | 0.0000 |

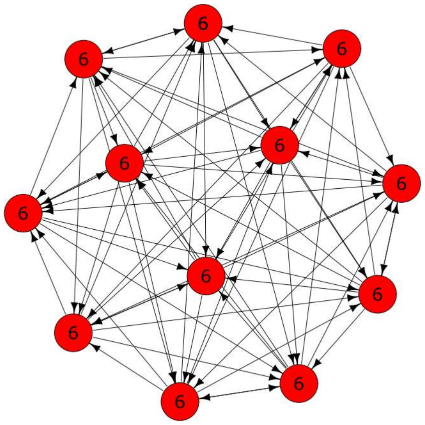


Table S48: Properties of socially efficient networks when $\min(k_{in}) = 6$, $\max(k_{out}) = 6$, universal threshold contribution is a Nash equilibrium, and pure contributors are maximized.

| | | |
|---|---|---|
| $N = 12$ | $\min(k_{in}) = 6$ | $\max(k_{out}) = 7$ |
| Parameter range: | $\frac{4}{c_t - c_p} < m < \frac{4}{c_t - c_p}$ | $\frac{c_t}{c_p} - \frac{b}{11} < p < \frac{c_t}{2c_p} - \frac{b}{11}$ |
| Max pure contributors ($C$): | $N - \left\lceil \frac{N \min(k_{in})}{\max(k_{out})} \right\rceil = 12 - \left\lceil \frac{72}{7} \right\rceil = 1$ | |
| Graph Efficiency ($E$): | $E = 1 - \frac{N \min(k_{in}) - N + 1}{(N-1)^2} = 0.4959$ | |
| Minimum Graph Hierarchy: | $\frac{2N-2C}{2N-C-1} = 2/12 = 0.1667$ | |
| Graph hierarchy score distribution from 1,000,000 random samples: 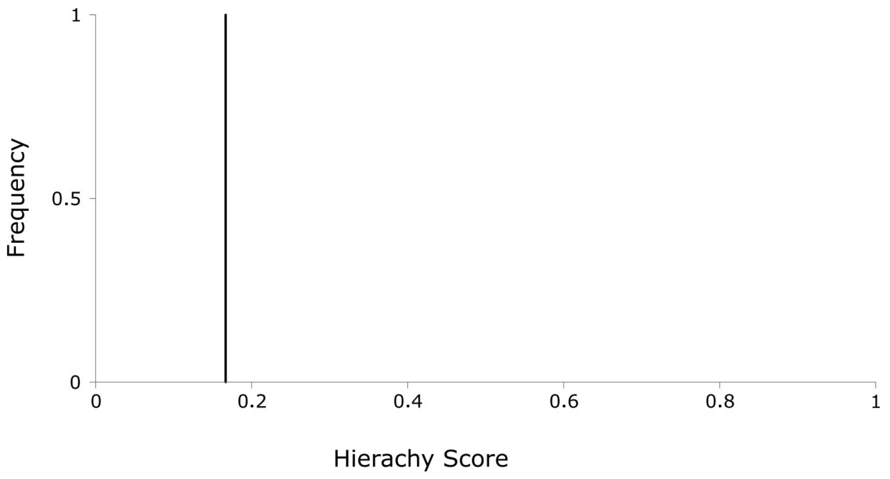  | | |
| Low H score 0.1667 | Median H score 0.1667 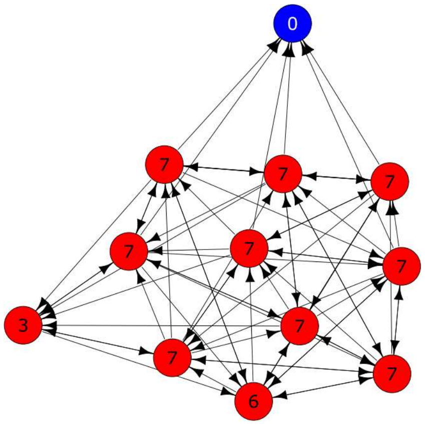  | High H score 0.1667 |

Table S49: Properties of socially efficient networks when $\min(k_{in}) = 6$, $\max(k_{out}) = 7$, universal threshold contribution is a Nash equilibrium, and pure contributors are maximized.

| $N = 12$ | $\min(k_{in}) = 6$ | $\max(k_{out}) = 8$ |
|---|---|---|

Parameter range: $\frac{4}{c_t - c_p} < m < \frac{4}{c_t - c_p}$ $\quad \frac{c_t}{c_p} - \frac{b}{11} < p < \frac{c_t}{2c_p} - \frac{b}{11}$

Max pure contributors ($C$): $N - \left\lceil \frac{N\min(k_{in})}{\max(k_{out})} \right\rceil = 12 - \left\lceil \frac{72}{8} \right\rceil = 3$

Graph Efficiency ($E$): $E = 1 - \frac{N\min(k_{in}) - N + 1}{(N-1)^2} = 0.4959$

Minimum Graph Hierarchy: $\frac{2N-2C}{2N-C-1} = 6/14 = 0.4286$

Graph hierarchy score distribution from 1,000,000 random samples:

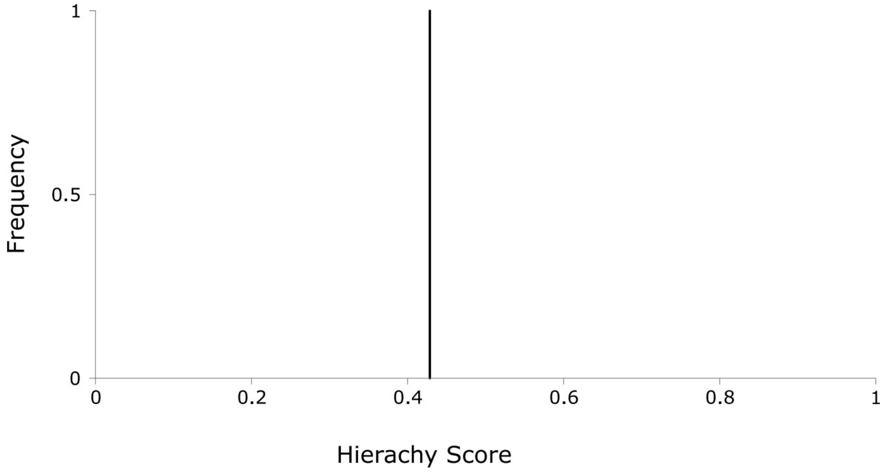


| Low H score | Median H score | High H score |
|---|---|---|
| 0.4286 | 0.4286 | 0.4286 |

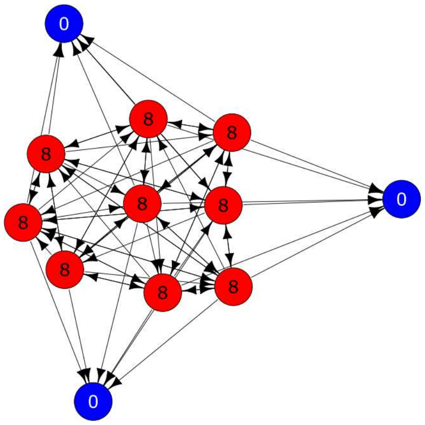

Table S50: Properties of socially efficient networks when $\min(k_{in}) = 6$, $\max(k_{out}) = 8$, universal threshold contribution is a Nash equilibrium, and pure contributors are maximized.

| | | |
|---|---|---|
| $N = 12$ | $\min(k_{in}) = 6$ | $\max(k_{out}) = 9$ |
| Parameter range: | $\frac{4}{c_t - c_p} < m < \frac{4}{c_t - c_p}$ | $\frac{c_t}{c_p} - \frac{b}{11} < p < \frac{c_t}{2c_p} - \frac{b}{11}$ |
| Max pure contributors ($C$): | $N - \left\lceil \frac{N \min(k_{in})}{\max(k_{out})} \right\rceil = 12 - \left\lceil \frac{72}{9} \right\rceil = 4$ | |
| Graph Efficiency ($E$): | $E = 1 - \frac{N \min(k_{in}) - N + 1}{(N-1)^2} = 0.4959$ | |
| Minimum Graph Hierarchy: | $\frac{2N-2C}{2N-C-1} = 8/15 = 0.5333$ | |

Graph hierarchy score distribution from 1,000,000 random samples:

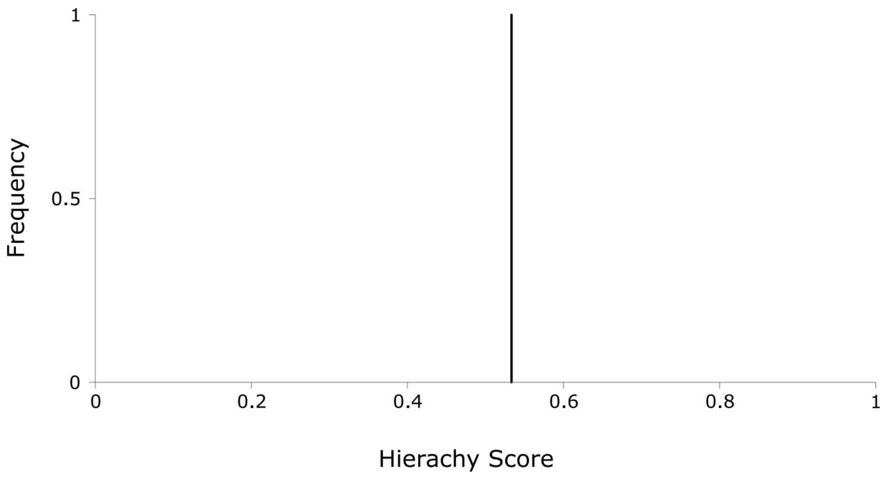


| Low H score | Median H score | High H score |
|---|---|---|
| 0.5333 | 0.5333 | 0.5333 |

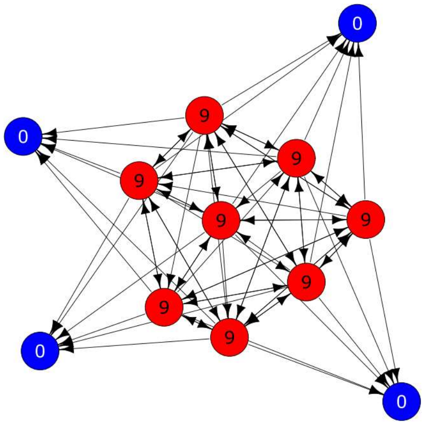


Table S51: Properties of socially efficient networks when $\min(k_{in}) = 6$, $\max(k_{out}) = 9$, universal threshold contribution is a Nash equilibrium, and pure contributors are maximized.

| | | |
|---|---|---|
| $N = 12$ | $\min(k_{in}) = 6$ | $\max(k_{out}) = 10$ |
| Parameter range: | $\frac{4}{c_t - c_p} < m < \frac{4}{c_t - c_p}$ | $\frac{c_t}{c_p} - \frac{b}{11} < p < \frac{c_t}{2c_p} - \frac{b}{11}$ |
| Max pure contributors ($C$): | $N - \left\lceil \frac{N \min(k_{in})}{\max(k_{out})} \right\rceil = 12 - \left\lceil \frac{72}{10} \right\rceil = 4$ | |
| Graph Efficiency ($E$): | $E = 1 - \frac{N \min(k_{in}) - N + 1}{(N-1)^2} = 0.4959$ | |
| Minimum Graph Hierarchy: | $\frac{2N-2C}{2N-C-1} = 8/15 = 0.5333$ | |

Graph hierarchy score distribution from 1,000,000 random samples:

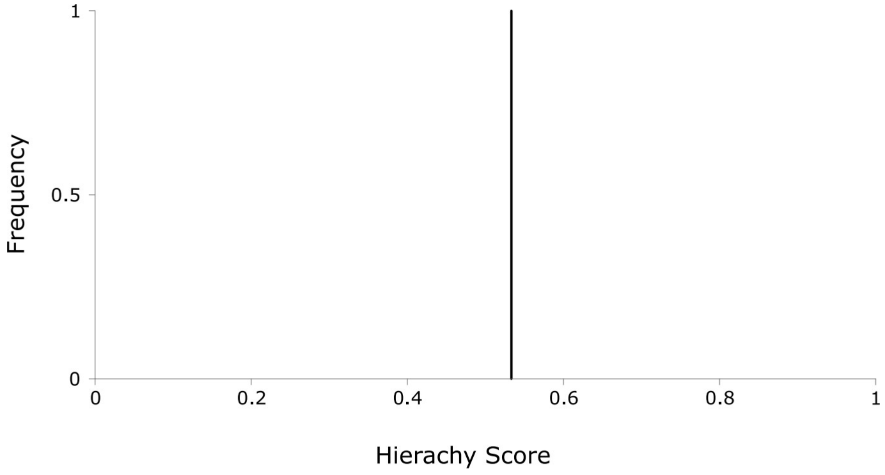


| Low H score | Median H score | High H score |
|---|---|---|
| 0.5333 | 0.5333 | 0.6500 |

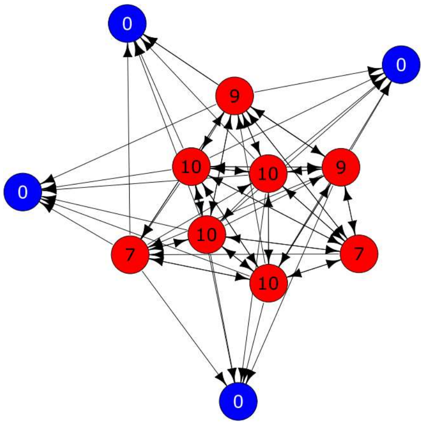


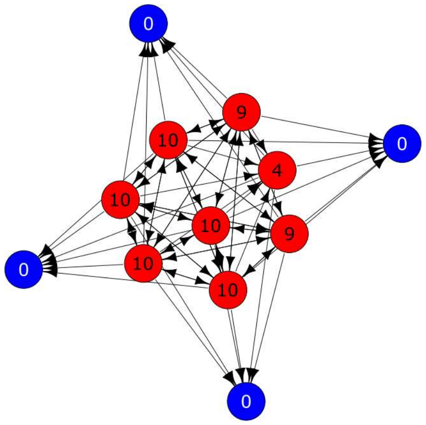


Table S52: Properties of socially efficient networks when $\min(k_{in}) = 6$, $\max(k_{out}) = 10$, universal threshold contribution is a Nash equilibrium, and pure contributors are maximized.

| | | |
|---|---|---|
| $N = 12$ | $\min(k_{in}) = 6$ | $\max(k_{out}) = 11$ |
| Parameter range: | $\frac{4}{c_t - c_p} < m < \frac{4}{c_t - c_p}$ | $\frac{c_t}{c_p} - \frac{b}{11} < p < \frac{c_t}{2c_p} - \frac{b}{11}$ |
| Max pure contributors ($C$): | $N - \left\lceil \frac{N \min(k_{in})}{\max(k_{out})} \right\rceil = 12 - \left\lceil \frac{72}{11} \right\rceil = 5$ | |
| Graph Efficiency ($E$): | $E = 1 - \frac{N \min(k_{in}) - N + 1}{(N-1)^2} = 0.4959$ | |
| Minimum Graph Hierarchy: | $\frac{2N-2C}{2N-C-1} = 10/16 = 0.6250$ | |

Graph hierarchy score distribution from 1,000,000 random samples:

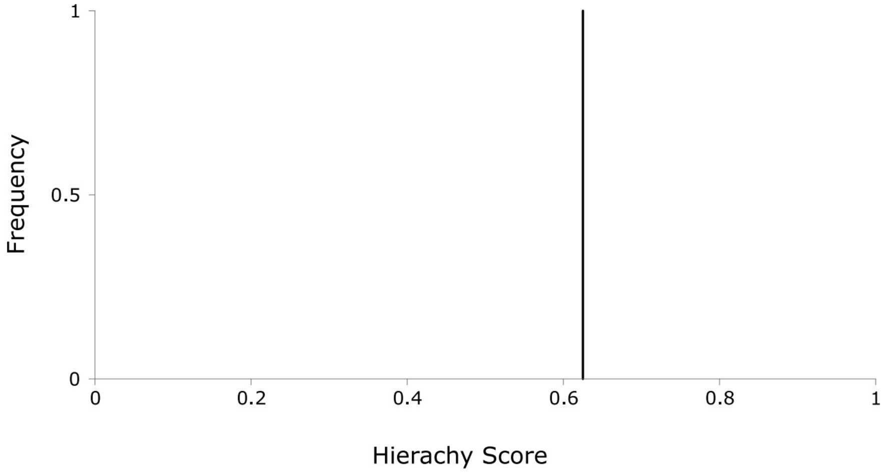


| Low H score | Median H score | High H score |
|---|---|---|
| 0.6250 | 0.6250 | 0.6250 |

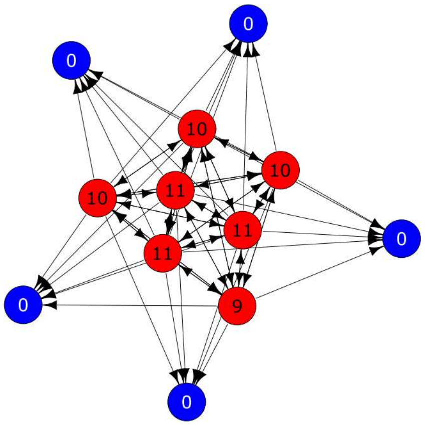


Table S53: Properties of socially efficient networks when $\min(k_{in}) = 6$, $\max(k_{out}) = 11$, universal threshold contribution is a Nash equilibrium, and pure contributors are maximized.

| | | |
|---|---|---|
| $N = 12$ | $\min(k_{in}) = 7$ | $\max(k_{out}) = 7$ |
| Parameter range: | $\frac{4}{c_t - c_p} < m < \frac{4}{c_t - c_p}$ | $\frac{c_t}{c_p} - \frac{b}{11} < p < \frac{c_t}{2c_p} - \frac{b}{11}$ |
| Max pure contributors ($C$): | $N - \left\lceil \frac{N \min(k_{in})}{\max(k_{out})} \right\rceil = 12 - \left\lceil \frac{84}{7} \right\rceil = 0$ | |
| Graph Efficiency ($E$): | $E = 1 - \frac{N \min(k_{in}) - N + 1}{(N-1)^2} = 0.3967$ | |
| Minimum Graph Hierarchy: | $\frac{2N-2C}{2N-C-1} = 0/11 = 0.0000$ | |

Graph hierarchy score distribution from 1,000,000 random samples:

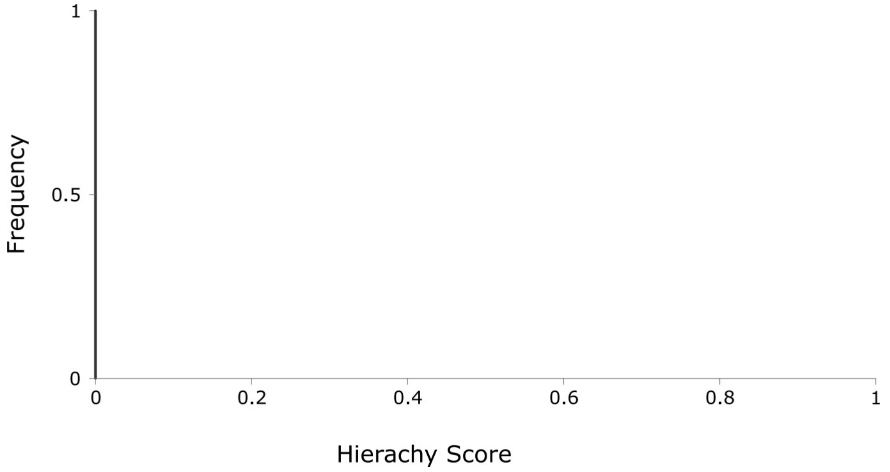


| Low H score | Median H score | High H score |
|---|---|---|
| 0.0000 | 0.0000 | 0.0000 |

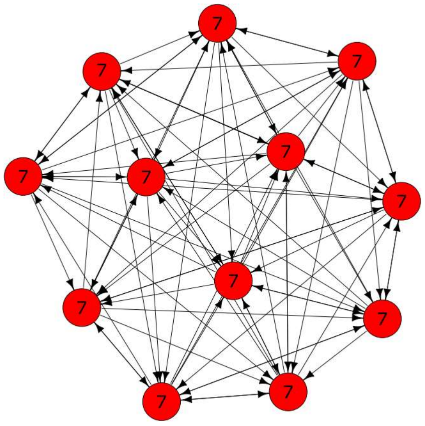

Table S54: Properties of socially efficient networks when $\min(k_{in}) = 7$, $\max(k_{out}) = 7$, universal threshold contribution is a Nash equilibrium, and pure contributors are maximized.

| | | |
|---|---|---|
| $N = 12$ | $\min(k_{in}) = 7$ | $\max(k_{out}) = 8$ |
| Parameter range: | $\frac{4}{c_t - c_p} < m < \frac{4}{c_t - c_p}$ | $\frac{c_t}{c_p} - \frac{b}{11} < p < \frac{c_t}{2c_p} - \frac{b}{11}$ |
| Max pure contributors ($C$): | $N - \left\lceil \frac{N \min(k_{in})}{\max(k_{out})} \right\rceil = 12 - \left\lceil \frac{84}{8} \right\rceil = 1$ | |
| Graph Efficiency ($E$): | $E = 1 - \frac{N \min(k_{in}) - N + 1}{(N-1)^2} = 0.3967$ | |
| Minimum Graph Hierarchy: | $\frac{2N-2C}{2N-C-1} = 2/12 = 0.1667$ | |

Graph hierarchy score distribution from 1,000,000 random samples:

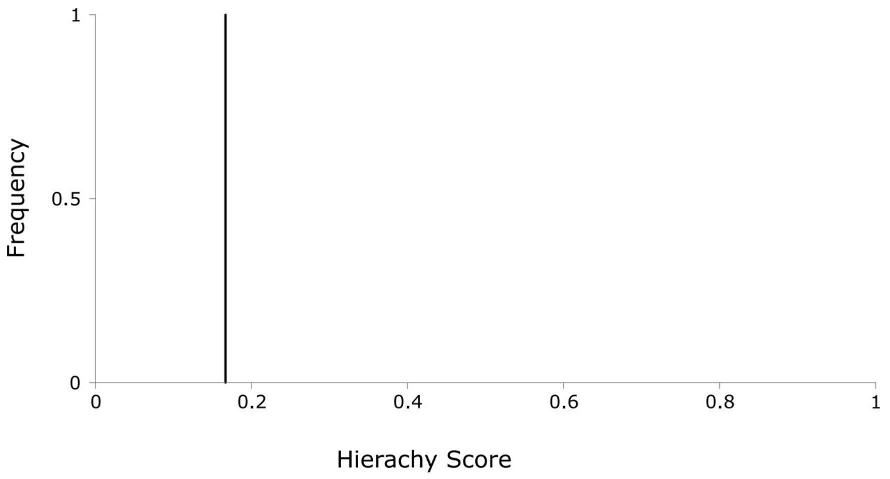


| Low H score | Median H score | High H score |
|---|---|---|
| 0.1667 | 0.1667 | 0.1667 |

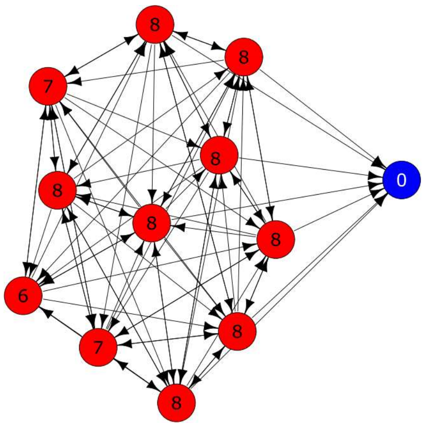


Table S55: Properties of socially efficient networks when $\min(k_{in}) = 7$, $\max(k_{out}) = 8$, universal threshold contribution is a Nash equilibrium, and pure contributors are maximized.

| | | |
|---|---|---|
| $N = 12$ | $\min(k_{in}) = 7$ | $\max(k_{out}) = 9$ |
| Parameter range: | $\frac{4}{c_t - c_p} < m < \frac{4}{c_t - c_p}$ | $\frac{c_t}{c_p} - \frac{b}{11} < p < \frac{c_t}{2c_p} - \frac{b}{11}$ |
| Max pure contributors ($C$): | $N - \left\lceil \frac{N \min(k_{in})}{\max(k_{out})} \right\rceil = 12 - \left\lceil \frac{84}{9} \right\rceil = 2$ | |
| Graph Efficiency ($E$): | $E = 1 - \frac{N \min(k_{in}) - N + 1}{(N-1)^2} = 0.3967$ | |
| Minimum Graph Hierarchy: | $\frac{2N-2C}{2N-C-1} = 4/13 = 0.3077$ | |

Graph hierarchy score distribution from 1,000,000 random samples:

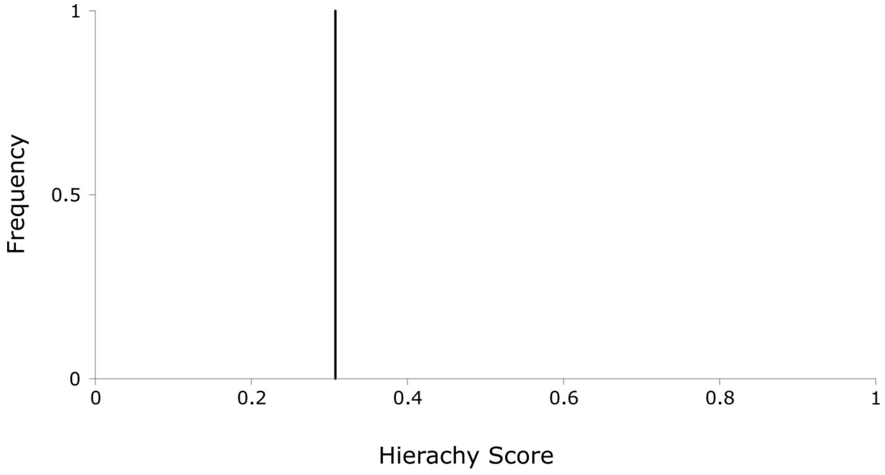


| Low H score | Median H score | High H score |
|---|---|---|
| 0.3077 | 0.3077 | 0.3077 |

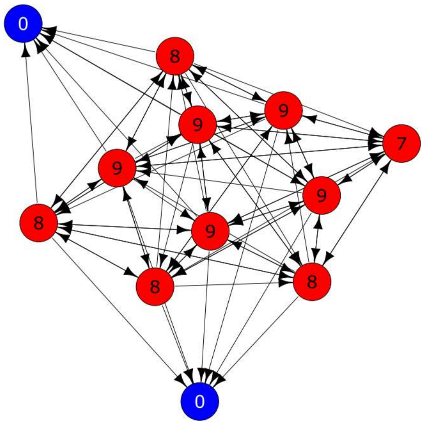


Table S56: Properties of socially efficient networks when $\min(k_{in}) = 7$, $\max(k_{out}) = 9$, universal threshold contribution is a Nash equilibrium, and pure contributors are maximized.

| | | |
|---|---|---|
| $N = 12$ | $\min(k_{in}) = 7$ | $\max(k_{out}) = 10$ |
| Parameter range: | $\frac{4}{c_t - c_p} < m < \frac{4}{c_t - c_p}$ | $\frac{c_t}{c_p} - \frac{b}{11} < p < \frac{c_t}{2c_p} - \frac{b}{11}$ |
| Max pure contributors ($C$): | $N - \left\lceil \frac{N\min(k_{in})}{\max(k_{out})} \right\rceil = 12 - \left\lceil \frac{84}{10} \right\rceil = 3$ | |
| Graph Efficiency ($E$): | $E = 1 - \frac{N\min(k_{in}) - N + 1}{(N-1)^2} = 0.3967$ | |
| Minimum Graph Hierarchy: | $\frac{2N-2C}{2N-C-1} = 6/14 = 0.4286$ | |

Graph hierarchy score distribution from 1,000,000 random samples:

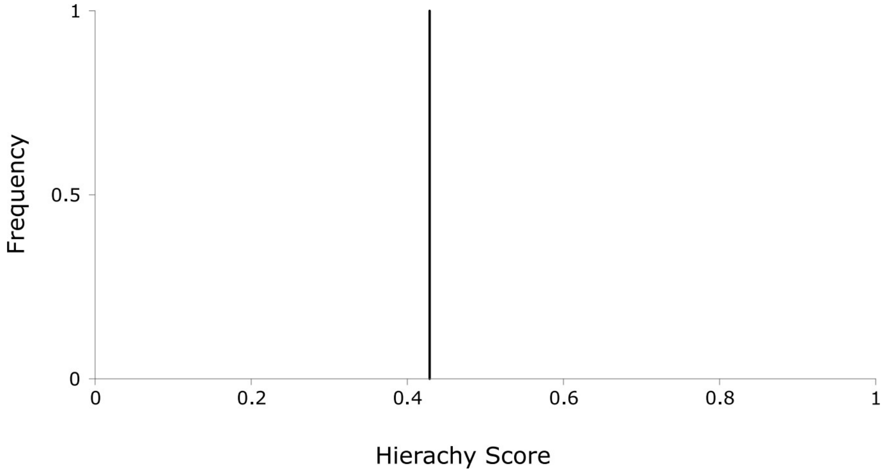


| Low H score | Median H score | High H score |
|---|---|---|
| 0.4286 | 0.4286 | 0.4286 |

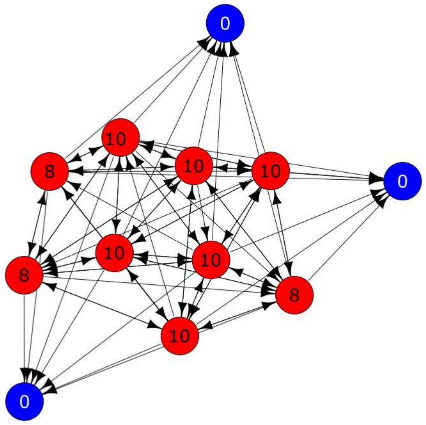


Table S57: Properties of socially efficient networks when $\min(k_{in}) = 7$, $\max(k_{out}) = 10$, universal threshold contribution is a Nash equilibrium, and pure contributors are maximized.

| $N = 12$ | $\min(k_{in}) = 7$ | $\max(k_{out}) = 11$ |
|---|---|---|
| Parameter range: | $\frac{4}{c_t - c_p} < m < \frac{4}{c_t - c_p}$ | $\frac{c_t}{c_p} - \frac{b}{11} < p < \frac{c_t}{2c_p} - \frac{b}{11}$ |
| Max pure contributors ($C$): | $N - \left\lceil \frac{N \min(k_{in})}{\max(k_{out})} \right\rceil = 12 - \left\lceil \frac{84}{11} \right\rceil = 4$ | |
| Graph Efficiency ($E$): | $E = 1 - \frac{N \min(k_{in}) - N + 1}{(N-1)^2} = 0.3967$ | |
| Minimum Graph Hierarchy: | $\frac{2N - 2C}{2N - C - 1} = 8/15 = 0.5333$ | |

Graph hierarchy score distribution from 1,000,000 random samples:

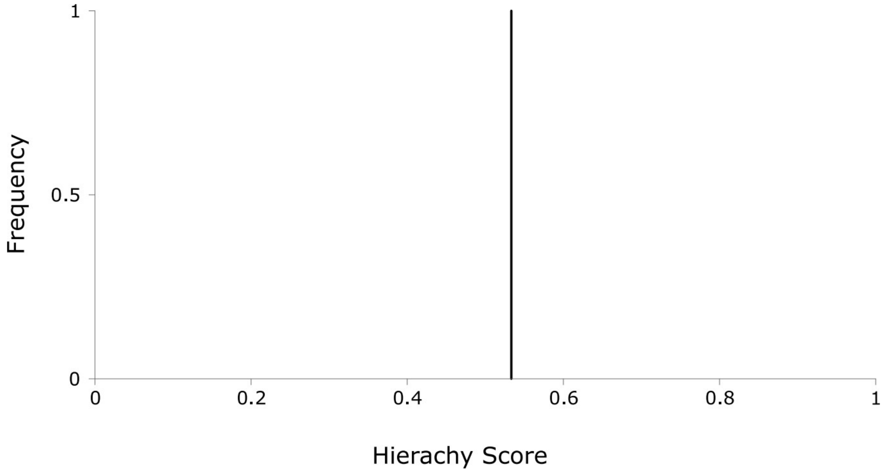


| Low H score | Median H score | High H score |
|---|---|---|
| 0.5333 | 0.5333 | 0.5333 |

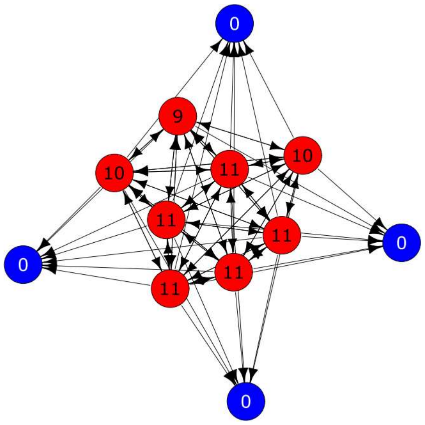


Table S58: Properties of socially efficient networks when $\min(k_{in}) = 7$, $\max(k_{out}) = 11$, universal threshold contribution is a Nash equilibrium, and pure contributors are maximized.

| | | |
|---|---|---|
| $N = 12$ | $\min(k_{in}) = 8$ | $\max(k_{out}) = 8$ |
| Parameter range: | $\frac{4}{c_t - c_p} < m < \frac{4}{c_t - c_p}$ | $\frac{c_t}{c_p} - \frac{b}{11} < p < \frac{c_t}{2c_p} - \frac{b}{11}$ |
| Max pure contributors ($C$): | $N - \left\lceil \frac{N\min(k_{in})}{\max(k_{out})} \right\rceil = 12 - \left\lceil \frac{96}{8} \right\rceil = 0$ | |
| Graph Efficiency ($E$): | $E = 1 - \frac{N\min(k_{in}) - N + 1}{(N-1)^2} = 0.2975$ | |
| Minimum Graph Hierarchy: | $\frac{2N-2C}{2N-C-1} = 0/11 = 0.0000$ | |

Graph hierarchy score distribution from 1,000,000 random samples:

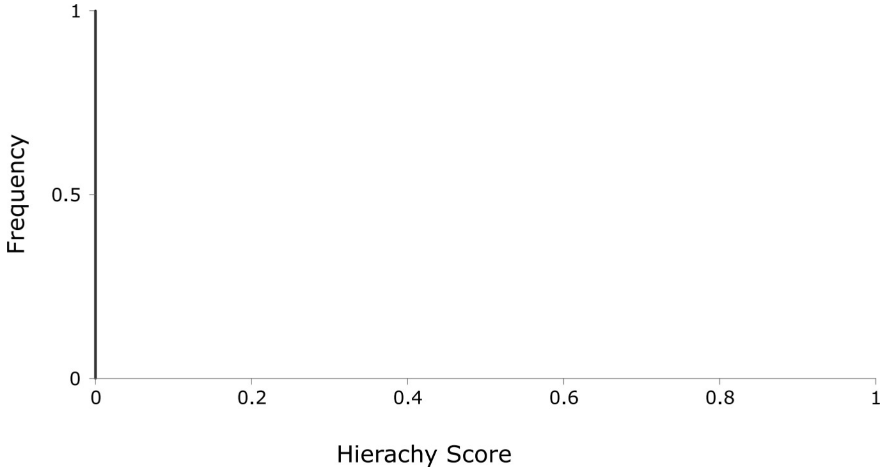


| Low H score | Median H score | High H score |
|---|---|---|
| 0.0000 | 0.0000 | 0.0000 |

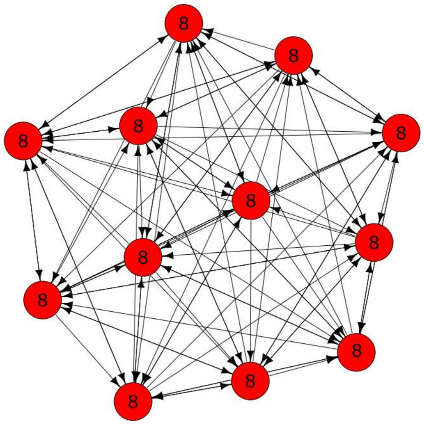


Table S59: Properties of socially efficient networks when $\min(k_{in}) = 8$, $\max(k_{out}) = 8$, universal threshold contribution is a Nash equilibrium, and pure contributors are maximized.

| $N = 12$ | $\min(k_{in}) = 8$ | $\max(k_{out}) = 9$ |
|---|---|---|
| Parameter range: | $\frac{4}{c_t - c_p} < m < \frac{4}{c_t - c_p}$ | $\frac{c_t}{c_p} - \frac{b}{11} < p < \frac{c_t}{2c_p} - \frac{b}{11}$ |
| Max pure contributors ($C$): | $N - \left\lceil \frac{N\min(k_{in})}{\max(k_{out})} \right\rceil = 12 - \left\lceil \frac{96}{9} \right\rceil = 1$ | |
| Graph Efficiency ($E$): | $E = 1 - \frac{N\min(k_{in}) - N + 1}{(N-1)^2} = 0.2975$ | |
| Minimum Graph Hierarchy: | $\frac{2N-2C}{2N-C-1} = 2/12 = 0.1667$ | |

Graph hierarchy score distribution from 1,000,000 random samples:

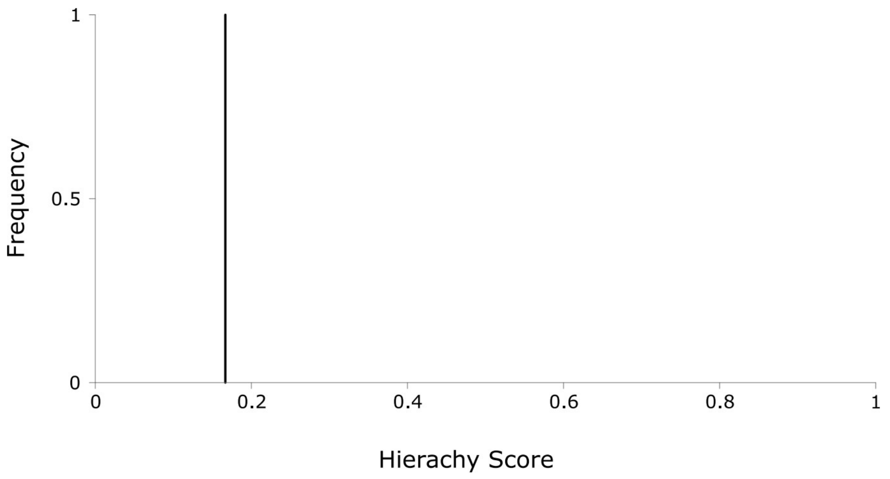


| Low H score | Median H score | High H score |
|---|---|---|
| 0.1667 | 0.1667 | 0.1667 |

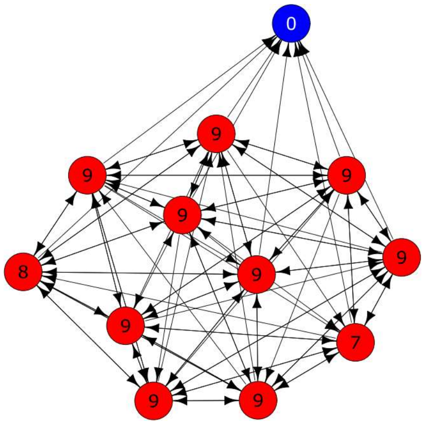

Table S60: Properties of socially efficient networks when $\min(k_{in}) = 8$, $\max(k_{out}) = 9$, universal threshold contribution is a Nash equilibrium, and pure contributors are maximized.

| $N = 12$ | $\min(k_{in}) = 8$ | $\max(k_{out}) = 10$ |
|---|---|---|
| Parameter range: | $\frac{4}{c_t - c_p} < m < \frac{4}{c_t - c_p}$ | $\frac{c_t}{c_p} - \frac{b}{11} < p < \frac{c_t}{2c_p} - \frac{b}{11}$ |
| Max pure contributors ($C$): | $N - \left\lceil \frac{N \min(k_{in})}{\max(k_{out})} \right\rceil = 12 - \left\lceil \frac{96}{10} \right\rceil = 2$ | |
| Graph Efficiency ($E$): | $E = 1 - \frac{N \min(k_{in}) - N + 1}{(N-1)^2} = 0.2975$ | |
| Minimum Graph Hierarchy: | $\frac{2N-2C}{2N-C-1} = 4/13 = 0.3077$ | |
| Graph hierarchy score distribution from 1,000,000 random samples: | | |

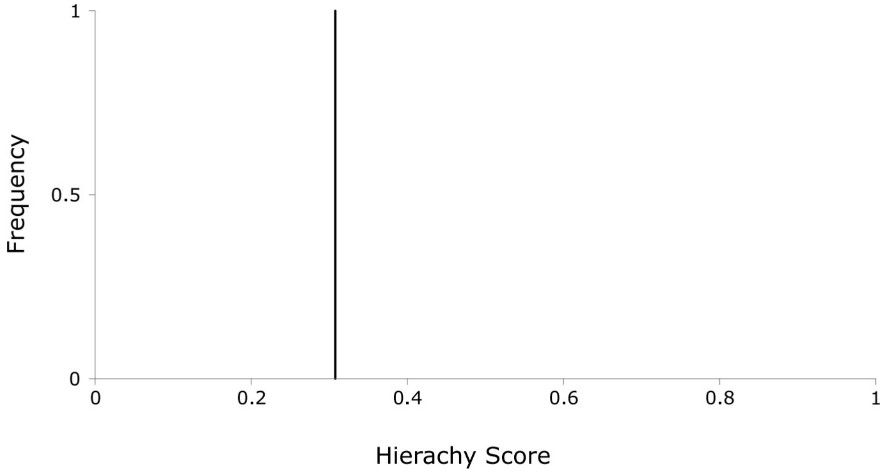


| Low H score | Median H score | High H score |
|---|---|---|
| 0.3077 | 0.3077 | 0.3077 |

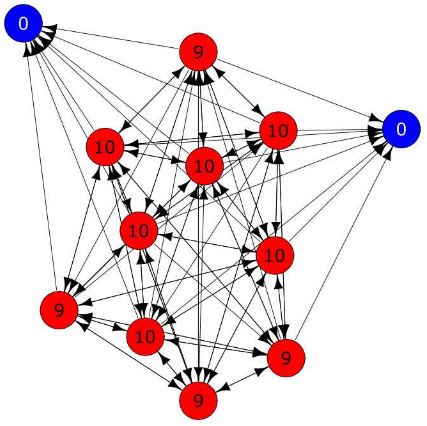

Table S61: Properties of socially efficient networks when $\min(k_{in}) = 8$, $\max(k_{out}) = 10$, universal threshold contribution is a Nash equilibrium, and pure contributors are maximized.

| | | |
|---|---|---|
| $N = 12$ | $\min(k_{in}) = 8$ | $\max(k_{out}) = 11$ |
| Parameter range: | $\frac{4}{c_t - c_p} < m < \frac{4}{c_t - c_p}$ | $\frac{c_t}{c_p} - \frac{b}{11} < p < \frac{c_t}{2c_p} - \frac{b}{11}$ |
| Max pure contributors ($C$): | $N - \left\lceil \frac{N \min(k_{in})}{\max(k_{out})} \right\rceil = 12 - \left\lceil \frac{96}{11} \right\rceil = 3$ | |
| Graph Efficiency ($E$): | $E = 1 - \frac{N \min(k_{in}) - N + 1}{(N-1)^2} = 0.2975$ | |
| Minimum Graph Hierarchy: | $\frac{2N - 2C}{2N - C - 1} = 6/14 = 0.4286$ | |
| Graph hierarchy score distribution from 1,000,000 random samples: | | |

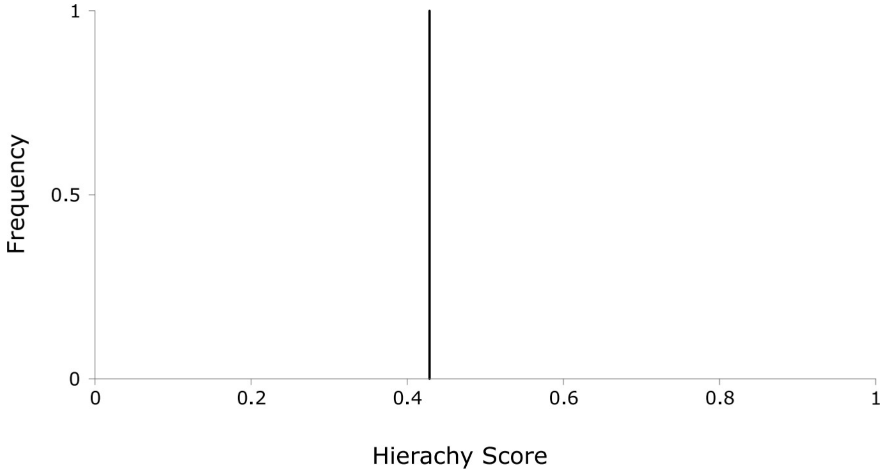


| Low H score | Median H score | High H score |
|---|---|---|
| 0.4286 | 0.4286 | 0.4286 |

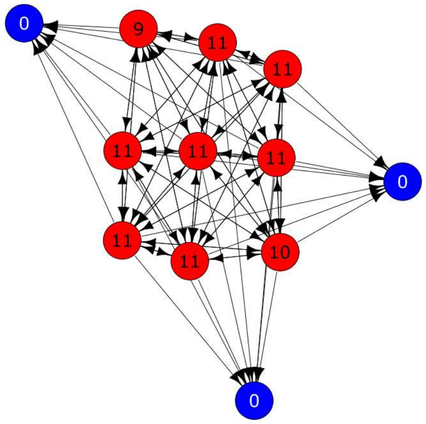

Table S62: Properties of socially efficient networks when $\min(k_{in}) = 8$, $\max(k_{out}) = 11$, universal threshold contribution is a Nash equilibrium, and pure contributors are maximized.

| | | |
|---|---|---|
| $N = 12$ | $\min(k_{in}) = 9$ | $\max(k_{out}) = 9$ |
| Parameter range: | $\frac{4}{c_t - c_p} < m < \frac{4}{c_t - c_p}$ | $\frac{c_t}{c_p} - \frac{b}{11} < p < \frac{c_t}{2c_p} - \frac{b}{11}$ |
| Max pure contributors ($C$): | $N - \left\lceil \frac{N \min(k_{in})}{\max(k_{out})} \right\rceil = 12 - \left\lceil \frac{108}{9} \right\rceil = 0$ | |
| Graph Efficiency ($E$): | $E = 1 - \frac{N \min(k_{in}) - N + 1}{(N-1)^2} = 0.1983$ | |
| Minimum Graph Hierarchy: | $\frac{2N-2C}{2N-C-1} = 0/11 = 0.0000$ | |

Graph hierarchy score distribution from 1,000,000 random samples:

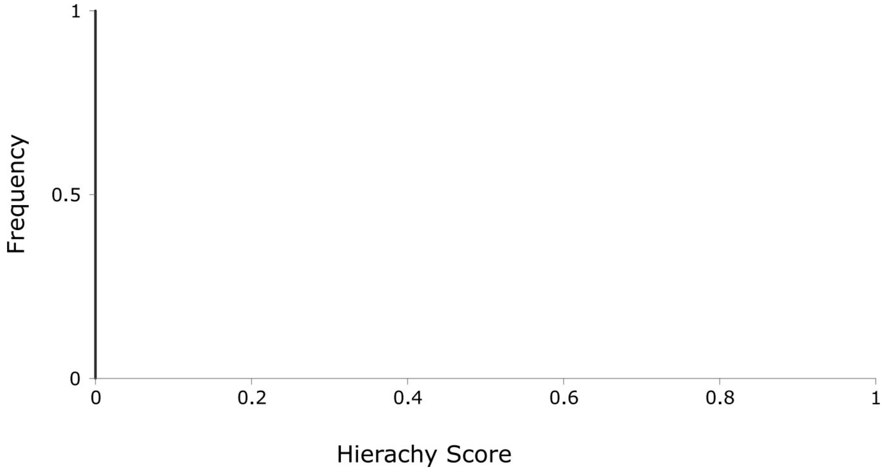


| Low H score | Median H score | High H score |
|---|---|---|
| 0.0000 | 0.0000 | 0.0000 |

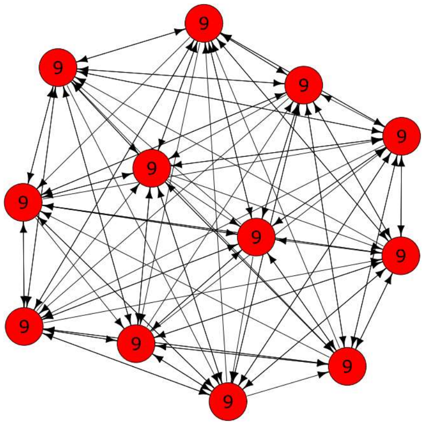


Table S63: Properties of socially efficient networks when $\min(k_{in}) = 9$, $\max(k_{out}) = 9$, universal threshold contribution is a Nash equilibrium, and pure contributors are maximized.

| | | |
|---|---|---|
| $N = 12$ | $\min(k_{in}) = 9$ | $\max(k_{out}) = 10$ |
| Parameter range: | $\frac{4}{ct-c_p} < m < \frac{4}{ct-c_p}$ | $\frac{c_t}{c_p} - \frac{b}{11} < p < \frac{c_t}{2c_p} - \frac{b}{11}$ |
| Max pure contributors ($C$): | $N - \left\lceil \frac{N\min(k_{in})}{\max(k_{out})} \right\rceil = 12 - \left\lceil \frac{108}{10} \right\rceil = 1$ | |
| Graph Efficiency ($E$): | $E = 1 - \frac{N\min(k_{in})-N+1}{(N-1)^2} = 0.1983$ | |
| Minimum Graph Hierarchy: | $\frac{2N-2C}{2N-C-1} = 2/12 = 0.1667$ | |

Graph hierarchy score distribution from 1,000,000 random samples:

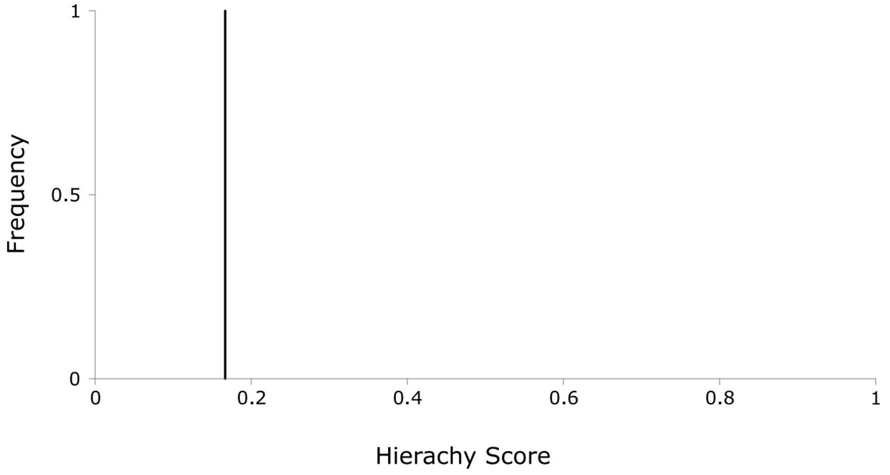


| Low H score | Median H score | High H score |
|---|---|---|
| 0.1667 | 0.1667 | 0.1667 |

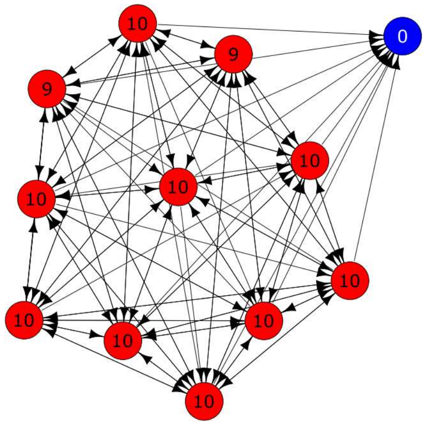

Table S64: Properties of socially efficient networks when $\min(k_{in}) = 9$, $\max(k_{out}) = 10$, universal threshold contribution is a Nash equilibrium, and pure contributors are maximized.

| | | |
|---|---|---|
| $N = 12$ | $\min(k_{in}) = 9$ | $\max(k_{out}) = 11$ |
| Parameter range: | $\frac{4}{c_t - c_p} < m < \frac{4}{c_t - c_p}$ | $\frac{c_t}{c_p} - \frac{b}{11} < p < \frac{c_t}{2c_p} - \frac{b}{11}$ |
| Max pure contributors ($C$): | $N - \left\lceil \frac{N \min(k_{in})}{\max(k_{out})} \right\rceil = 12 - \left\lceil \frac{108}{11} \right\rceil = 2$ | |
| Graph Efficiency ($E$): | $E = 1 - \frac{N \min(k_{in}) - N + 1}{(N-1)^2} = 0.1983$ | |
| Minimum Graph Hierarchy: | $\frac{2N-2C}{2N-C-1} = 4/13 = 0.3077$ | |

Graph hierarchy score distribution from 1,000,000 random samples:

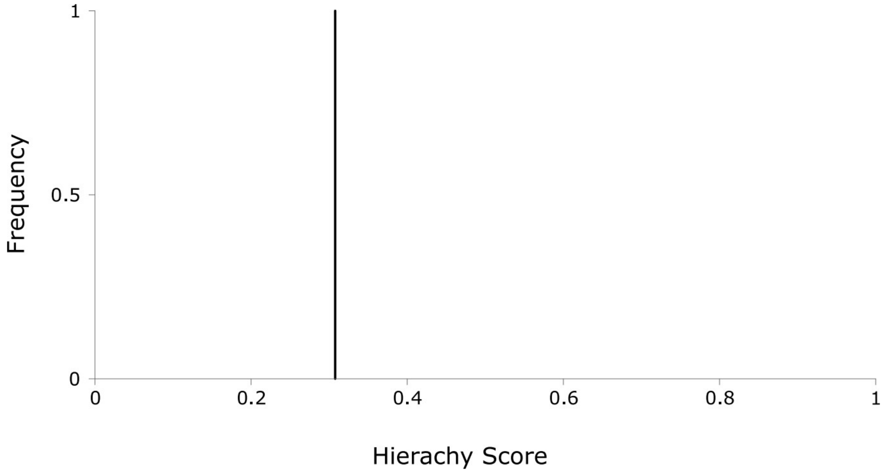


| Low H score | Median H score | High H score |
|---|---|---|
| 0.3077 | 0.3077 | 0.3077 |

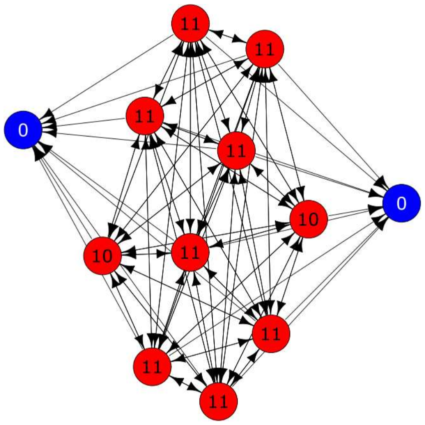

Table S65: Properties of socially efficient networks when $\min(k_{in}) = 9$, $\max(k_{out}) = 11$, universal threshold contribution is a Nash equilibrium, and pure contributors are maximized.

| $N = 12$ | $\min(k_{in}) = 10$ | $\max(k_{out}) = 10$ |
|---|---|---|
| Parameter range: | $\frac{4}{c_t - c_p} < m < \frac{4}{c_t - c_p}$ | $\frac{c_t}{c_p} - \frac{b}{11} < p < \frac{c_t}{2c_p} - \frac{b}{11}$ |
| Max pure contributors ($C$): | $N - \left\lceil \frac{N \min(k_{in})}{\max(k_{out})} \right\rceil = 12 - \left\lceil \frac{120}{10} \right\rceil = 0$ | |
| Graph Efficiency ($E$): | $E = 1 - \frac{N \min(k_{in}) - N + 1}{(N-1)^2} = 0.0992$ | |
| Minimum Graph Hierarchy: | $\frac{2N-2C}{2N-C-1} = 0/11 = 0.0000$ | |

Graph hierarchy score distribution from 1,000,000 random samples:

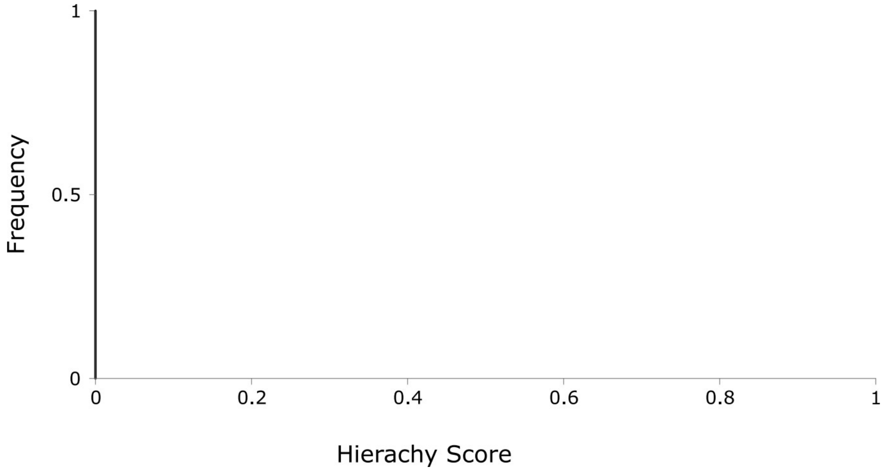


| Low H score | Median H score | High H score |
|---|---|---|
| 0.0000 | 0.0000 | 0.0000 |

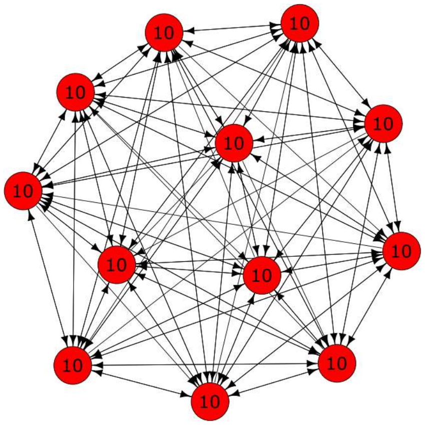


Table S66: Properties of socially efficient networks when $\min(k_{in}) = 10$, $\max(k_{out}) = 10$, universal threshold contribution is a Nash equilibrium, and pure contributors are maximized.

| | | |
|---|---|---|
| $N = 12$ | $\min(k_{in}) = 10$ | $\max(k_{out}) = 11$ |
| Parameter range: | $\frac{4}{c_t - c_p} < m < \frac{4}{c_t - c_p}$ | $\frac{c_t}{c_p} - \frac{b}{11} < p < \frac{c_t}{2c_p} - \frac{b}{11}$ |
| Max pure contributors ($C$): | $N - \left\lceil \frac{N \min(k_{in})}{\max(k_{out})} \right\rceil = 12 - \left\lceil \frac{120}{11} \right\rceil = 1$ | |
| Graph Efficiency ($E$): | $E = 1 - \frac{N \min(k_{in}) - N + 1}{(N-1)^2} = 0.0992$ | |
| Minimum Graph Hierarchy: | $\frac{2N-2C}{2N-C-1} = 2/12 = 0.1667$ | |

Graph hierarchy score distribution from 1,000,000 random samples:

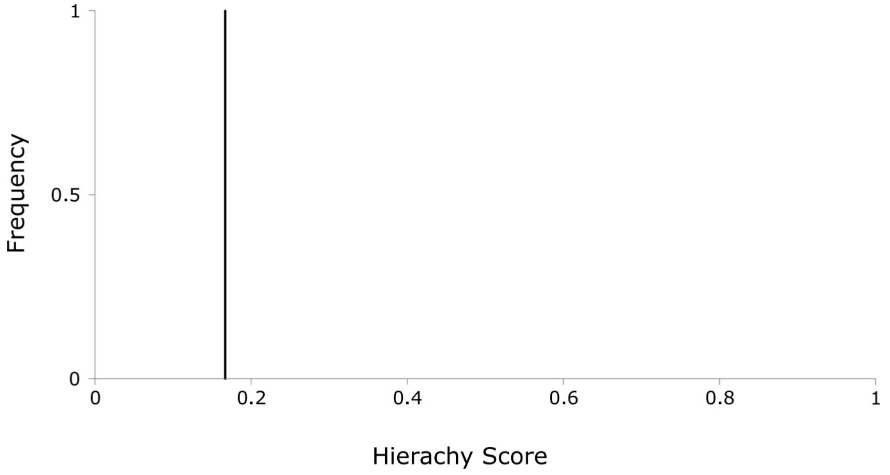


| Low H score | Median H score | High H score |
|---|---|---|
| 0.1667 | 0.1667 | 0.1667 |

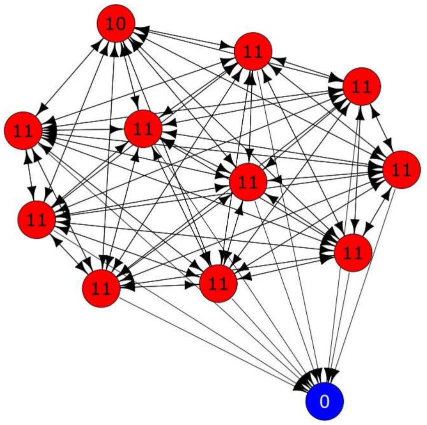


Table S67: Properties of socially efficient networks when $\min(k_{in}) = 10$, $\max(k_{out}) = 11$, universal threshold contribution is a Nash equilibrium, and pure contributors are maximized.

| | | |
|---|---|---|
| $N = 12$ | $\min(k_{in}) = 11$ | $\max(k_{out}) = 11$ |
| Parameter range: | $\frac{4}{c_t - c_p} < m < \frac{4}{c_t - c_p}$ | $\frac{c_t}{c_p} - \frac{b}{11} < p < \frac{c_t}{2c_p} - \frac{b}{11}$ |
| Max pure contributors ($C$): | $N - \left\lceil \frac{N \min(k_{in})}{\max(k_{out})} \right\rceil = 12 - \left\lceil \frac{132}{11} \right\rceil = 0$ | |
| Graph Efficiency ($E$): | $E = 1 - \frac{N \min(k_{in}) - N + 1}{(N-1)^2} = 0.0000$ | |
| Minimum Graph Hierarchy: | $\frac{2N - 2C}{2N - C - 1} = 0/11 = 0.0000$ | |

Graph hierarchy score distribution from 1,000,000 random samples:

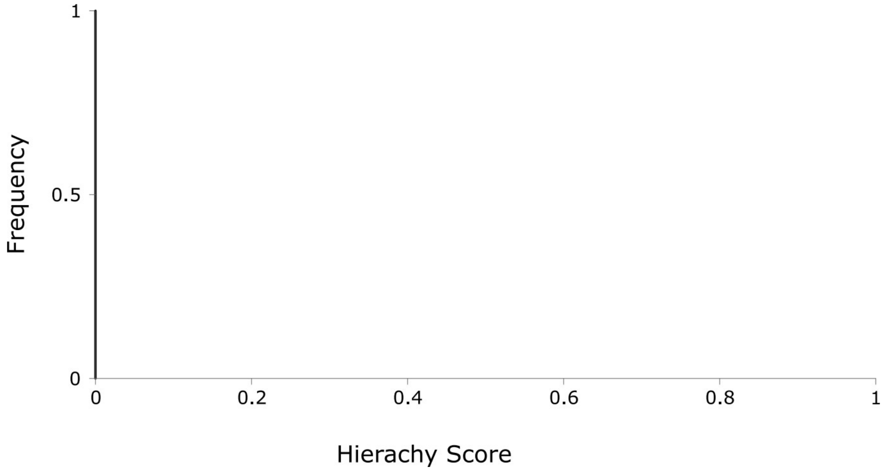


| Low H score | Median H score | High H score |
|---|---|---|
| 0.0000 | 0.0000 | 0.0000 |

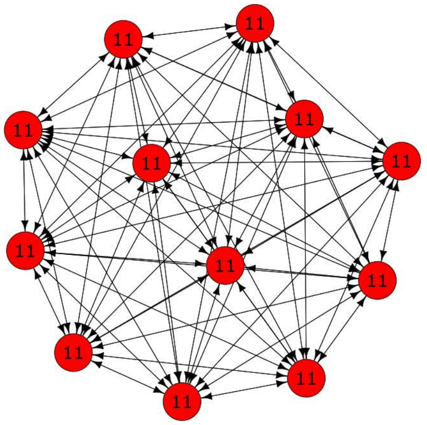


Table S68: Properties of socially efficient networks when $\min(k_{in}) = 11$, $\max(k_{out}) = 11$, universal threshold contribution is a Nash equilibrium, and pure contributors are maximized.

# S13 All optimal networks and hierarchy scores when minimum in-degree is one.

Appendix S9 describes how to calculate a lower bound for graph hierarchy scores for socially efficient networks where universal threshold contribution is a Nash equilibrium and the network has the maximum number of pure contributors. Appendix S12, lists the results of simulations showing that for most parameter combinations, the lower bound network hierarchy score was also the only one found. For some parameter combinations, the simulations showed that networks with greater hierarchy scores are possible and, in some cases, easier to find with randomly generated networks. These combinations where most prominent in cases where $\min(k_{in}) = 1$ and $\max(k_{out}) > 1$. These conditions are also especially interesting because they generate the most tree-like hierarchical networks.

This appendix includes an exhaustive list of socially efficient twelve-node networks when where universal threshold contribution is a Nash equilibrium when $\min(k_{in}) = 1$ and $\max(k_{out}) > 1$ and each network has the maximum number of pure contributors. It also shows their associated hierarchy scores. The networks with the highest hierarchy scores for each network are shown in Figure 5 and the range of hierarchy scores is shown in Figure 6. Since each network in this appendix has $N$ links, the efficiency score for all of these networks is 0.9917.

$\max(k_{out}) = 2$

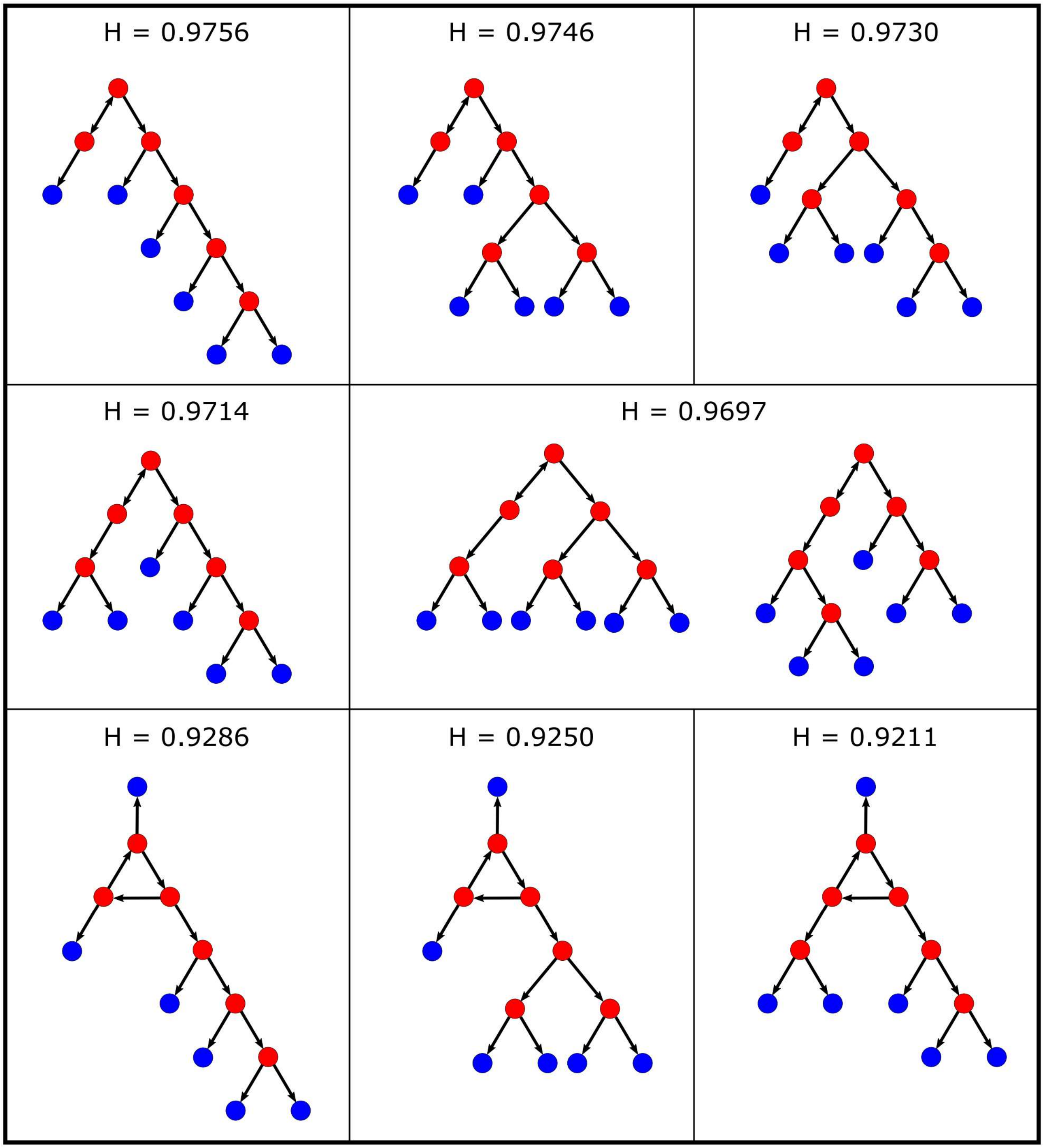


Figure S9: Nine socially efficient network structures where $\min(k_{in}) = 1$ and $\max(k_{out}) = 2$ and where the number of pure contributors (blue nodes) are maximized at six. Hierarchy scores for each network are shown at the top of each box. These are the nine networks with the highest hierarchy scores. The remainder are in Figure S10.

$\max(k_{out}) = 2$

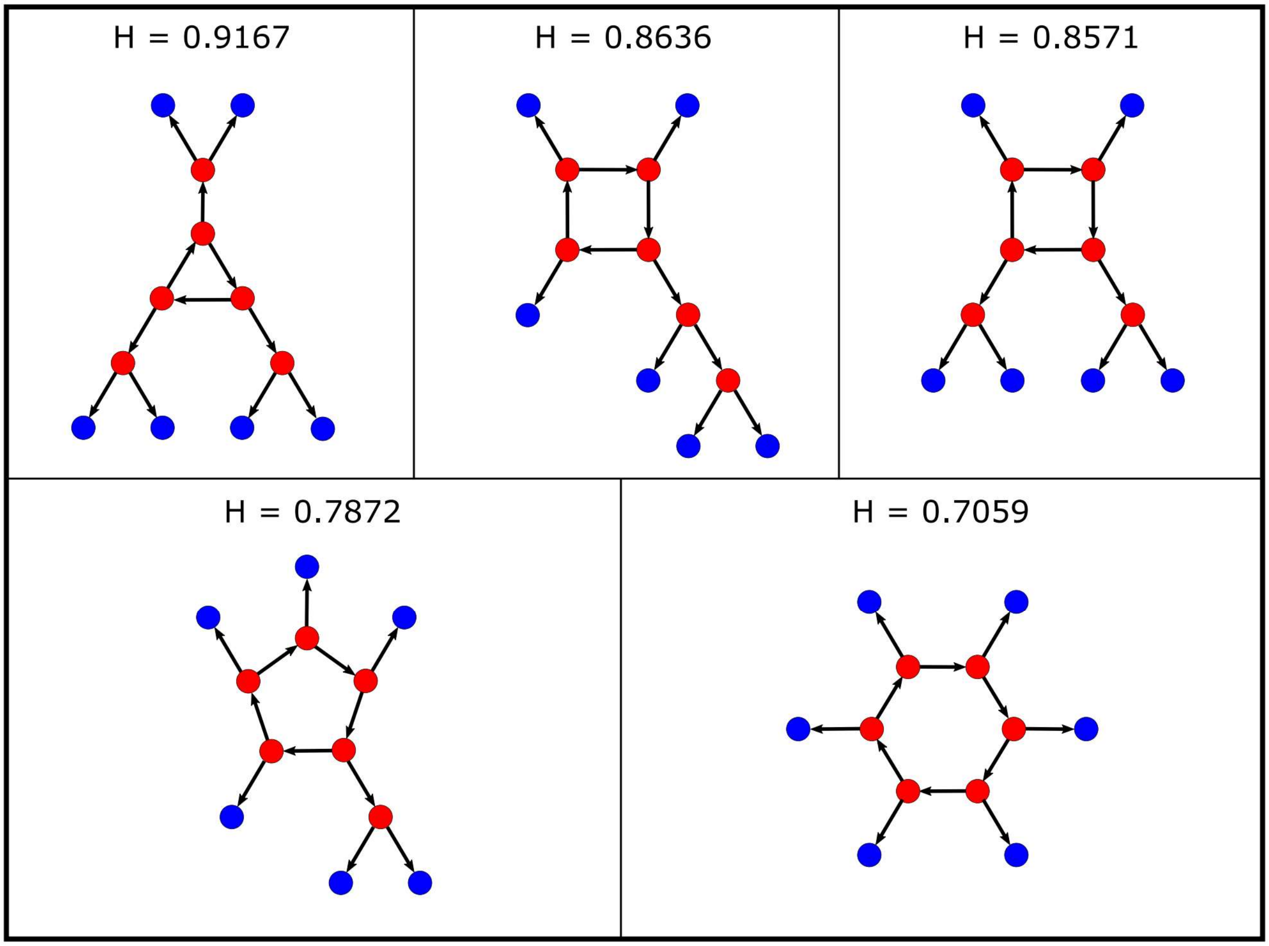


Figure S10: Five socially efficient network structures where $\min(k_{in}) = 1$ and $\max(k_{out}) = 2$ and where the number of pure contributors (blue nodes) are maximized at six. Hierarchy scores for each network are shown at the top of each box. These are the five networks with the lowest hierarchy scores. The remainder are in Figure S9.

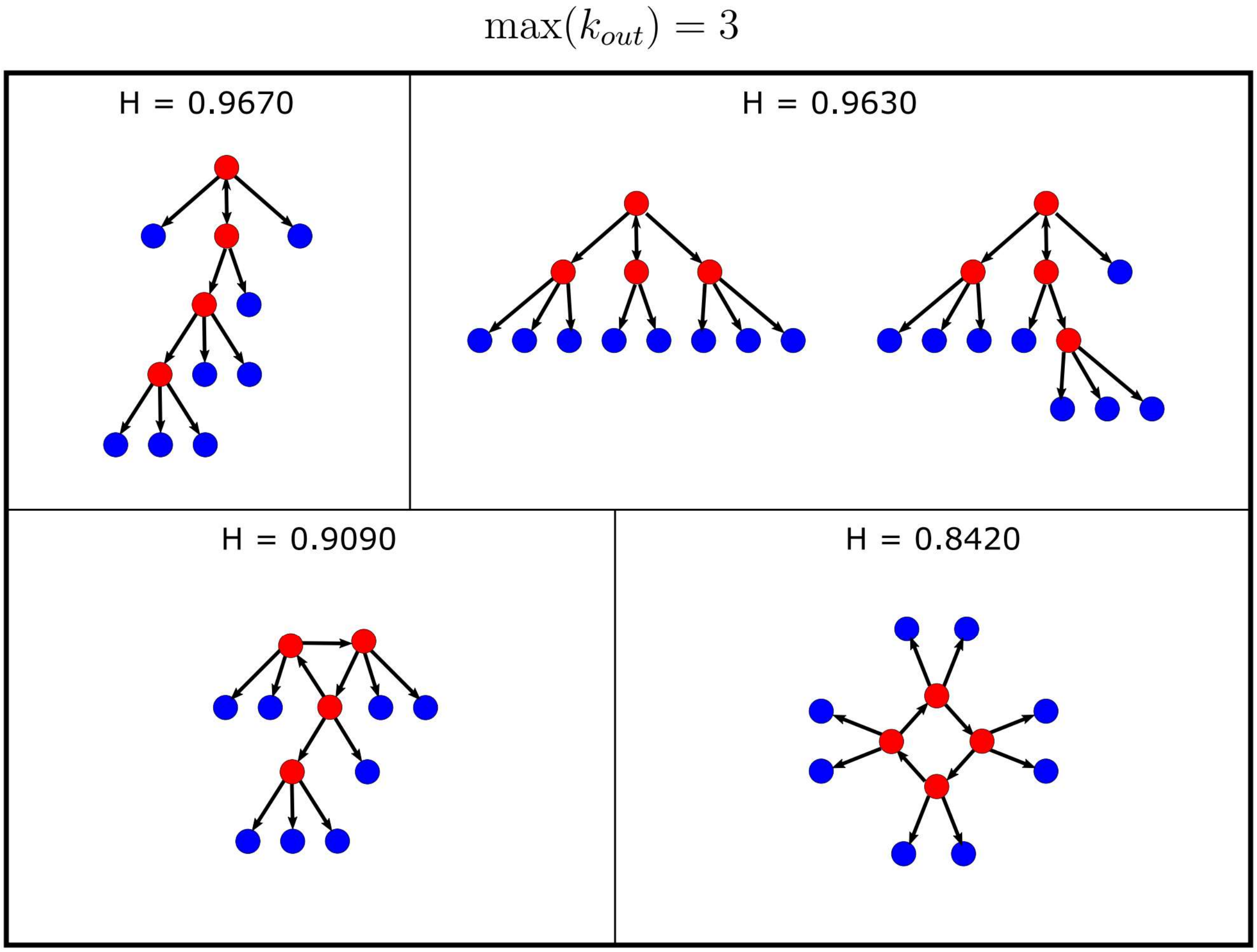


Figure S11: Five socially efficient network structures where $\min(k_{in}) = 1$ and $\max(k_{out}) = 3$ and where the number of pure contributors (blue nodes) are maximized at eight. Hierarchy scores for each network are shown at the top of each box.

$\max(k_{out}) = 4$

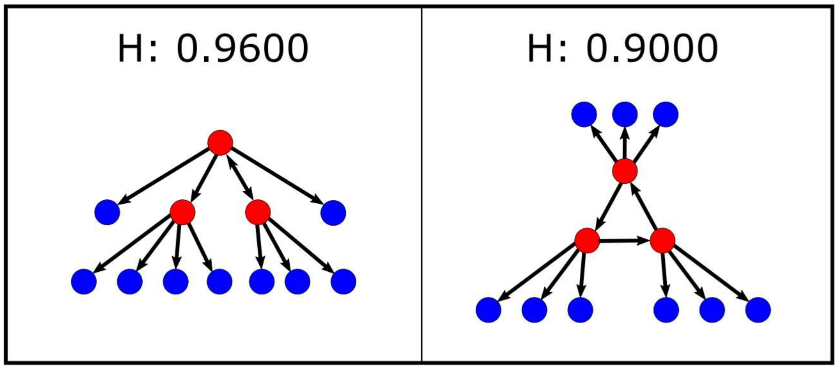


Figure S12: Two socially efficient network structures where $\min(k_{in}) = 1$ and $\max(k_{out}) = 4$ and where the number of pure contributors (blue nodes) are maximized at nine. Hierarchy scores for each network are shown at the top of each box.

$\max(k_{out}) = 5$

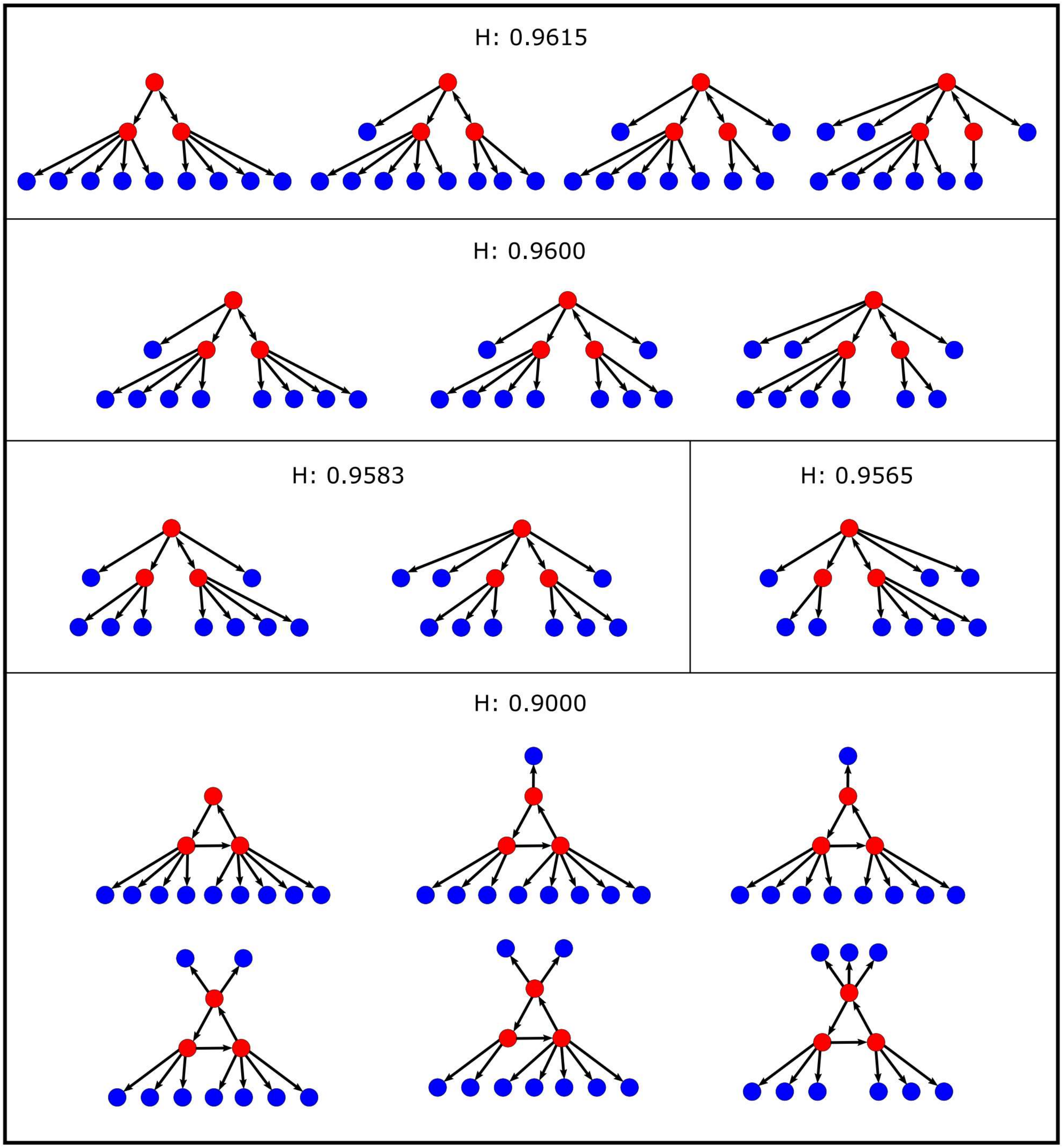


Figure S13: Seven socially efficient network structures where $\min(k_{in}) = 1$ and $\max(k_{out}) = 5$ and where the number of pure contributors (blue nodes) are maximized at none. All of these networks have the same graph efficiency scores, but they can have different graph hierarchy scores. Graph hierarchy scores are shown at the bottom of the box with each network. These networks include those in Figure S12 for $\max(k_{out}) = 4$.

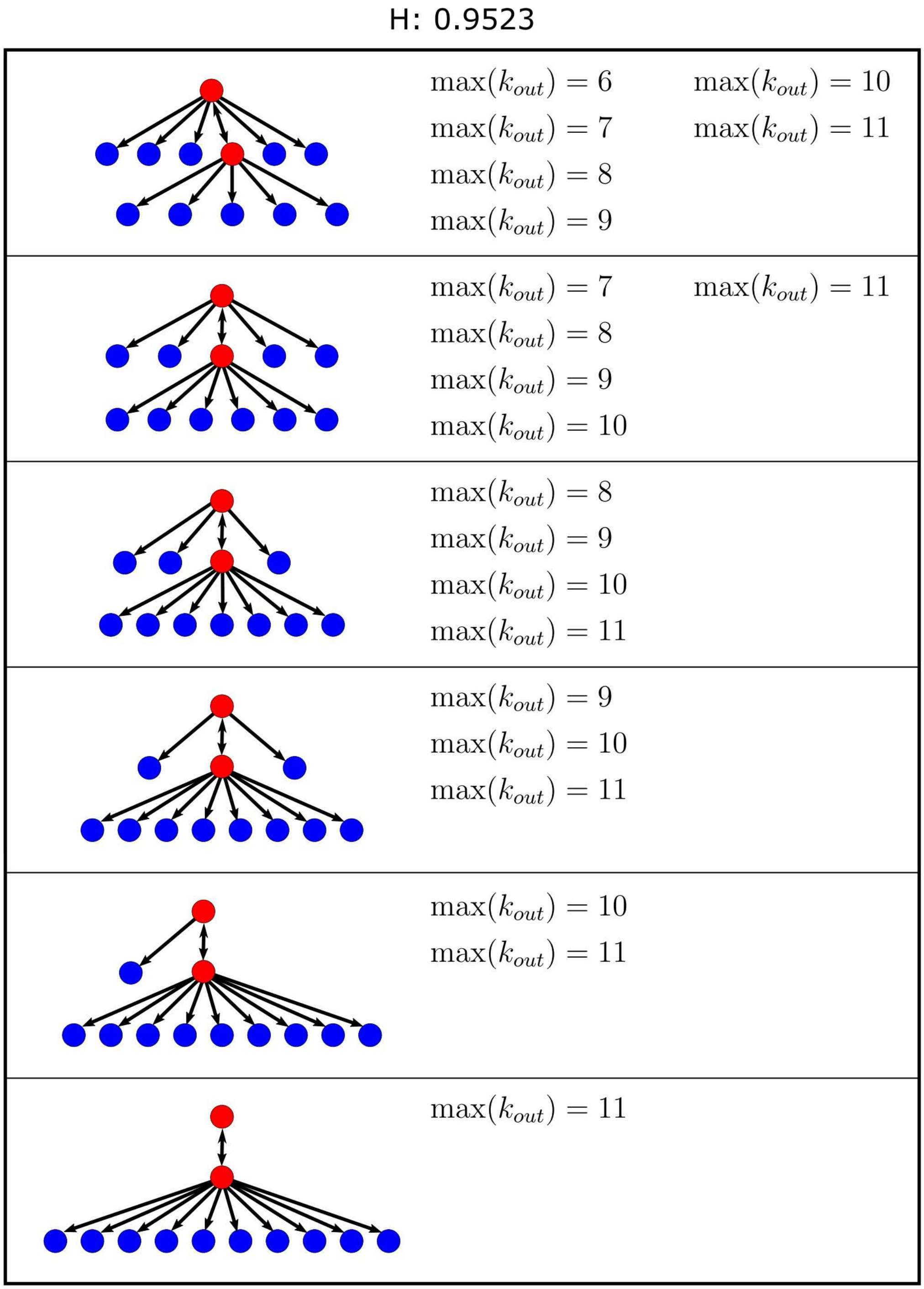


Figure S14: Seven socially efficient network structures where $\min(k_{in}) = 1$ and $\max(k_{out}) = 6$ to $\max(k_{out}) = 11$ and where the number of pure contributors (blue nodes) are maximized at ten. All of these networks have the same graph hierarchy score, $H = 0.9524$, the maximum span-of-control increases with $\max(k_{out})$. All of these network structures are possible when $\max(k_{out}) = 11$ but only the first is possible when $\max(k_{out}) = 6$.

# S14 Socially efficient 120-node networks

In the main text, I demonstrated the use of this model for understanding the relationship between collective action problem payoffs and smaller networks of six or twelve nodes. In this section, I show that a similar logic applies to larger networks, by exploring socially efficient networks of 120 nodes under different monitoring and punishment efficiencies when universal threshold contribution is a Nash equilibrium. Figure S15 shows a heat map of the graph efficiency and Figure S16 shows the graph hierarchy for these networks with different minimum $k_{in}$ and maximum $k_{out}$ thresholds. Consistent with the results for the smaller networks, graph efficiency is higher with greater monitoring efficiency, and graph hierarchy is greater with greater punishment efficiency.

To generate these plots one thousand random networks were generated in for each combination of $\min(k_{in})$ and $\max(k_{out})$ in the manner described for 12-node networks in Appendix S12. Graph efficiency and graph efficiency scores were measured for each network using the *sna* package. Networks used to generate Figures S15 and S16 are those with the highest graph hierarchy score.

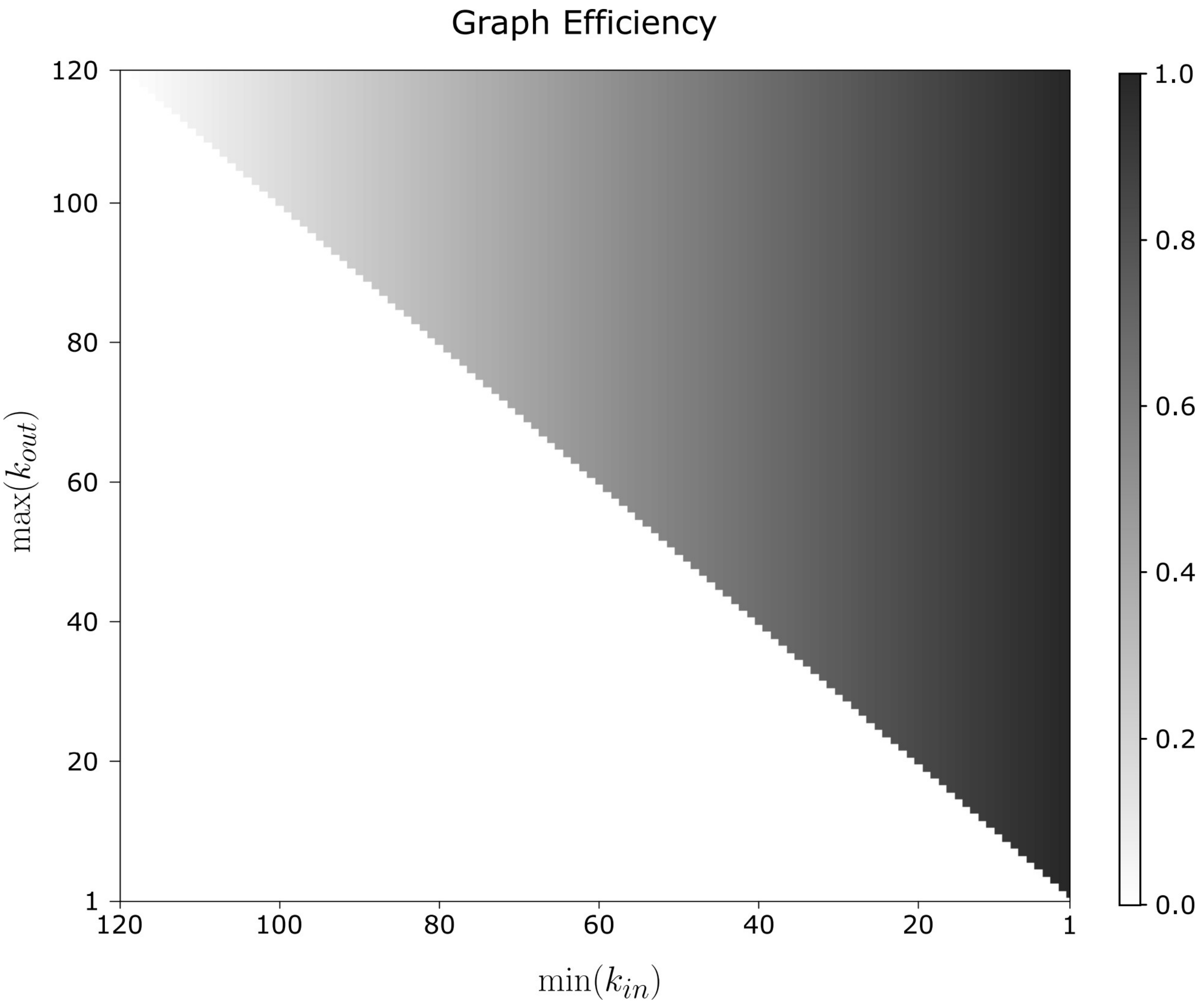


Figure S15: Graph efficiency scores for the most hierarchical socially efficient 120-node networks under different monitoring and punishment efficiencies when universal threshold contribution is a Nash equilibrium. As with the six-node and 12-node graphs in Figures S1 and 6, higher punishment efficiency corresponds to a lower minimum in-degree ($k_{in}$) for contribution to be a Nash for each individual and this results in a higher graph efficiency. Graphs on the far right of the plot resemble hierarchical out-trees.

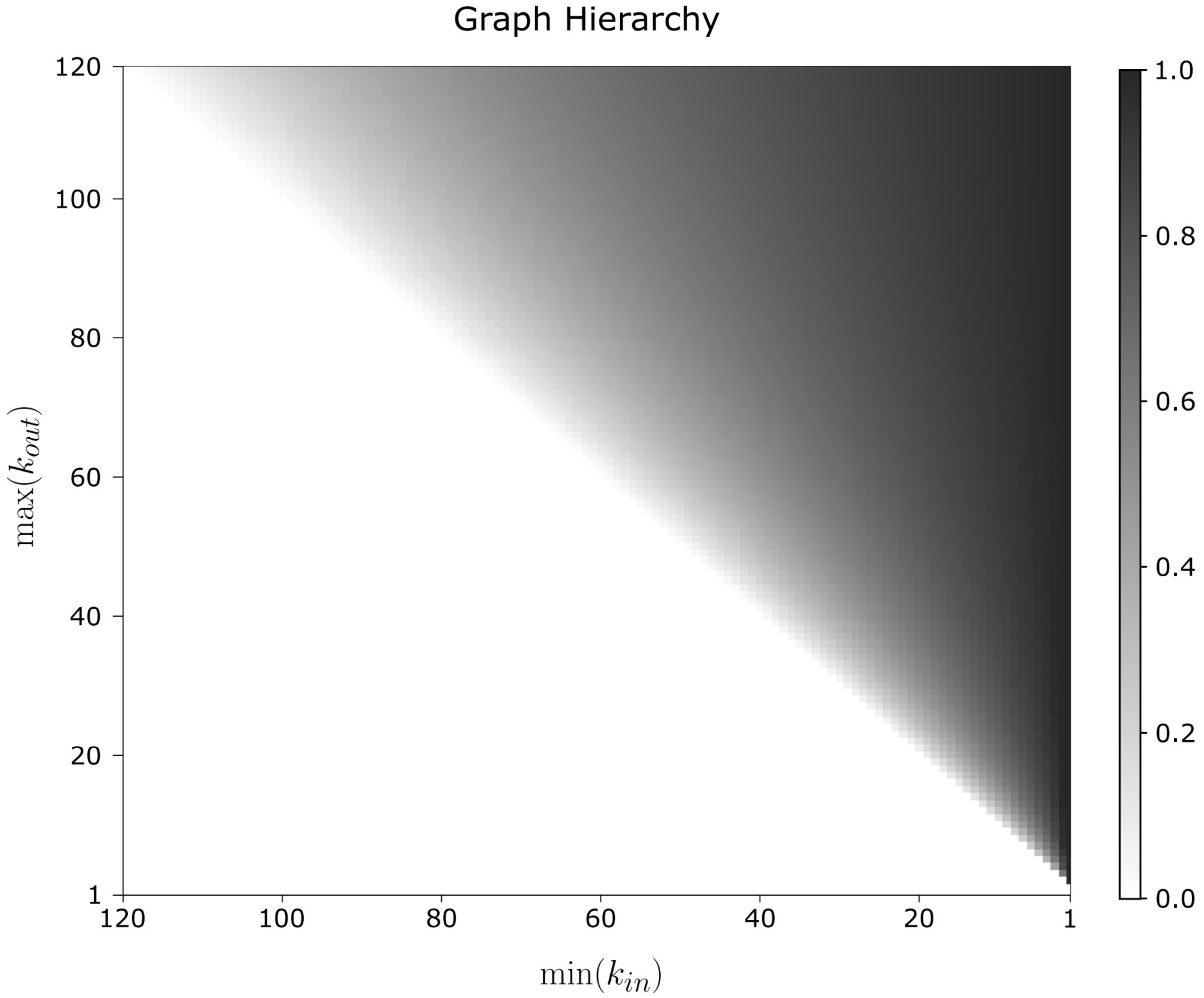


Figure S16: Graph hierarchies for the most hierarchical socially efficient 120-node networks under different monitoring and punishment efficiencies when universal threshold contribution is a Nash equilibrium. As with the six-node and 12-node graphs in Figures S1 and 6, higher monitoring and higher punishment efficiency correspond to higher graph hierarchy scores. Graphs on the far right of the plot resemble hierarchical out-trees.
